# A nanobody targeting the translocated intimin receptor inhibits the attachment of enterohemorrhagic *E*. *coli* to human colonic mucosa

**DOI:** 10.1371/journal.ppat.1008031

**Published:** 2019-08-29

**Authors:** David Ruano-Gallego, Daniel A. Yara, Lorenza Di Ianni, Gad Frankel, Stephanie Schüller, Luis Ángel Fernández

**Affiliations:** 1 Department of Microbial Biotechnology, Centro Nacional de Biotecnología, Consejo Superior de Investigaciones Científicas (CSIC), Campus UAM-Cantoblanco, Madrid, Spain; 2 MRC Centre for Molecular Bacteriology and Infection, Life Sciences Department, Imperial College London, London, United Kingdom; 3 Norwich Medical School, University of East Anglia, Norwich Research Park, Norwich, United Kingdom; 4 Quadram Institute Bioscience, Norwich Research Park, Norwich, United Kingdom; McMaster University, CANADA

## Abstract

Enterohemorrhagic *E*. *coli* (EHEC) is a human intestinal pathogen that causes hemorrhagic colitis and hemolytic uremic syndrome. No vaccines or specific therapies are currently available to prevent or treat these infections. EHEC tightly attaches to the intestinal epithelium by injecting the intimin receptor Tir into the host cell via a type III secretion system (T3SS). In this project, we identified a camelid single domain antibody (nanobody), named TD4, that recognizes a conserved Tir epitope overlapping the binding site of its natural ligand intimin with high affinity and stability. We show that TD4 inhibits attachment of EHEC to cultured human HeLa cells by preventing Tir clustering by intimin, activation of downstream actin polymerization and pedestal formation. Furthermore, we demonstrate that TD4 significantly reduces EHEC adherence to human colonic mucosa in *in vitro* organ cultures. Altogether, these results suggest that nanobody-based therapies hold potential in the development of much needed treatment and prevention strategies against EHEC infection.

## Author summary

Currently, there is no effective treatment or vaccine against enterohemorrhagic *E*. *coli* (EHEC), a bacterial pathogen that infects human colon after the ingestion of contaminated food. It thrives in the colon thanks to its ability to attach intimately to the intestinal epithelium. Here, we have identified and characterised a small antibody fragment (nanobody) that recognises Tir, a receptor injected by the bacterium into the host cell to mediate intimate attachment. This nanobody shows higher affinity against Tir than its natural bacterial ligand (intimin) and, most importantly, blocks the intimate attachment of the pathogen to the human colonic tissue. Our results show the potential of this nanobody to prevent and treat EHEC infection.

## Introduction

Enterohemorrhagic *E*. *coli* (EHEC) is a major public health concern in industrial countries with most severe infections linked to serotype O157:H7. In addition to diarrhoea, EHEC can cause hemorrhagic colitis as well as life-threatening hemolytic uremic syndrome (HUS) damaging the kidneys and central nervous system [1–4]. EHEC naturally resides in the intestinal tract of cattle, and most infections are acquired by consumption of undercooked beef products or cross-contaminated vegetables or sprouts [5]. Upon infection, EHEC adheres to the epithelium of the distal ileum and colon by forming attaching and effacing (A/E) lesions, which are characterized by intimate bacterial attachment and effacement of the brush border microvilli [6, 7]. This is mediated by the Locus of Enterocyte Effacement (LEE) [8], a pathogenicity island encoding a filamentous type III secretion system (T3SS) [9, 10], the outer membrane adhesin intimin and the translocated intimin receptor (Tir), and other effector proteins involved in pathogenesis [11, 12].

After formation of the translocation filament consisting of EspA proteins, Tir is injected into intestinal epithelial cells (IECs), where it integrates into the plasma membrane in a hairpin loop topology, presenting an extracellular domain of about 100 residues (TirM) [13, 14] that serves as a binding site for the C-terminal lectin-like domain of intimin [15–17]. Binding of intimin to Tir leads to intimate bacterial attachment, Tir clustering, activation of actin polymerization pathways and subsequent formation of actin pedestals and A/E lesions [7, 18–22].

Other key virulence factors of EHEC are the phage-encoded Shiga toxins (Stx) which are released into the bloodstream and cause the systemic effects associated with HUS [23, 24]. So far, there is no specific treatment for HUS, and application of antibiotics is discouraged as it induces Stx expression and thereby increases the risk of developing HUS [25, 26]. Therefore, there is a need to develop alternative therapies, and the use of antibodies (Abs) has been proposed for treatment of infectious diseases [27]. In particular, members of the family *Camelidae* (e.g. dromedaries, llamas) produce a class of Abs devoid of light chains [28, 29]. In these heavy-chain-only Abs, the antigen-binding site is formed by a single variable domain termed V_HH_ [30]. The recombinant expression of camelid V_HH_s yields single domain Ab fragments, which are also referred to as nanobodies (Nbs) [31]. The V_HH_s have extended complementarity determining regions (CDRs) capable of adopting novel conformations and recognizing epitopes located in otherwise non-accessible clefts or protein cavities, such as active sites of enzymes [32, 33] and inner regions of surface proteins from pathogens [34]. They also show strict monomeric behavior, reversible folding properties, higher resistance to proteolysis and thermal degradation, when compared with the variable domains of conventional antibodies [31, 35, 36]. In addition, the high similarity between V_HH_s and human VH3 sequences opens their potential use in therapeutic applications [31]. These beneficial properties offer opportunities to use Nbs for the development of therapeutic inhibitors against extracellular pathogens [37, 38].

We have previously isolated a set of Nbs binding to EspA, the C-terminal receptor-binding domain of intimin (Int280) and the TirM domain from a library of V_HH_s obtained after immunization of a dromedary (*Camelus dromedarius*). Nanobodies were secreted to the extracellular medium using the hemolysin (Hly) transport system of *E*. *coli* and purified from the culture supernatants [39]. Here, we have investigated the ability of the selected Nbs to inhibit EHEC adhesion to HeLa cells and human colonic mucosa. We have identified a Nb clone that binds TirM, named TD4, which reduces the interaction of TirM with Int280 and interferes with actin pedestal formation and the intimate attachment of EHEC to human cells. Importantly, using infection of human *in vitro* organ cultures (IVOC), we demonstrate that Nb TD4 can also inhibit the attachment of EHEC to human colonic tissue.

## Results

### Nanobodies against TirM inhibit EHEC attachment to HeLa cells

**Fig 1:**
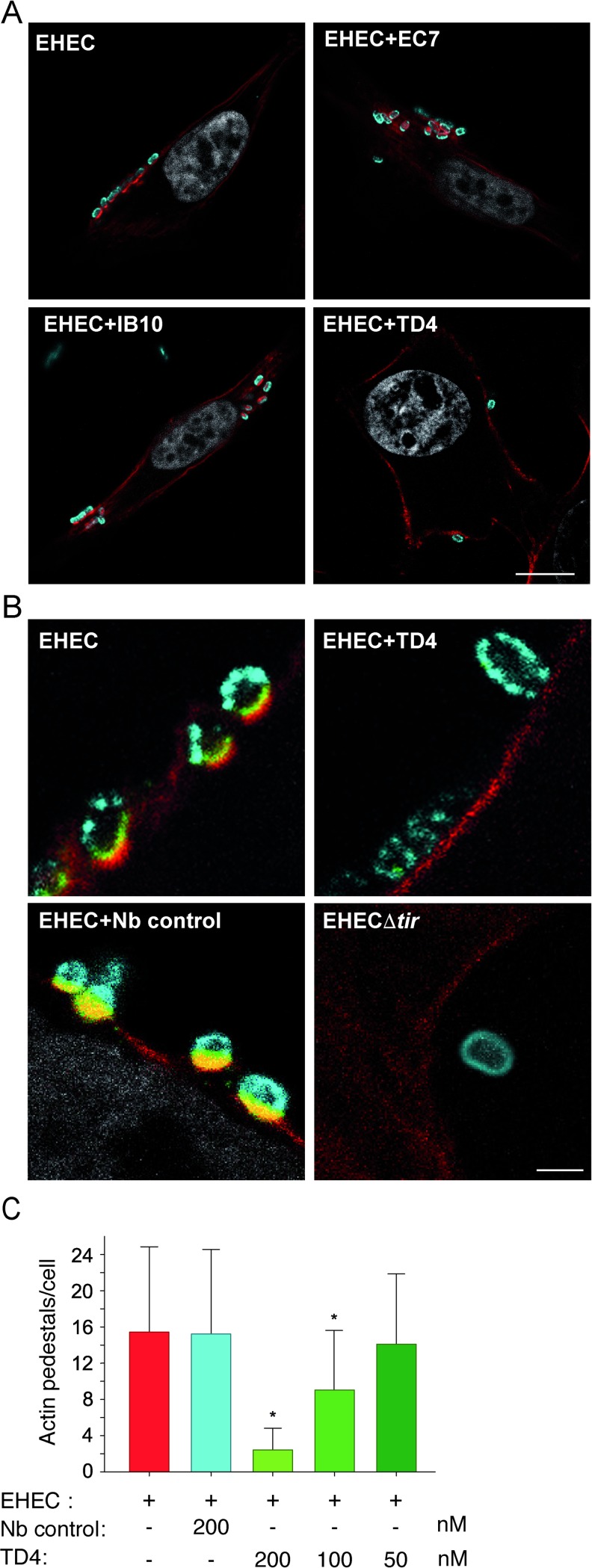
Influence of Nbs on EHEC adhesion to HeLa cells and Tir clustering. **A**. Confocal microscopy images of HeLa cells infected with EHEC for 3 h in the presence of 200 nM purified Nbs against EspA (EHEC+EC7), Int280 (EHEC+IB10), TirM (EHEC+TD4), control Nb or medium control (EHEC), as indicated. Cells were stained for EHEC (cyan), actin (red) and cell nuclei (white). Bar = 7.5 μm. **B.** Tir clustering underneath EHEC bacteria in the presence of Nb TD4. EHEC infection of HeLa cells for 3 h in the presence of media control, 200 nM Nb TD4 or Nb control, as indicated. EHECΔtir strain was used as negative control. Cells were stained for EHEC (cyan), Tir (green), actin (red) and cell nuclei (white). Bar = 1 μm. **C**. Actin pedestal formation per cell was quantified by counting 100 cells per condition. Data are presented as means and standard deviations. * p < 0.05 versus EHEC control.

To determine if purified Nbs against EspA, Int280 and TirM affected EHEC A/E lesion formation, HeLa cells were infected with EHEC for 3 h in the presence or absence of Nbs. Actin pedestal formation was visualized and quantified by immunofluorescence staining. While EHEC attachment and pedestal formation was not affected by Nb clones recognizing EspA (EC7) or Int280 (IB10) at concentrations 200 nM (Fig 1A), a Nb clone binding TirM (TD4) significantly reduced the accumulation of actin beneath the attached bacteria. As clustering of Tir is necessary for A/E lesion formation, we also evaluated the Tir localization in the presence of Nb TD4 or an unrelated Nb. As shown in Fig 1B, localization of Tir beneath adherent EHEC was evident in samples incubated with the control Nb or non-treated controls, while no Tir accumulation was observed in the presence of TD4. No Tir staining was detected in HeLa cells infected with EHECΔ*tir*. We quantified the effect of TD4 by determining the mean of pedestals formed on infected cells under various concentrations of Nb TD4. This revealed a significant decrease in the number of actin pedestals per infected cell when TD4 Nb was added at concentrations ≥ 100 nM (Fig 1C).

**Fig 2:**
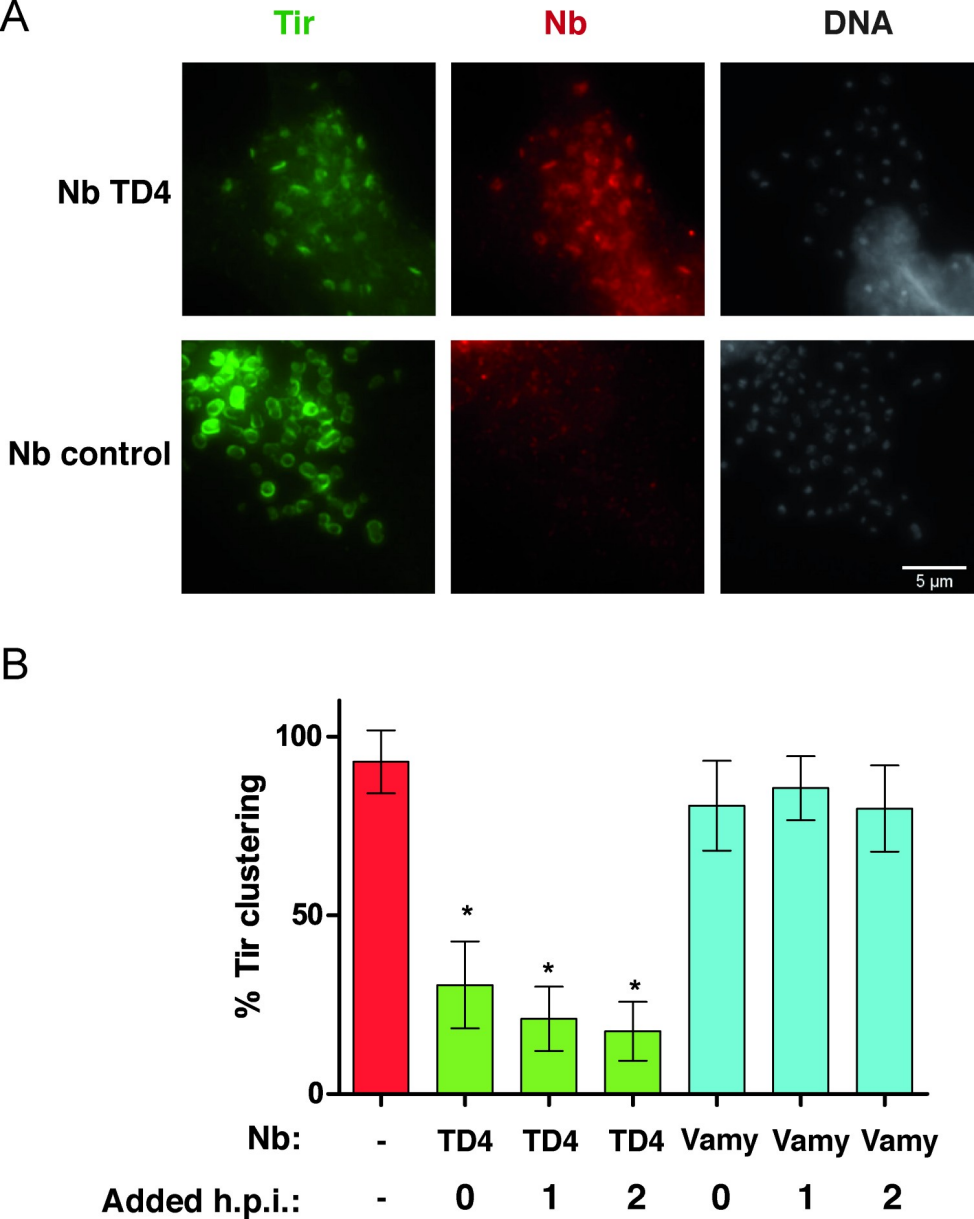
Visualization of TD4-Tir colocalization and effect of its addition after the beginning of the infection. **A**. Images of HeLa cells infected with EHEC for 3 h in the presence of 200 nM of TD4 or Nb control (Vamy). Cells were stained for Tir (green), HA-tag (red) and DAPI (gray). Bar = 5 μm. **B**. Nbs were added at the beginning of the infection (0 h), 1 h or 2 h post infection (h.p.i.)., as indicated. The number of Tir clusters was put in relation with the total amount of bound bacteria per cell, for 50 cells randomly selected in different microscopic fields of the sample. * p < 0.05 versus EHEC control.

We wanted to rule out the possibility that the lack of Tir clustering beneath the bound bacteria could be due to a block of Tir translocation through the T3SS and not to the direct interaction of the Nb TD4 to the exposed region of Tir upon its translocation and insertion in the plasma membrane of the host cell. We tested this possibility and simultaneously evaluated whether TD4 can interfere with EHEC actin-pedestal formation when added at different times during infection. To this end, we increased the infection rate by halving the volume of the medium and added 200 nM of Nbs TD4 or control (Vamy) simultaneously with the infection, or at 1 or 2 h post-infection. After a total of 3 h of infection, all samples were stained for Tir and the HA-tagged Nbs to test for their co-localization (Fig 2). Due to the higher infection rate, some Tir signal could be observed beneath the bound bacteria with TD4, but the arrangement of Tir staining in the host cells was altered, being distributed in the cytoplasm and showing only weak staining marks at the site of the EHEC adhesion (Fig 2A). Furthermore, we could detect colocalization of the HA-tagged TD4 with Tir, showing that the interaction between TD4 and Tir occurs at the surface of infected host cells, once Tir has been translocated, and suggesting that this interaction is responsible for the observed phenotype. In contrast, infections incubated with the control Nb (Vamy) showed strong Tir signals accumulated beneath EHEC bacteria and no staining of the cytoplasm (Fig 2A). Importantly, this inhibitory effect of TD4 was observed at similar levels independently of the time of addition of the Nb (0, 1 or 2 h post-infection) as determined by quantification of Tir clusters in the infected cells (Fig 2B).

**Fig 3:**
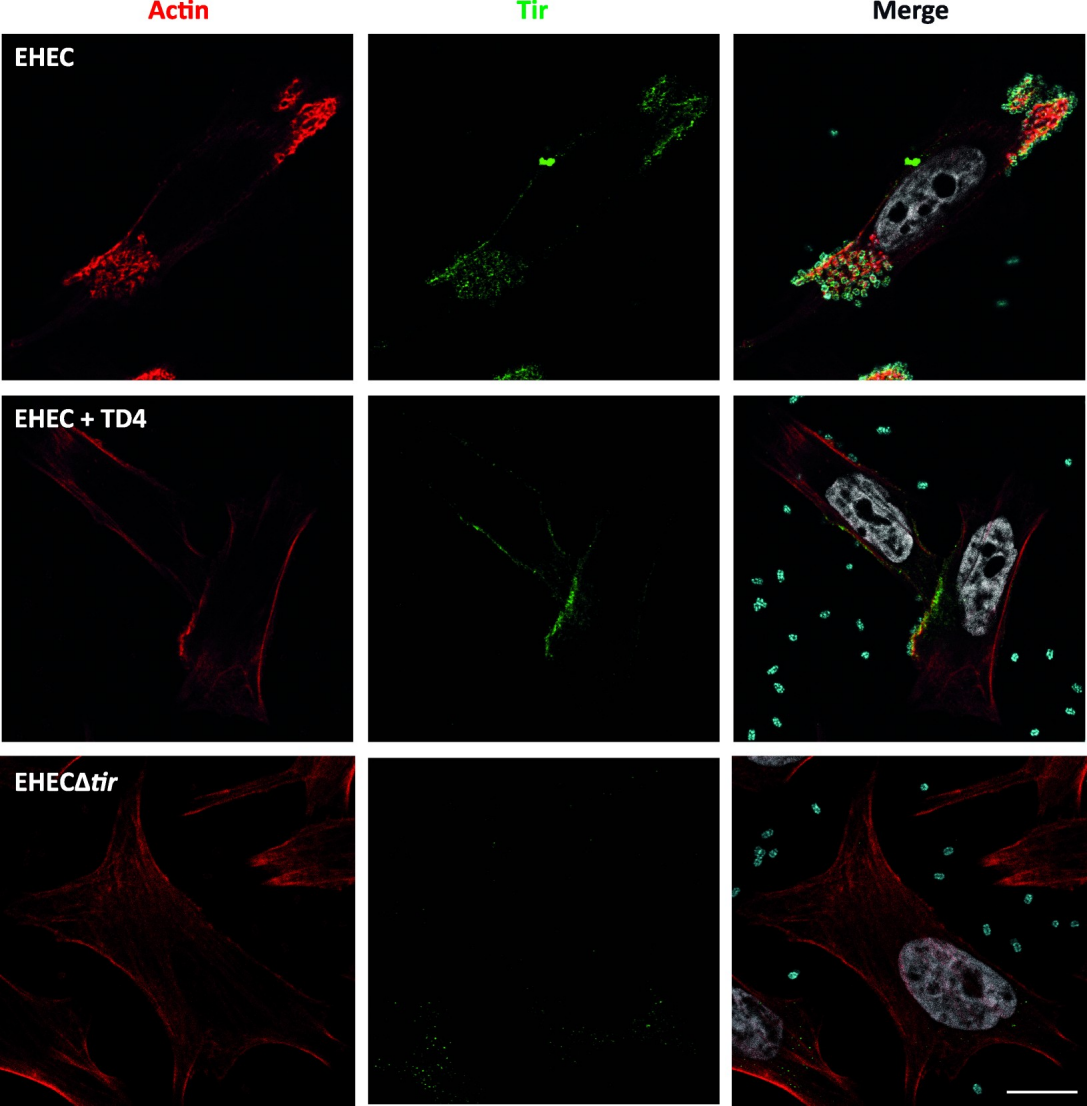
Effect of TD4 after 6 h-long infection of HeLa cells with EHEC. Images of sample slices were taken at the confocal microscope after 6 h of infection. The upper panels show the infection control of wt EHEC; the middle panels, the effect of TD4 (200 nM) to this infection; and the panel in the bottom, the infection of the EHECΔ*tir* mutant. Fluorescent-staining of F-actin is shown in red, the O157 antigen of EHEC in cyan, TirM of EHEC in green and DNA in gray. Bar = 7.5 μm.

Lastly, we assessed the inhibitory effect of TD4 at longer times of infection. HeLa cells were infected with EHEC for 6 h in the presence or absence of TD4, and stained for bacteria, Tir and F-actin. In this experiment fresh medium and Nbs were added after 3 h of infection. Inspection of these samples revealed the presence of a high number of intimately attached EHEC bacteria and dense clusters of Tir in the absence of TD4 (Fig 3). The high density of EHEC bacteria did not allow us to visualize individual bacteria with Tir clustering for quantification purposes. Nonetheless, we clearly observed that the presence of TD4 dramatically reduced the number of EHEC bound to HeLa cells, as well as the intensity of actin and Tir signals in those bacteria that were bound to the cells (Fig 3). Taken together, the above data showed that Nb TD4 reduces EHEC attachment, Tir clustering and actin polymerization by binding to the extracellular TirM domain exposed after Tir translocation. The Nb TD4 shows this inhibitory activity even when added once infection has begun.

### Nb TD4 has a higher affinity for TirM than its natural ligand Int280

**Fig 4:**
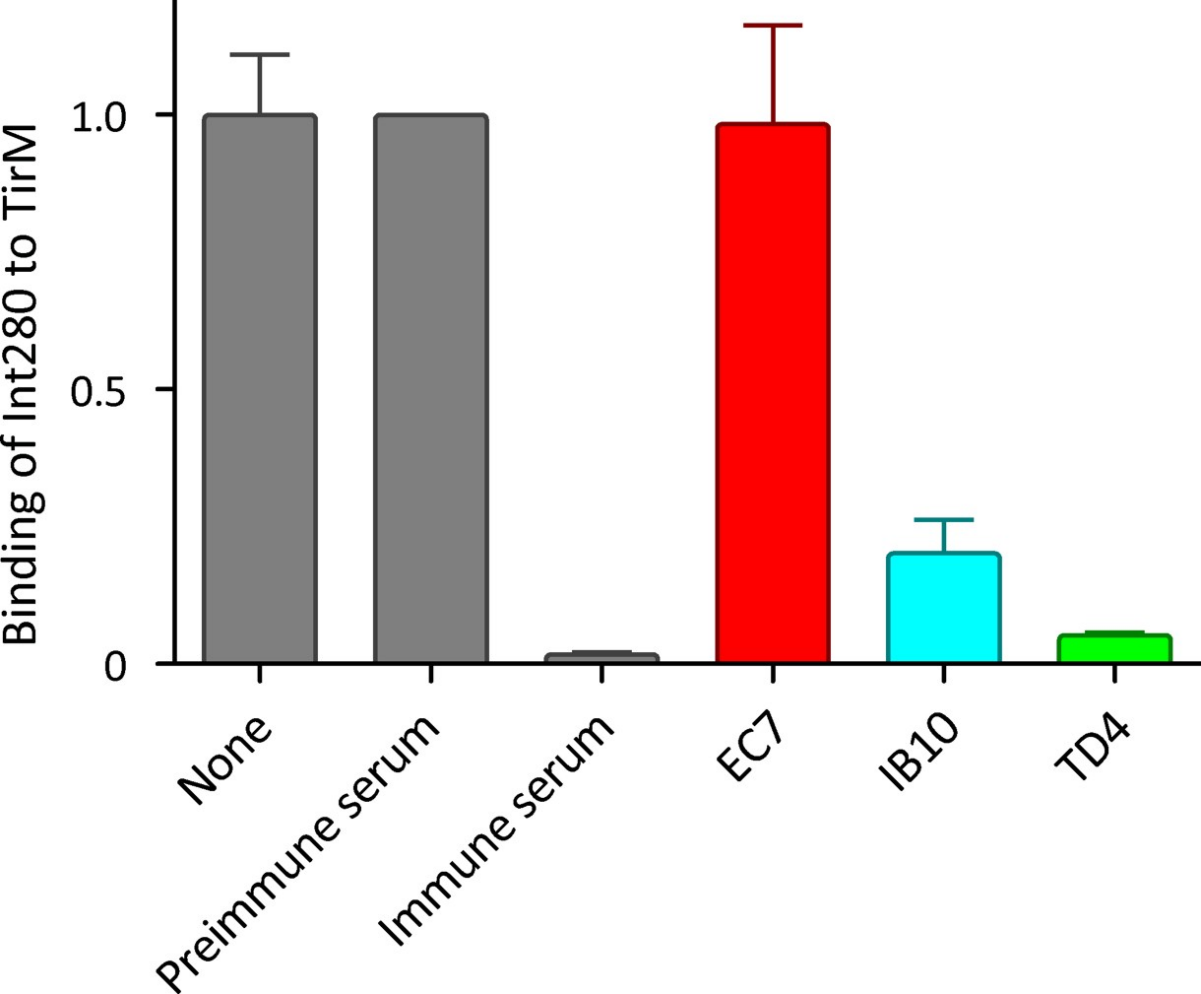
Competition of purified Nbs for TirM binding in the presence of Int280. Representation of the binding signals obtained from Int280-TirM interaction ELISA in the presence of PBS (none), the indicated camel sera or purified Nbs (EC7, IB10 and TD4). ELISA plates were coated with 5 μg/ml TirM_EHEC_ or BSA control and incubated with 50 μg/ml biotinylated Int280_EHEC_ in the presence of camel pre-immune and immune sera (1:50 dilution), 50 μg/ml purified Nbs (EC7, IB10, TD4) or PBS control. Binding of biotinylated Int280 was evaluated by incubation with Streptavidin-POD and measurement of OD490. Data are presented as means and standard deviations from of two independent experiments with triplicates. Values are indicated relative to those of Int280-TirM interaction in the presence of PBS.

One mechanism by which Nb TD4 could interfere with the attachment of EHEC to human cells is by directly competing with intimin for binding TirM. To investigate this, ELISA plates coated with purified TirM were incubated with biotinylated Int280 in the presence of different Nbs (Fig 4). While Int280:TirM interaction was not affected by the presence of camel pre-immune serum or Nb EC7 binding EspA (control), incubation with camel immune serum or Nb TD4 inhibited the interaction. In addition, Nb IB10 (anti-Int280) also reduced Int280:TirM interaction, but to a lesser extent than TD4. These results show that Nb TD4 is a potent inhibitor of Int280-TirM interaction.

**Fig 5:**
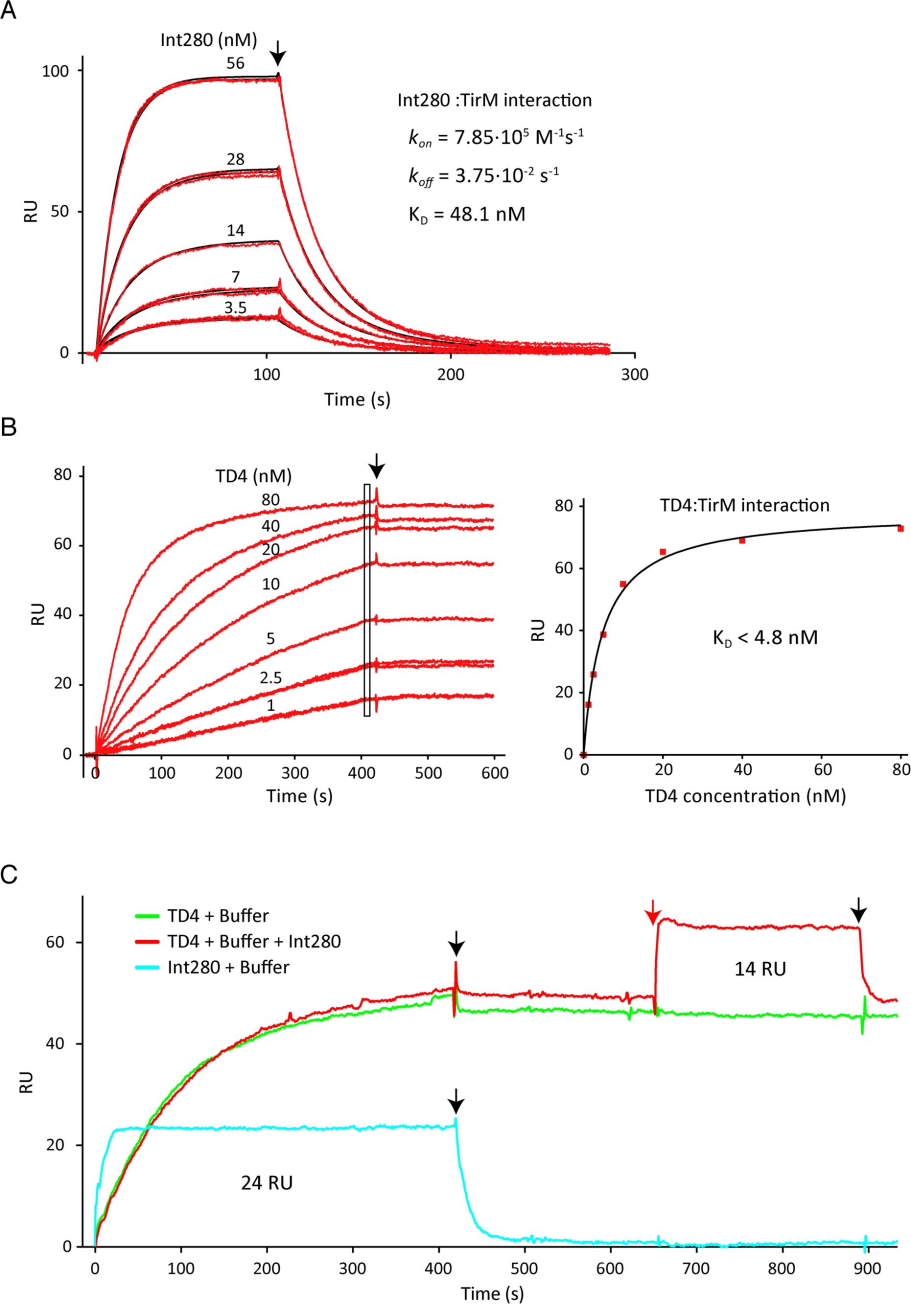
SPR analysis of TD4 and Int280 binding to TirM. **A**. Change in resonance units (RU) with time (sensograms) obtained with purified Int280_EHEC_ (at the indicated concentrations) passed through a streptavidin chip coated with biotinylated TirM_EHEC_. Sensograms were used to calculate the kinetic constants of association (k_*on*_) and dissociation (k_*off*_), and the equilibrium dissociation constant (K_D_) using the BiaEvaluation software. The curves were fitted to a model 1:1 Langmuir interaction (black lines) for the calculation. **B**. Sensograms obtained with purified TD4-HlyA (at the indicated concentrations) passed through a streptavidin chip coated with biotinylated TirM_EHEC_. The absence of significant dissociation preclude the calculation of kinetic constant of dissociation (k_*off*_). The RU shift near the steady state (at 400 s, indicated with a rectangle) were used to estimate an apparent constant of dissociation (K_D_) of TD4-HlyA for TirM_EHEC_ representing the shift in RU values *vs* concentration of TD4-HlyA (graph on the right). **C.** Sensograms showing binding to TirM_EHEC_ by 40 nM of TD4 followed by HEPES buffer (green line) or 40 nM TD4 followed by 80 nM Int280_EHEC_ (red line). The blue line represents the sensogram obtained by 80 nM Int280_EHEC_ (without TD4) followed by HEPES buffer. The black arrows indicate the time of injection of HEPES buffer. The red arrow indicates the time of injection of Int280_EHEC_ in the chip in which TD4 was previously injected.

To further characterize the binding of Nb TD4 to TirM and its inhibitory activity, we compared the affinities of Int280 and TD4 for TirM using surface plasmon resonance (SPR). Biotinylated TirM was immobilized onto a chip for SPR, and purified Int280 and TD4 Nb-HlyA fusion were passed over the chip at different concentrations in successive rounds of binding and regeneration. The change in resonance units (RU) with time was recorded as a direct indication of the binding of these proteins to TirM. The sensograms obtained are represented in Fig 5. These experiments revealed a distinct pattern of binding of Int280 and TD4 to TirM. While Int280 quickly bound to and dissociated from TirM after stopping Int280 injection (Fig 5A), TD4 bound to TirM more slowly and the interaction remained stable without any detectable dissociation even >300 sec after the injection stopped (Fig 5B). The kinetic constants of association (*k*_*on*_) and dissociation (*k*_*off*_) of Int280-TirM binding could be calculated directly from the obtained sensograms (Fig 5A). A model 1:1 Langmuir interaction fitted the binding curves, suggesting the formation of a 1:1 complex, as observed by protein crystallography of the EPEC Int280:TirM complex [15]. Using this binding model, we determined a *k*_*off*_ of 3.75·10^−2^ s^-1^ and a *k*_*on*_ of 7.85·10^5^ M^-1^s^-1^ for EHEC Int280:TirM interaction. The equilibrium dissociation constant (K_D_) for EHEC Int280-TirM interaction was calculated from the ratio of these kinetic constants (*k*_*off*_/*k*_*on*_) and determined to be 48.1 nM. In contrast to Int280, the fact that TD4 had no detectable dissociation of TirM during SPR analysis impeded the determination of its kinetic constants *k*_*on*_ and *k*_*off*_ from the obtained sensograms. In addition, the K_D_ could not be determined from RU values at equilibrium since the steady state was only reached at the highest concentration of TD4 (Fig 5B). Using the RU values closer to an apparent binding plateau at the different concentrations tested, we could estimate an apparent K_D_ ~4.8 nM for the TD4:TirM interaction (Fig 5B). The actual K_D_ for this interaction is likely to be below this estimated value (K_D_ < 4.8 nM) as the actual steady state would be reached with higher RU values. Hence, this quantitative binding analysis indicated an at least 10-fold higher affinity of TD4 for TirM than the one of its natural ligand, Int280.

Using SPR we also investigated whether TD4 recognised an epitope of TirM overlapping the binding site of Int280, taking advantage of the extremely slow dissociation of TD4. We injected 40 nM of TD4 into the TirM-chip until reaching RU values close to steady state followed by 80 nM of Int280 (Fig 5C). We compared the increment of RU values obtained by Int280 injection in this condition (with bound TD4) with those obtained by injecting the same concentration of Int280 to the TirM-chip in the absence of TD4. This experiment showed that the RU values of Int280 binding to TirM were reduced in the presence of TD4, but binding of Int280 occurred simultaneously to TD4, indicating that the binding sites of Int280 and TD4 are not identical, although they could partially overlap. Interestingly, when Int280 injection was stopped, Int280 quickly dissociated whereas TD4 remained bound to TirM and the RU in the assay came back to those of TD4 binding alone. Thus, while Int280 quickly dissociates from TirM, TD4 remains stably bound to it.

### Mapping the Nb TD4 binding site in TirM

**Fig 6:**
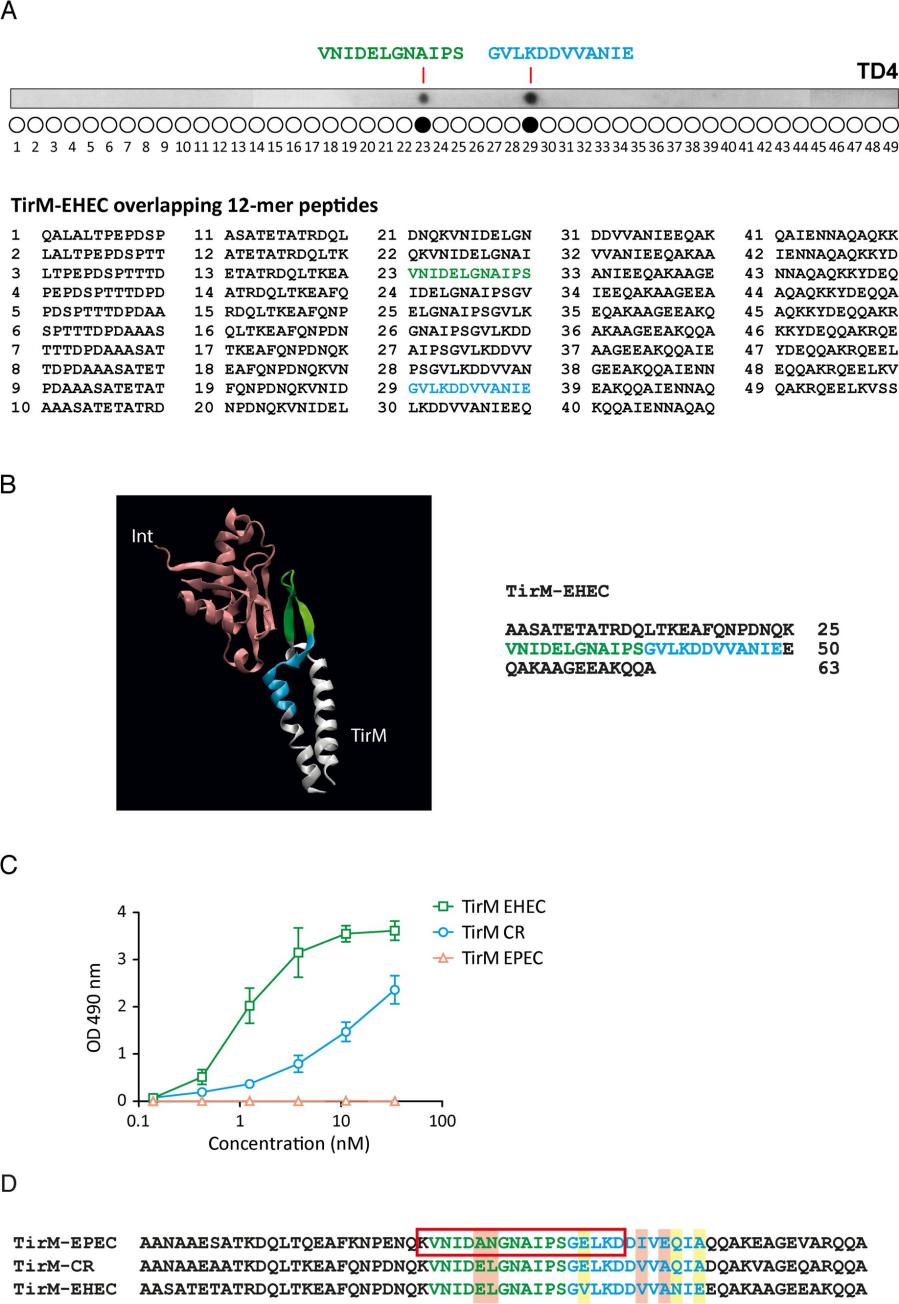
Identification of the TirM sequence recognised by Nb TD4. **A**. 12-mer peptides overlapping 10 residues and reconstructing the sequence of TirM_EHEC_ were synthesized on a PVDF membrane and incubated with purified TD4-HlyA. Bound TD4-HlyA was detected with anti-E-tag mAb as for Western blots. **B.** Structure of the crystal 1F02 (PDB) showing the interaction site of Int D3 domain (in pink) and Tir (in white) of EPEC. The peptides of TirM_EHEC_ recognized by TD4 are coloured in green and blue both in the crystal representation and in the linear sequence of the TirM_EHEC_. **C**. ELISA of purified TD4-HlyA (at the indicated concentrations) against TirM of EPEC, EHEC and CR. **D.** Alignment of TirM sequences of EHEC, EPEC and CR. In green and blue are coloured the peptides recognized by TD4-HlyA in EHEC. Inside the red rectangle are the identified aas for the Int:Tir interaction crystal 1F02. Highlighted in red are the residues that differ between EPEC TirM and the TirM of EHEC and CR. Highlighted in yellow are the residues that differ between CR TirM and EHEC TirM.

To identify the specific binding site of TD4 to TirM, we synthesized 12-mer peptides of EHEC TirM covering its sequence, with a 10 amino acid (aa) overlap between consecutive peptides on a PVDF membrane. After incubation with TD4, bound Nb was subsequently detected with a secondary antibody. This identified two peptides recognized by TD4: VNIDELGNAIPS (aa 296–307) and GVLKDDVVANIE (aa 308–319) (Fig 6A). These consecutive peptides are localized within the interaction interface between Int280 and Tir (Fig 6B) [15–17, 40]. A BLASTP search [41] with non-redundant DNA sequences in databases (S1 Data) allowed us to determine that the 24-mer sequence of these peptides is 100% identical (BLASTP score 77.4 in S1 Data) in Tir proteins from all EHEC strains, including O157, O55, O145 and other relevant non-O157 serotypes [42].

The sequence of TirM is highly conserved among the related A/E pathogens EHEC, enteropathogenic *E*. *coli* (EPEC) and *Citrobacter rodentium* (CR) but the sequence of these Tir peptides is not identical in EPEC and CR strains, so we were therefore interested to determine the affinity of TD4 to purified TirM_EPEC_ and TirM_CR_. We found that TD4 bound TirM_CR_ with lower affinity (ca. 10-fold) than TirM_EHEC_. Surprisingly, no binding of TD4 to TirM_EPEC_ was detected (Fig 6C). Comparing the aa sequences of the TD4 binding site in TirM_EHEC_ with corresponding regions in TirM_EPEC_ and TirM_CR_ (Fig 6D) revealed that TirM_EHEC_ differs from both TirM_EPEC_ and TirM_CR_ in residues V309, N317 and E319, suggesting that these changes may affect the affinity of TD4 towards TirM. Moreover, TirM_EPEC_ specifically differs from TirM_EHEC_ in residues E300, L301, V314 and A316, suggesting that these residues may be essential for TirM recognition by TD4.

### TD4 inhibits EHEC binding to human colonic mucosa

**Fig 7:**
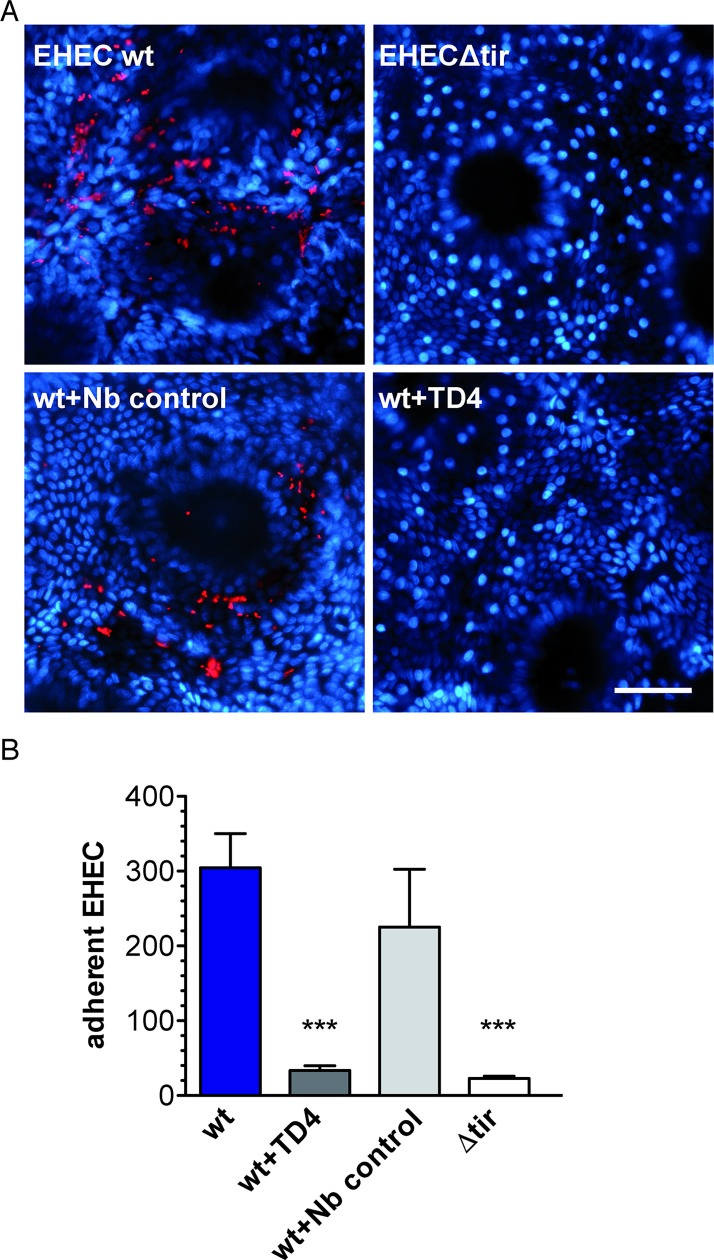
EHEC binding to human colonic biopsies is reduced in the presence of Nb TD4. **A**. Biopsy samples from the transverse colon were infected for 8 h with EHEC wild-type alone (EHEC wt) or in the presence of 200 nM Nb against TirM (wt + TD4) or amylase (wt + Nb control). Incubations with EHECΔ*tir* were included as negative control. (A) Tissue samples were stained for EHEC (red) and cell nuclei (blue), bar = 50 μm. **B**. EHEC colonisation was quantified by counting numbers of adherent bacteria in a surface area of 1 mm^2^. Data from five experiments in triplicate are expressed as means with standard errors of the means, p*** < 0.001 versus wt.

We tested the effect of TD4 on EHEC binding to human colonic mucosa by employing IVOC. Human colonic biopsy samples were infected with EHEC in the presence or absence of TD4 or control Nb (Vamy). In addition, infection with EHECΔ*tir* was included as a negative control. Immunostaining of biopsy samples showed a significant reduction in the number of adherent EHEC in the presence of TD4 but not of the control Nb (Fig 7). As expected, very few adherent bacteria were observed in biopsy samples infected with EHECΔ*tir* (Fig 7). These results demonstrate that Nb TD4 reduces EHEC binding to human colonic mucosa *ex vivo*.

## Discussion

EHEC infections are associated with severe diseases such as bloody diarrhoea and HUS [1, 2]. Efficient therapies against EHEC infections are lacking, and current treatment is based on fluid replacement and supportive care [43]. However, increasing knowledge on EHEC virulence factors and infection mechanisms is contributing to the development of new treatment strategies [44], such as inhibition of quorum sensing [45], use of EHEC LPS-specific bacteriocins [46] and inhibition of Stx binding to its host receptor globotriaosylceramide (Gb3) with antibodies [47, 48] or other ligands [49].

In this work, we tested the possibility of using specific Nbs against the EHEC proteins EspA, intimin and Tir as an alternative approach to interfere with EHEC infection. Nb clones binding Int280 (IB10) or EspA (EC7) did not interfere with EHEC infection. Since intimin covers the entire surface of EHEC, binding of Nb IB10 in the concentration used might not be sufficient to mask all the Tir-binding sites, despite some inhibitory activity of this Nb in the *in vitro* Int280:TirM binding assay. Similarly, binding of Nb EC7 to EspA, which forms the translocation filament, did not affect EHEC infection nor inhibit Tir translocation. In contrast, a Nb binding TirM (TD4) reduced the attachment of EHEC and actin pedestal formation in HeLa cells. As the TirM domain is exposed on the host cell surface after Tir translocation [13, 14], binding of TD4 appears to block intimin binding. The fact that TirM is only presented on infected cells, suggests that a relatively low Nb concentration is needed for inhibition.

Staining of Tir after EHEC infection showed that TD4 hindered the formation of actin pedestals by preventing the characteristic Tir clustering produced at the bacterial:host interface [19, 50], which is achieved even after its addition 2 h post infection and is maintained for 6 h. *In vitro* protein interaction assays confirmed a strong inhibition of Int280:TirM interaction by the presence of TD4, which prevents Tir clustering by binding to TirM. SPR analysis of this interaction demonstrated an extremely slow dissociation rate of TD4. This analysis also revealed that the affinity for TD4 to TirM (K_D_ ≤ 4 nM) is at least 10 times higher than the affinity of Int280 to TirM (K_D_ ~40 nM). Strikingly, Int280 showed a fast dissociation of TirM, suggesting a dynamic interaction.

SPR experiments also determined that the TirM epitope recognized by TD4 could partially overlap with the binding region of Int280 as the addition of TD4 reduces the binding of Int280 to TirM. We mapped two TirM consecutive non-overlapping peptides bound by TD4: VNIDELGNAIPS (296–307) and GVLKDDVVANIE (308–319). It may be possible that each of these peptides is recognized by different CDRs of the Nb TD4, but this experiment does not exclude that TD4 may recognize a conformational structure of TirM. Its CDRs could still bind to the primary structure of these peptides, albeit with reduced affinity. Importantly, these recognized peptides are fully conserved in all Tir sequences from EHEC strains. We also determined that TD4 does not bind to TirM_EPEC_ and has a weak interaction with TirM_CR_, which are highly similar but not identical to TirM_EHEC._ This information helped us to narrow the interaction site of TD4 and TirM_EHEC_ by comparing the TirM sequences of the three pathogens. Differences between TirM of EHEC and CR—i.e. V309, N317 and E319- reduce but do not abolish the interaction with TD4. On the other hand, differences with TirM of EPEC—i.e. E300, L301, V314 and A316—could be critical for the binding of TD4, likely representing energetic hotspots of protein-protein interaction [51, 52].

We could further localize the residues that may participate in the interaction of TD4 with TirM based on the crystal structure of Int280 and Tir of EPEC [15]. Within the TirM sequence, it has been identified the so-called Int280-binding domain (IBD) [13], composed of two long alpha-helices (HA, residues 271–288, and HB, residues 312–331) separated by a ß-hairpin (residues 294–308). The described complex of EPEC reveals that the Int:Tir interaction is primarily mediated by the lectin-like D3 domain of Int280 and the ß-hairpin and the N-terminal part of the HB of Tir IBD, corresponding to residues 294–313 of TirM_EHEC_. The peptides identified to which TD4 binds (residues 296–319 of TirM_EHEC_), are enclosed within the IBD of Tir, indicating that TD4 is directly interfering with the Int:Tir interaction.

Importantly, we have shown that TD4 can also block the interaction of EHEC to intestinal human colonic tissue *ex vivo* [7], as the number of bacteria bound to the epithelium was significantly reduced in the presence of this Nb. This result opens the possibility of testing TD4 protection in humans, which could be administered using a passive immunization strategy. The fact that TD4 shows inhibitory activity once EHEC infection has already begun opens also the possibility of using this Nb as a therapeutic Ab to treat infections.

Nbs can be overproduced in bacteria, yeast, plants and mammalian cells to obtain highly concentrated purified proteins [53–56]. A purified Nb recognising EHEC toxins Stx1 and Stx2 has been administered, in combination with IgG, for the treatment of HUS [57]. However, the use of purified antibodies is a costly strategy for therapy development. To circumvent this problem, some studies describe the production of Abs and Nbs in edible plants and seeds. The production of Abs in edible tissues allows oral passive immunization at the gastric mucosal surface. For instance, a Nb against rotavirus infection has been expressed in rice and shown to protect infant mice from severe diarrhoea [58]. Abs contained in seeds enable long-term storage and the direct use for passive immunization with oral administration, which is particularly advantageous. Interestingly, a Nb against enterotoxigenic *E*. *coli* (ETEC) has been fused to the constant region (Fc) of immunoglobulins and produced in seeds. Piglets fed with these seeds were protected against ETEC infection [59, 60].

Alternatively, probiotic strains such as *E*. *coli* Nissle 1917 (EcN) [61] could be used for delivery of TD4 to the gastrointestinal tract. EcN is known to compete with EHEC for colonisation of the mouse intestine [62] through specific mechanisms including the secretion of microcins [61, 63]. Hence, secretion of TD4 by EcN could enhance its natural anti-microbial activity and leads to the development of a superior therapeutic strain against EHEC infection. Other probiotic bacteria can be considered for local delivery of Nb TD4. For instance, Gram-positive *Lactobacillus* strains producing surface-bound or secreted Nbs against rotaviruses have been shown to reduce the severity and duration of rotavirus-induced diarrhoea in mice [64–66].

Overall, this study demonstrates that a Nb recognising Tir reduces intimate attachment of EHEC to human cells and colonic tissue by competing with its natural partner, intimin, thereby preventing colonization of the epithelium. These results open the possibility for passive immunization and therapeutic strategies that could prevent EHEC adhesion to intestinal tissues during infection. This could also be applied to reduce the prevalence of EHEC in its natural bovine host and minimize the risk of EHEC contamination into the food chain.

## Methods

### Ethics statement

This study was performed with approval from the University of East Anglia Faculty of Medicine and Health Ethics Committee (ref 2010/11-030). All samples were registered with the Norwich Biorepository which has approval from the National Research Ethics Service (ref 08/h0304/85+5). Biopsy samples from the transverse colon were obtained with informed written consent during colonoscopy of adult patients. All samples were anonymized.

### Bacterial culture

All *E*. *coli* strains used in this work are listed in Table 1. Bacteria were grown at 37°C on Lysogeny broth (LB) agar plates (1.5% w/v), in liquid LB or Dulbecco’s Modified Eagle’s Medium (DMEM). Ampicillin (Ap, 150 μg/ml), Chloramphenicol (Cm, 30 μg/ml) and Kanamycin (Km, 50 μg/ml) were added for plasmid selection as required. For infection of HeLa cells, EHEC strains were grown for 8 h at 37°C (200 rpm) in a flask with 10 mL of liquid LB, inoculated in capped Falcon tubes (BD Biosciences) with 5 mL DMEM, and incubated o/n at 37°C in a CO_2_ incubator (static) for the induction of the T3SS. For infection of biopsy samples, 2 ml of LB media were inoculated with an EHEC colony from an LB-agar plate and grown standing at 37 ^o^C overnight (o/n).

**Table 1:** Bacterial strains and plasmids used in this work.

Name	Relevant characteristics	Source or Reference
DH10B-T1R	(F^-^ λ^-^) *mcrA* Δ*mrr-hsdRMS-mcrBC φ80lacZ*Δ*M15*, Δ*galU galK rpsL*(Str^R^), *nupG tonA*	Invitrogen, [67]
HB2151	Δ*lac-pro*, *ara*, *nal*^R^, *thi*, F’(*proAB lacI*^*Q*^ *lacZ*ΔM15)	[68]
BL21 (DE3)	F^-^; *ompT hsdSB*(r_B_–, m_B_–) *gal dcm lon* λ(DE3[*lacI lacUV5-T7 gene1 ind1 sam7 nin5*])	Novagen-Merck, [69]
EHEC	O157:H7 EDL933 *stx1*- *stx2*-	[4]
EHECΔ*tir*	O157:H7 EDL933 *stx1*- *stx2*- Δ*tir*	[70]
pEHlyA5-TD4	Ap^R^; pUC ori, *lac* promoter, N-terminal His tag, Nb TD4, HA- and E-tags, C-HlyA	[39]
pEHlyA5-IB10	Ap^R^; pUC ori, *lac* promoter, N-terminal His tag, Nb IB10, HA- and E-tags, C-HlyA	[39]
pEHlyA5-EC7	Ap^R^; pUC ori, *lac* promoter, N-terminal His tag, Nb EC7, HA- and E-tags, C-HlyA	[39]
pEHlyA5-Vamy	Ap^R^; pUC ori, *lac* promoter, N-terminal His tag, Nb Vamy, HA- and E-tags, C-HlyA	[39]
pVDL9.3	Cm^R^; pSC101 ori, *lac* promoter, *hlyB hlyD*	[71]
pVDL9.3-TD4	pVDL9.3 derivative; operon expressing TD4-HlyA, HlyB and HlyD	This work
pVDL9.3-Vamy	pVDL9.3 derivative; operon expressing Vamy-HlyA, HlyB and HlyD	This work
pET28a	Km^R^; pBR ori, T7 promoter, N-terminal His-tagged fusions	Novagen-Merck
pET28a-TirM_EHEC_	pET28a derivative; expression of His-tagged TirM of EHEC	[39]
pET28a-Int280_EHEC_	pET28a derivative; expression of His-tagged Int280 of EHEC	[39]
pET28a-TirM_EPEC_	pET28a derivative; expression His-tagged TirM of EPEC	This work
pET28a-TirM_CR_	pET28a derivative; expression His-tagged TIrM of *C*. *rodentium*	This work

### Plasmids, DNA constructs, and oligonucleotides

Plasmids used in this study are listed in Table 1. Strain *E*. *coli* DH10B-T1R was used as a host for the cloning and propagation of plasmids. TD4-HlyA and Vamy-HlyA DNA fragments were excised with BglII from pEHLYA5-TD4 and pEHLYA5-Vamy, respectively, and cloned into the same site of pVDL9.3 [71]. TirM sequences of EPEC (aa 255–363) and CR (aa 253–360) were amplified by PCR using primers listed in Table 2, cloned after *Eco*RI-*Hind*III digestion into the same sites of pET28a plasmid backbone. The TirM constructs in this plasmid are under the T7 promoter and fused to an N-terminal His-tag for purification. PCR reactions were performed with Taq DNA polymerase (Roche, NZyTech) for standard amplifications in screenings. All DNA constructs were fully sequenced (Secugen SL, Madrid, Spain).

**Table 2:** Oligonucleotides used in this work.

Name	Sequence
BamEcoTirM-EPEC	CGCGGATCCGAATTCCAGGCGTTGGCTTTGACACCGG
XhoHindTirM-EPEC	CCGCTCGAGAAGCTTACCCGATGAAAGCTGTAATTCCTCCTG
BamEcoTirM-CR	CGCGGATCCGAATTCCAGGCGGTTGCTTTGACACCAGC
XhoHindTirM-CR	CCGCTCGAGAAGCTTTATGATGAGAGATCCAATTCCTGCCGC

### Purification of antigens

Cultures of *E*. *coli* BL21(DE3) carrying the corresponding pET28a-derivative were grown at 30°C in 500 ml of LB with Km to an optical density at 600 nm (OD600) ~0.5 and subsequently induced with 1 mM isopropyl-1-thio-β-D-galactoside (IPTG) for 2 h. Bacteria were harvested by centrifugation (10 min, 10,000 x g, 4°C), resuspended in 20 ml of 50 mM NaPO_4_ pH 7, 300 mM NaCl, DNase (0.1 mg/ml; Roche) and protease inhibitor cocktail (Roche), and lysed by passing through a French-Press at 1200 psi three times. The resultant lysate was ultracentrifuged (60 min, 40000 x g, 4°C) to obtain a cleared lysate supernatant. For purification of the His-tagged Int280_EHEC_, TirM_EHEC_, TirM_EPEC_ and TirM_CR_, lysates were passed through 2 ml of pre-equilibrated Cobalt-containing resin (TALON, Takara) in a chromatography column and washed with 20 mM HEPES pH 7.4, 200 mM NaCl. The bound His-tagged proteins were eluted by adding the same buffer complemented with 150 mM imidazole. The eluted fractions were dialyzed against HEPES-buffer (sterile filtered and degassed) and concentrated 10-fold in a 3-kDa centrifugal filter unit (Amicon Ultra-15). Proteins were loaded onto a gel filtration column (HiLoad 16/600 Superdex 75 preparative grade, GE Healthcare), pre-equilibrated with HEPES-buffer and calibrated with protein markers (Gel Filtration Standards, Bio-Rad) and Blue dextran (for exclusion volume Vo; Sigma). Fractions of 1 ml containing the purified proteins were collected and checked for purity by SDS-PAGE. Protein concentration was estimated using the Bicinchoninic acid protein assay kit (Thermo Scientific).

### Purification of Nb-HlyA fusions

Cultures of *E*. *coli* strain HB2151 carrying pVDL9.3 (*hlyB hlyD*) and the indicated pEHLYA5-derivative, or pVDL9.3-derivatives with Nb-HlyA fusions (Table 1), were grown o/n at 30°C (170 rpm) in liquid LB with the appropriate antibiotics. Next, bacteria were inoculated in fresh medium (200 ml of liquid media in 1L flask) and grown at 37°C (170 rpm) until OD600 reached 0.4. At this point, bacteria were induced with 1 mM (IPTG and further incubated for 6 h with shaking (100 rpm). The cultures were centrifuged twice (10 min, 10000 g, 4°C) to retrieve the supernatants, which were mildly sonicated (3 pulses of 5 seconds) and filtered (0.2 μm syringe filters). Then, they were loaded in columns for metal affinity chromatography (IMAC) purification. The supernatants were loaded at ca 4 ml/min onto chromatography columns with pre-equilibrated Cobalt-containing resin (TALON, Takara). Columns were washed with Tris pH 7.5 (50 mM) NaCl (150 mM) or HEPES buffer and eluted with a gradient of imidazole reaching 500 mM. A second purification step by gel filtration was performed for His-tagged antigens and Nb-HlyA fusions used in Surface Plasmon Resonance (SPR). Fractions eluted from metal-affinity chromatography were dialysed against HEPES-buffer (sterile filtered and degassed) and concentrated to 2 ml in a 3 kDa centrifugal filter unit (Amicon Ultra-15, Millipore). Next, protein samples were loaded onto a calibrated gel filtration column (HiLoad 16/600 Superdex 75, GE Healthcare), pre-equilibrated with HEPES-buffer. The elution of Nb-HlyA proteins was performed using HEPES buffer and collecting 1 ml fractions. Protein concentration was estimated using the BCA protein assay kit (Thermo Scientific).

### Synthesis and recognition of TirM_EHEC_ peptides by TD4-HlyA

For the generation of 12-mer TirM_EHEC_ peptides on a PVDF membrane, a MultiPep RSi synthesizer (Intavis) with SPOT module (Proteomics Service, CNB-CSIC) was used. The resulting membrane was blocked in PBS containing 0.1% Tween 20 (PBST) and 3% (w/v) skimmed milk for 1 h at room temperature (RT) and subsequently incubated in purified TD4-HlyA dissolved in PBST, 3% skimmed milk for 2 h. After washing in PBST, the membrane was sequentially incubated with anti-E tag mAb (Phadia, 1:5000) and secondary rabbit anti-mouse IgG-POD (1:5000, Sigma). Signal detection was performed using the Clarity Western ECL Substrate kit (Bio-Rad) and exposure to X-ray films (Agfa).

### Enzyme-linked immunosorbent assay (ELISA)

ELISA was performed as described previously [39]. Briefly, 96-well immunoplates (Maxisorp, Nunc) were coated for 2 h at RT with 5 μg/ml of purified TirM (from EHEC, EPEC or CR, as indicated) diluted in PBS. Bovine serum albumin (BSA, Roche) was used as a negative control antigen. Nb-HlyA fusions were added at the indicated concentrations for 1 h and plates were subsequently washed three times with PBS. For detection of bound Nb-HlyA fusions, anti-E-tag mAb (1:2000; Phadia) and anti-mouse IgG-POD (1:2000; Sigma), as secondary antibody, were added. The reaction was developed with o-phenylenediamine (Sigma) and H_2_O_2_ (Sigma), as previously reported [72], and the OD490 was determined using a microplate reader (iMark ELISA plate reader, Bio-Rad).

For the neutralization assay, 1 mg/ml Int280 was biotinylated using a 20-fold molar excess of Biotinamidocaproate N-hydroxysuccinimide ester (Sigma). After incubation on a gyratory wheel for 1 h at RT, the reaction was stopped by addition of 50 mM Tris-HCl pH 7.5, and placement on ice for 1 h. The reaction mix was subsequently loaded onto a pre-packed column for gel filtration chromatography (Sephadex G25 PD-10; GE Healthcare) and the biotinylated protein was eluted in 500-μl fractions with PBS. Protein concentrations were estimated using the BCA protein assay kit (Thermo Scientific). For the assay, 5 μg/ml non-biotinylated TirM was bound to plastic 96 wells plates for 2 h. The wells were blocked with 3% (w/v) skimmed milk in PBS for 1 h. At the same time, biotinylated Int280 (50 μg/ml) was incubated with a 1:50 dilution of the camel immune or preimmune serums or 1 μM (50 μg/ml) of the corresponding purified Nb-HlyA. These solutions were added to the microtiter wells for 1 h incubation after removing the blocking solution. Then, the wells were developed as a standard ELISA using Streptavidin-POD (Roche, Sigma).

### Surface Plasmon Resonance (SPR)

SPR experiments were performed using BiaCore3000 (GE Healthcare). All proteins solutions were dialyzed against HEPES-buffer (sterile filtered and degassed) at 4°C for 2 h. TirM_EHEC_ was biotinylated (as described above) at 0.1 μg/ml and immobilised on a Streptavidin SA chip (GE Healthcare) at 150 response units (RU) at a flow rate of 10 μl/min in HEPES-buffer containing 0.005% (v/v) of the surfactant Polysorbate 20 (P20, GE Healthcare). To determine binding kinetics, dilutions of purified TD4-HlyA or Int280 (as indicated) were run at 30 μl/min in HEPES-buffer and sensograms were generated. Regeneration of TD4-HlyA was performed by sequential injections of 10 μl 10 mM glycine-HCl pH 1.7, 5 μl 5 mM NaOH and 10 μl 10 mM glycine-HCl pH 1.7. No regeneration was needed for Int280. Sensograms with different concentrations of analyte were overlaid, aligned and analysed with BIAevaluation 4.1 software (GE Healthcare) under assumption of the 1:1 Langmuir model and using both the simultaneous kinetics model and the steady-state equilibrium analysis [73].

### EHEC infection of HeLa cells

The human cervix carcinoma cell line HeLa (ATCC, CCL-2) was grown in DMEM supplemented with 10% fetal bovine serum and 2 mM glutamine at 37°C in a 5% CO_2_ atmosphere. For infection, cells were seeded out on glass coverslips in 24-well plates at a concentration of 10^5^ cells/well. Cells were inoculated with EHEC at a multiplicity of infection (MOI) of 1000 for 3 h at 37°C in a 5% CO_2_ atmosphere. The purified Nb-HlyA fusions, at the indicated concentrations, were added to the cells simultaneously with EHEC bacteria, or 1 h or 2 h post-infection, as indicated, in a final volume of 0.5 or 1 ml. The infection was stopped by three washes with sterile PBS. In the case of EHEC infections for 6 h, cells were washed with PBS after 3 h of infection, fresh medium and Nbs were then added, and incubation was continued for another 3 h.

### Immunofluorescence microscopy

Cells were fixed with 4% (w/v) paraformaldehyde in PBS for 20 min at RT and permeabilized in 0.1% (v/v) of saponin (Sigma) in PBS for 10 min. All antibodies were diluted in PBS with 10% goat serum (Sigma), and mouse monoclonal anti-O157 (Abcam, 1:500), mouse monoclonal anti-HA (Cambridge bioscience, 1:200) and rabbit polyclonal anti-Tir_EHEC_ (1:200) were used to detect EHEC bacteria, HA-tag and Tir_EHEC_, respectively. After incubation for 1 h at RT, coverslips were washed three times with PBS, and incubated for 45 min with secondary antibodies, Alexa477-conjugated goat anti-mouse IgG or Alexa647-conjugated goat anti-rabbit-IgG (1:500, ThermoFisher Scientific), Tetramethylrhodamine (TRITC)-conjugated phalloidin (1:500, Sigma) and 4',6-Diamidino-2-phenylindole (DAPI) (1:500, Sigma) to label F-actin and DNA, respectively. Coverslips were washed 3 times with PBS after incubation, mounted in of ProLong Gold anti-fade reagent (ThermoFisher Scientific), and analysed with an SP5 confocal microscope (Leica).

### *In vitro* organ culture (IVOC) of human colonic biopsies

Biopsy samples from the transverse colon were taken from macroscopically normal areas, transported to the laboratory in IVOC medium and processed within the next hour. IVOC was performed as described previously [74]. Briefly, biopsies were mounted on foam supports in 12 well plates and incubated with 30 μl EHEC standing overnight culture (approximately 10^7^ bacteria) and 200 nM of TD4 or Nb control (Vamy). Samples were incubated for 8 h on a rocking platform at 37°C in a 5% CO_2_ atmosphere with medium changes after 4 and 6 h of incubation. At the end of the experiment, tissues were washed in PBS to remove the mucus layer and fixed in 3.7% formaldehyde/PBS for 20 min at RT. Samples were permeabilised with 0.1% Triton X-100/PBS, and blocked with 0.5% BSA/PBS for 20 min. Tissues were incubated with goat polyclonal anti-*E*. *coli* (1:400, Abcam) for one hour, followed by incubation in Alexa Fluor 568-conjugated donkey anti-goat IgG (1:400,ThermoFisher Scientific) and DAPI for 30 min to counterstain cell nuclei. Biopsy samples were mounted with Vectashield mounting medium (Vector Labs) and analysed using an Axio Imager M2 motorized fluorescence microscope (Zeiss). EHEC colonisation of colonic biopsies was quantified by counting adherent bacteria in a surface area of 1 mm^2^.

### Statistics

Means and standard errors of experimental values were calculated using Prism 5.0 (GraphPad software Inc). Statistical analyses comparing multiple groups were performed using one-way ANOVA and Dunnett’s post- test. Statistics for Fig 2 were done using One Way ANOVA analysis doing logarithms for normal distribution. Data was corrected with the Bonferroni test. A value of p<0.05 was considered significant.

## Supporting information

S1 Data Taxonomy report obtained after BLASTP search of non-redundant databases with TirM peptide "VNIDELGNAIPSGVLKDDVVANIE" (residues 296–319) from EHEC O157:H7 strain EDL933. The generated HTML file shows full conservation of this TirM peptide among EHEC strains (score = 77.4).
(HTML)
