# Supplementary material for: A nanobody targeting the translocated intimin receptor inhibits the attachment of enterohemorrhagic E. coli to human colonic mucosa
Source: PLoS Pathog. 2019 Aug 29;15(8):e1008031. doi: 10.1371/journal.ppat.1008031 (PMC6738647; doi:10.1371/journal.ppat.1008031)
Supplement: S1 Data — The generated HTML file shows full conservation of this TirM peptide among EHEC strains (score = 77.4). (HTML) [file ppat.1008031.s001.html]

NCBI Blast:Protein Sequence


- NCBI Home
- Sign in to NCBI
- Skip to Main Content
- Skip to Navigation
- About NCBI Accesskeys

U.S. National Library of Medicine

NCBI
National Center for Biotechnology Information

- My NCBI
- Sign in to NCBI
- Register
- Sign Out

BLAST ® » blastp suite » RID-K3M7T9RP014


- Home
- Recent Results
- Saved Strategies
- Help

Tax BLAST report


- Your search parameters were adjusted to search for a short input sequence.

Taxonomy report description


# Tax BLAST report

Results for:

lcl|Query\_129683 None(24aa)
[?]


Your BLAST job specified more than one input sequence.
This box lets you choose which input sequence to show BLAST results for.

RID
:   K3M7T9RP014 (Expires on 07-20 22:17 pm)

Query ID
:   lcl|Query\_129683
:   lcl|Query\_129683

Description
:   None

Molecule type
:   amino acid

Query Length
:   24

Database Name
:   nr

Description
:   All non-redundant GenBank CDS translations+PDB+SwissProt+PIR+PRF excluding environmental samples from WGS projects See details

Program
:   BLASTP 2.9.0+ Citation


Reference 

Stephen F. Altschul, Thomas L. Madden, Alejandro A. Schäffer, Jinghui Zhang, Zheng Zhang, Webb Miller, and David J. Lipman (1997), "Gapped BLAST and PSI-BLAST: a new generation of protein database search programs", Nucleic Acids Res. 25:3389-3402.

## Lineage Report

Organism Report
Taxonomy Report

Lineage Report

| Organism | Blast Name | Score | Number of Hits | Description |
| --- | --- | --- | --- | --- |
| root |  |  | 1288 |  |
| .Enterobacteriaceae | enterobacteria |  | 1286 |  |
| ..Escherichia | enterobacteria |  | 1281 |  |
| ...Escherichia coli | enterobacteria |  | 871 |  |
| ....Escherichia coli O157:H7 | enterobacteria |  | 37 |  |
| .....Escherichia coli O157:H7 str. EDL933 | enterobacteria | 77.4 | 6 | Escherichia coli O157:H7 str. EDL933 hits |
| .....Escherichia coli O157:H7 str. EC869 | enterobacteria | 77.4 | 1 | Escherichia coli O157:H7 str. EC869 hits |
| .....Escherichia coli O157:H7 str. 2009C-4258 | enterobacteria | 77.4 | 1 | Escherichia coli O157:H7 str. 2009C-4258 hits |
| .....Escherichia coli O157:H7 str. F7350 | enterobacteria | 77.4 | 1 | Escherichia coli O157:H7 str. F7350 hits |
| .....Escherichia coli O157:H7 str. F7377 | enterobacteria | 77.4 | 1 | Escherichia coli O157:H7 str. F7377 hits |
| .....Escherichia coli O157:H7 str. Sakai | enterobacteria | 77.4 | 2 | Escherichia coli O157:H7 str. Sakai hits |
| .....Escherichia coli O157:H7 str. EC4115 | enterobacteria | 77.4 | 2 | Escherichia coli O157:H7 str. EC4115 hits |
| .....Escherichia coli O157:H7 str. TW14359 | enterobacteria | 77.4 | 2 | Escherichia coli O157:H7 str. TW14359 hits |
| .....Escherichia coli O157:H7 str. SS17 | enterobacteria | 77.4 | 1 | Escherichia coli O157:H7 str. SS17 hits |
| .....Escherichia coli O157:H7 str. SS52 | enterobacteria | 77.4 | 1 | Escherichia coli O157:H7 str. SS52 hits |
| .....Escherichia coli O157:H7 str. EC4196 | enterobacteria | 77.4 | 1 | Escherichia coli O157:H7 str. EC4196 hits |
| .....Escherichia coli O157:H7 str. EC4113 | enterobacteria | 77.4 | 1 | Escherichia coli O157:H7 str. EC4113 hits |
| .....Escherichia coli O157:H7 str. EC4076 | enterobacteria | 77.4 | 1 | Escherichia coli O157:H7 str. EC4076 hits |
| .....Escherichia coli O157:H7 str. EC4401 | enterobacteria | 77.4 | 1 | Escherichia coli O157:H7 str. EC4401 hits |
| .....Escherichia coli O157:H7 str. EC4486 | enterobacteria | 77.4 | 1 | Escherichia coli O157:H7 str. EC4486 hits |
| .....Escherichia coli O157:H7 str. EC4501 | enterobacteria | 77.4 | 1 | Escherichia coli O157:H7 str. EC4501 hits |
| .....Escherichia coli O157:H7 str. EC508 | enterobacteria | 77.4 | 1 | Escherichia coli O157:H7 str. EC508 hits |
| .....Escherichia coli O157:H7 str. EC4206 | enterobacteria | 77.4 | 1 | Escherichia coli O157:H7 str. EC4206 hits |
| .....Escherichia coli O157:H7 str. EC4045 | enterobacteria | 77.4 | 1 | Escherichia coli O157:H7 str. EC4045 hits |
| .....Escherichia coli O157:H7 str. EC4042 | enterobacteria | 77.4 | 1 | Escherichia coli O157:H7 str. EC4042 hits |
| .....Escherichia coli O157:H7 str. TW14588 | enterobacteria | 77.4 | 1 | Escherichia coli O157:H7 str. TW14588 hits |
| .....Escherichia coli O157:H7 str. EC1212 | enterobacteria | 77.4 | 1 | Escherichia coli O157:H7 str. EC1212 hits |
| .....Escherichia coli O157:H7 str. G5101 | enterobacteria | 77.4 | 1 | Escherichia coli O157:H7 str. G5101 hits |
| .....Escherichia coli O157:H7 str. LSU-61 | enterobacteria | 77.4 | 1 | Escherichia coli O157:H7 str. LSU-61 hits |
| .....Escherichia coli O157:H7 str. 1044 | enterobacteria | 77.4 | 1 | Escherichia coli O157:H7 str. 1044 hits |
| .....Escherichia coli O157:H7 str. 1125 | enterobacteria | 77.4 | 1 | Escherichia coli O157:H7 str. 1125 hits |
| .....Escherichia coli O157:H7 str. TW14313 | enterobacteria | 77.4 | 2 | Escherichia coli O157:H7 str. TW14313 hits |
| .....Escherichia coli O157:H7 str. 2009EL2109 | enterobacteria | 77.4 | 1 | Escherichia coli O157:H7 str. 2009EL2109 hits |
| .....Escherichia coli O157:H7 str. 2009EL1705 | enterobacteria | 77.4 | 1 | Escherichia coli O157:H7 str. 2009EL1705 hits |
| .....Escherichia coli O157:H7 str. K5806 | enterobacteria | 77.4 | 1 | Escherichia coli O157:H7 str. K5806 hits |
| .....Escherichia coli O157:H7 str. 2011EL-2312 | enterobacteria | 77.4 | 1 | Escherichia coli O157:H7 str. 2011EL-2312 hits |
| .....Escherichia coli O157:H7 str. 2011EL-2289 | enterobacteria | 77.4 | 1 | Escherichia coli O157:H7 str. 2011EL-2289 hits |
| .....Escherichia coli O157:H7 str. 2011EL-2288 | enterobacteria | 77.4 | 1 | Escherichia coli O157:H7 str. 2011EL-2288 hits |
| .....Escherichia coli O157:H7 str. 2011EL-2114 | enterobacteria | 77.4 | 1 | Escherichia coli O157:H7 str. 2011EL-2114 hits |
| .....Escherichia coli O157:H7 str. 2011EL-2287 | enterobacteria | 77.4 | 1 | Escherichia coli O157:H7 str. 2011EL-2287 hits |
| .....Escherichia coli O157:H7 str. 2011EL-2286 | enterobacteria | 77.4 | 1 | Escherichia coli O157:H7 str. 2011EL-2286 hits |
| .....Escherichia coli O157:H7 str. 2011EL-2113 | enterobacteria | 77.4 | 1 | Escherichia coli O157:H7 str. 2011EL-2113 hits |
| .....Escherichia coli O157:H7 str. 2011EL-2112 | enterobacteria | 77.4 | 1 | Escherichia coli O157:H7 str. 2011EL-2112 hits |
| .....Escherichia coli O157:H7 str. 2011EL-2111 | enterobacteria | 77.4 | 1 | Escherichia coli O157:H7 str. 2011EL-2111 hits |
| .....Escherichia coli O157:H7 str. 2011EL-2108 | enterobacteria | 77.4 | 1 | Escherichia coli O157:H7 str. 2011EL-2108 hits |
| .....Escherichia coli O157:H7 str. 2011EL-2109 | enterobacteria | 77.4 | 1 | Escherichia coli O157:H7 str. 2011EL-2109 hits |
| .....Escherichia coli O157:H7 str. 2011EL-2107 | enterobacteria | 77.4 | 1 | Escherichia coli O157:H7 str. 2011EL-2107 hits |
| .....Escherichia coli O157:H7 str. 2011EL-2106 | enterobacteria | 77.4 | 1 | Escherichia coli O157:H7 str. 2011EL-2106 hits |
| .....Escherichia coli O157:H7 str. 2011EL-2105 | enterobacteria | 77.4 | 1 | Escherichia coli O157:H7 str. 2011EL-2105 hits |
| .....Escherichia coli O157:H7 str. 2011EL-2104 | enterobacteria | 77.4 | 1 | Escherichia coli O157:H7 str. 2011EL-2104 hits |
| .....Escherichia coli O157:H7 str. 2011EL-2103 | enterobacteria | 77.4 | 1 | Escherichia coli O157:H7 str. 2011EL-2103 hits |
| .....Escherichia coli O157:H7 str. 2011EL-2101 | enterobacteria | 77.4 | 1 | Escherichia coli O157:H7 str. 2011EL-2101 hits |
| .....Escherichia coli O157:H7 str. 2011EL-2099 | enterobacteria | 77.4 | 1 | Escherichia coli O157:H7 str. 2011EL-2099 hits |
| .....Escherichia coli O157:H7 str. 08-4169 | enterobacteria | 77.4 | 1 | Escherichia coli O157:H7 str. 08-4169 hits |
| .....Escherichia coli O157:H7 str. 08-3037 | enterobacteria | 77.4 | 1 | Escherichia coli O157:H7 str. 08-3037 hits |
| .....Escherichia coli O157:H7 str. 08-3527 | enterobacteria | 77.4 | 1 | Escherichia coli O157:H7 str. 08-3527 hits |
| .....Escherichia coli O157:H7 str. 2011EL-2098 | enterobacteria | 77.4 | 1 | Escherichia coli O157:H7 str. 2011EL-2098 hits |
| .....Escherichia coli O157:H7 str. 2011EL-2097 | enterobacteria | 77.4 | 1 | Escherichia coli O157:H7 str. 2011EL-2097 hits |
| .....Escherichia coli O157:H7 str. 2011EL-2096 | enterobacteria | 77.4 | 1 | Escherichia coli O157:H7 str. 2011EL-2096 hits |
| .....Escherichia coli O157:H7 str. 2011EL-2094 | enterobacteria | 77.4 | 1 | Escherichia coli O157:H7 str. 2011EL-2094 hits |
| .....Escherichia coli O157:H7 str. 2011EL-2093 | enterobacteria | 77.4 | 1 | Escherichia coli O157:H7 str. 2011EL-2093 hits |
| .....Escherichia coli O157:H7 str. 2011EL-2092 | enterobacteria | 77.4 | 1 | Escherichia coli O157:H7 str. 2011EL-2092 hits |
| .....Escherichia coli O157:H7 str. 2011EL-2091 | enterobacteria | 77.4 | 1 | Escherichia coli O157:H7 str. 2011EL-2091 hits |
| .....Escherichia coli O157:H7 str. 2011EL-2090 | enterobacteria | 77.4 | 1 | Escherichia coli O157:H7 str. 2011EL-2090 hits |
| .....Escherichia coli O157:H7 str. 2011EL-1107 | enterobacteria | 77.4 | 1 | Escherichia coli O157:H7 str. 2011EL-1107 hits |
| .....Escherichia coli O157:H7 str. 2010C-4979C1 | enterobacteria | 77.4 | 1 | Escherichia coli O157:H7 str. 2010C-4979C1 hits |
| .....Escherichia coli O157:H7 str. 07-3091 | enterobacteria | 77.4 | 1 | Escherichia coli O157:H7 str. 07-3091 hits |
| .....Escherichia coli O157:H7 str. 06-4039 | enterobacteria | 77.4 | 1 | Escherichia coli O157:H7 str. 06-4039 hits |
| .....Escherichia coli O157:H7 str. 07-3391 | enterobacteria | 77.4 | 1 | Escherichia coli O157:H7 str. 07-3391 hits |
| .....Escherichia coli O157:H7 str. 06-3745 | enterobacteria | 77.4 | 1 | Escherichia coli O157:H7 str. 06-3745 hits |
| .....Escherichia coli O157:H7 str. F6142 | enterobacteria | 77.4 | 1 | Escherichia coli O157:H7 str. F6142 hits |
| .....Escherichia coli O157:H7 str. F6750 | enterobacteria | 77.4 | 1 | Escherichia coli O157:H7 str. F6750 hits |
| .....Escherichia coli O157:H7 str. F6749 | enterobacteria | 77.4 | 1 | Escherichia coli O157:H7 str. F6749 hits |
| .....Escherichia coli O157:H7 str. F6751 | enterobacteria | 77.4 | 1 | Escherichia coli O157:H7 str. F6751 hits |
| .....Escherichia coli O157:H7 str. F7384 | enterobacteria | 77.4 | 1 | Escherichia coli O157:H7 str. F7384 hits |
| .....Escherichia coli O157:H7 str. F7410 | enterobacteria | 77.4 | 1 | Escherichia coli O157:H7 str. F7410 hits |
| .....Escherichia coli O157:H7 str. G5303 | enterobacteria | 77.4 | 1 | Escherichia coli O157:H7 str. G5303 hits |
| .....Escherichia coli O157:H7 str. H2495 | enterobacteria | 77.4 | 1 | Escherichia coli O157:H7 str. H2495 hits |
| .....Escherichia coli O157:H7 str. K1420 | enterobacteria | 77.4 | 1 | Escherichia coli O157:H7 str. K1420 hits |
| .....Escherichia coli O157:H7 str. H2498 | enterobacteria | 77.4 | 1 | Escherichia coli O157:H7 str. H2498 hits |
| .....Escherichia coli O157:H7 str. K1792 | enterobacteria | 77.4 | 1 | Escherichia coli O157:H7 str. K1792 hits |
| .....Escherichia coli O157:H7 str. K1845 | enterobacteria | 77.4 | 1 | Escherichia coli O157:H7 str. K1845 hits |
| .....Escherichia coli O157:H7 str. K1796 | enterobacteria | 77.4 | 1 | Escherichia coli O157:H7 str. K1796 hits |
| .....Escherichia coli O157:H7 str. K1921 | enterobacteria | 77.4 | 1 | Escherichia coli O157:H7 str. K1921 hits |
| .....Escherichia coli O157:H7 str. K2188 | enterobacteria | 77.4 | 1 | Escherichia coli O157:H7 str. K2188 hits |
| .....Escherichia coli O157:H7 str. K2191 | enterobacteria | 77.4 | 1 | Escherichia coli O157:H7 str. K2191 hits |
| .....Escherichia coli O157:H7 str. K2192 | enterobacteria | 77.4 | 1 | Escherichia coli O157:H7 str. K2192 hits |
| .....Escherichia coli O157:H7 str. K2581 | enterobacteria | 77.4 | 1 | Escherichia coli O157:H7 str. K2581 hits |
| .....Escherichia coli O157:H7 str. K2622 | enterobacteria | 77.4 | 1 | Escherichia coli O157:H7 str. K2622 hits |
| .....Escherichia coli O157:H7 str. K2845 | enterobacteria | 77.4 | 1 | Escherichia coli O157:H7 str. K2845 hits |
| .....Escherichia coli O157:H7 str. K2854 | enterobacteria | 77.4 | 1 | Escherichia coli O157:H7 str. K2854 hits |
| .....Escherichia coli O157:H7 str. K4406 | enterobacteria | 77.4 | 1 | Escherichia coli O157:H7 str. K4406 hits |
| .....Escherichia coli O157:H7 str. K4396 | enterobacteria | 77.4 | 1 | Escherichia coli O157:H7 str. K4396 hits |
| .....Escherichia coli O157:H7 str. K4405 | enterobacteria | 77.4 | 1 | Escherichia coli O157:H7 str. K4405 hits |
| .....Escherichia coli O157:H7 str. K4527 | enterobacteria | 77.4 | 1 | Escherichia coli O157:H7 str. K4527 hits |
| .....Escherichia coli O157:H7 str. K5418 | enterobacteria | 77.4 | 1 | Escherichia coli O157:H7 str. K5418 hits |
| .....Escherichia coli O157:H7 str. K5448 | enterobacteria | 77.4 | 1 | Escherichia coli O157:H7 str. K5448 hits |
| .....Escherichia coli O157:H7 str. K5453 | enterobacteria | 77.4 | 1 | Escherichia coli O157:H7 str. K5453 hits |
| .....Escherichia coli O157:H7 str. K5449 | enterobacteria | 77.4 | 1 | Escherichia coli O157:H7 str. K5449 hits |
| .....Escherichia coli O157:H7 str. K5460 | enterobacteria | 77.4 | 1 | Escherichia coli O157:H7 str. K5460 hits |
| .....Escherichia coli O157:H7 str. K5467 | enterobacteria | 77.4 | 1 | Escherichia coli O157:H7 str. K5467 hits |
| .....Escherichia coli O157:H7 str. K5602 | enterobacteria | 77.4 | 1 | Escherichia coli O157:H7 str. K5602 hits |
| .....Escherichia coli O157:H7 str. K5609 | enterobacteria | 77.4 | 1 | Escherichia coli O157:H7 str. K5609 hits |
| .....Escherichia coli O157:H7 str. K5607 | enterobacteria | 77.4 | 1 | Escherichia coli O157:H7 str. K5607 hits |
| .....Escherichia coli O157:H7 str. K5852 | enterobacteria | 77.4 | 1 | Escherichia coli O157:H7 str. K5852 hits |
| .....Escherichia coli O157:H7 str. K6590 | enterobacteria | 77.4 | 1 | Escherichia coli O157:H7 str. K6590 hits |
| .....Escherichia coli O157:H7 str. K6676 | enterobacteria | 77.4 | 1 | Escherichia coli O157:H7 str. K6676 hits |
| .....Escherichia coli O157:H7 str. K6687 | enterobacteria | 77.4 | 1 | Escherichia coli O157:H7 str. K6687 hits |
| .....Escherichia coli O157:H7 str. K7140 | enterobacteria | 77.4 | 1 | Escherichia coli O157:H7 str. K7140 hits |
| .....Escherichia coli O157:H7 str. 08-4529 | enterobacteria | 77.4 | 1 | Escherichia coli O157:H7 str. 08-4529 hits |
| .....Escherichia coli O157:H7 str. 2009EL1913 | enterobacteria | 77.4 | 1 | Escherichia coli O157:H7 str. 2009EL1913 hits |
| .....Escherichia coli O157:H7 str. 2011EL-2313 | enterobacteria | 77.4 | 1 | Escherichia coli O157:H7 str. 2011EL-2313 hits |
| .....Escherichia coli O157:H7 str. 2011EL-2290 | enterobacteria | 77.4 | 1 | Escherichia coli O157:H7 str. 2011EL-2290 hits |
| .....Escherichia coli O157:H7 str. EC1825 | enterobacteria | 77.4 | 1 | Escherichia coli O157:H7 str. EC1825 hits |
| .....Escherichia coli O157:H7 str. K1927 | enterobacteria | 77.4 | 1 | Escherichia coli O157:H7 str. K1927 hits |
| .....Escherichia coli O157:H7 str. 2009EL1449 | enterobacteria | 77.4 | 1 | Escherichia coli O157:H7 str. 2009EL1449 hits |
| .....Escherichia coli O157:H7 str. K2324 | enterobacteria | 77.4 | 1 | Escherichia coli O157:H7 str. K2324 hits |
| ....Escherichia coli O157:H7 | enterobacteria | 77.4 | 37 | Escherichia coli O157:H7 hits |
| ....Escherichia coli 6-175-07\_S1\_C1 | enterobacteria | 77.4 | 1 | Escherichia coli 6-175-07\_S1\_C1 hits |
| ....Escherichia coli T1282\_01 | enterobacteria | 77.4 | 1 | Escherichia coli T1282\_01 hits |
| ....Escherichia coli 99.1775 | enterobacteria | 77.4 | 1 | Escherichia coli 99.1775 hits |
| ....Escherichia coli 99.1781 | enterobacteria | 77.4 | 1 | Escherichia coli 99.1781 hits |
| ....Escherichia coli 96.0939 | enterobacteria | 77.4 | 1 | Escherichia coli 96.0939 hits |
| ....Escherichia coli FRIK1996 | enterobacteria | 77.4 | 1 | Escherichia coli FRIK1996 hits |
| ....Escherichia coli FRIK1990 | enterobacteria | 77.4 | 1 | Escherichia coli FRIK1990 hits |
| ....Escherichia coli PA40 | enterobacteria | 77.4 | 1 | Escherichia coli PA40 hits |
| ....Escherichia coli PA41 | enterobacteria | 77.4 | 1 | Escherichia coli PA41 hits |
| ....Escherichia coli TW09195 | enterobacteria | 77.4 | 1 | Escherichia coli TW09195 hits |
| ....Escherichia coli FRIK920 | enterobacteria | 77.4 | 1 | Escherichia coli FRIK920 hits |
| ....Escherichia coli FRIK1999 | enterobacteria | 77.4 | 1 | Escherichia coli FRIK1999 hits |
| ....Escherichia coli NE1487 | enterobacteria | 77.4 | 1 | Escherichia coli NE1487 hits |
| ....Escherichia coli FRIK2001 | enterobacteria | 77.4 | 1 | Escherichia coli FRIK2001 hits |
| ....Escherichia coli MA6 | enterobacteria | 77.4 | 1 | Escherichia coli MA6 hits |
| ....Escherichia coli 90.0039 | enterobacteria | 77.4 | 1 | Escherichia coli 90.0039 hits |
| ....Escherichia coli 95.0183 | enterobacteria | 77.4 | 1 | Escherichia coli 95.0183 hits |
| ....Escherichia coli 99.0672 | enterobacteria | 77.4 | 1 | Escherichia coli 99.0672 hits |
| ....Escherichia coli ATCC 700728 | enterobacteria | 77.4 | 1 | Escherichia coli ATCC 700728 hits |
| ....Escherichia coli 08BKT055439 | enterobacteria | 77.4 | 1 | Escherichia coli 08BKT055439 hits |
| ....Escherichia coli T234\_00 | enterobacteria | 77.4 | 1 | Escherichia coli T234\_00 hits |
| ....Escherichia coli 14A | enterobacteria | 77.4 | 1 | Escherichia coli 14A hits |
| ....Escherichia coli 08BKT77219 | enterobacteria | 77.4 | 1 | Escherichia coli 08BKT77219 hits |
| ....Escherichia coli O55:H7 str. RM12579 | enterobacteria | 77.4 | 1 | Escherichia coli O55:H7 str. RM12579 hits |
| ....Escherichia coli DEC5C | enterobacteria | 77.4 | 1 | Escherichia coli DEC5C hits |
| ....Escherichia coli DEC5D | enterobacteria | 77.4 | 1 | Escherichia coli DEC5D hits |
| ....Escherichia coli O55:H7 str. TB182A | enterobacteria | 77.4 | 1 | Escherichia coli O55:H7 str. TB182A hits |
| ....Escherichia coli 99.0814 | enterobacteria | 77.4 | 1 | Escherichia coli 99.0814 hits |
| ....Escherichia coli 99.0815 | enterobacteria | 77.4 | 1 | Escherichia coli 99.0815 hits |
| ....Escherichia coli 99.0816 | enterobacteria | 77.4 | 1 | Escherichia coli 99.0816 hits |
| ....Escherichia coli 95.0943 | enterobacteria | 77.4 | 1 | Escherichia coli 95.0943 hits |
| ....Escherichia coli 99.1753 | enterobacteria | 77.4 | 1 | Escherichia coli 99.1753 hits |
| ....Escherichia coli B107 | enterobacteria | 77.4 | 1 | Escherichia coli B107 hits |
| ....Escherichia coli B102 | enterobacteria | 77.4 | 1 | Escherichia coli B102 hits |
| ....Escherichia coli B26-1 | enterobacteria | 77.4 | 1 | Escherichia coli B26-1 hits |
| ....Escherichia coli B26-2 | enterobacteria | 77.4 | 1 | Escherichia coli B26-2 hits |
| ....Escherichia coli B103 | enterobacteria | 77.4 | 1 | Escherichia coli B103 hits |
| ....Escherichia coli B104 | enterobacteria | 77.4 | 1 | Escherichia coli B104 hits |
| ....Escherichia coli B105 | enterobacteria | 77.4 | 1 | Escherichia coli B105 hits |
| ....Escherichia coli B106 | enterobacteria | 77.4 | 1 | Escherichia coli B106 hits |
| ....Escherichia coli B40-2 | enterobacteria | 77.4 | 1 | Escherichia coli B40-2 hits |
| ....Escherichia coli B40-1 | enterobacteria | 77.4 | 1 | Escherichia coli B40-1 hits |
| ....Escherichia coli B49-2 | enterobacteria | 77.4 | 1 | Escherichia coli B49-2 hits |
| ....Escherichia coli B83 | enterobacteria | 77.4 | 1 | Escherichia coli B83 hits |
| ....Escherichia coli B84 | enterobacteria | 77.4 | 1 | Escherichia coli B84 hits |
| ....Escherichia coli B85 | enterobacteria | 77.4 | 1 | Escherichia coli B85 hits |
| ....Escherichia coli B86 | enterobacteria | 77.4 | 1 | Escherichia coli B86 hits |
| ....Escherichia coli O145:NM str. 2010C-4557C2 | enterobacteria | 77.4 | 1 | Escherichia coli O145:NM str. 2010C-4557C2 hits |
| ....Escherichia coli O145:H28 str. RM13514 | enterobacteria | 77.4 | 1 | Escherichia coli O145:H28 str. RM13514 hits |
| ....Escherichia coli O145:H28 str. RM13516 | enterobacteria | 77.4 | 1 | Escherichia coli O145:H28 str. RM13516 hits |
| ....Escherichia coli O145:H28 str. RM12761 | enterobacteria | 77.4 | 1 | Escherichia coli O145:H28 str. RM12761 hits |
| ....Escherichia coli O145:H28 str. RM12581 | enterobacteria | 77.4 | 1 | Escherichia coli O145:H28 str. RM12581 hits |
| ....Escherichia coli O145 str. RM9872 | enterobacteria | 77.4 | 1 | Escherichia coli O145 str. RM9872 hits |
| ....Escherichia coli O145 | enterobacteria | 77.4 | 1 | Escherichia coli O145 hits |
| ....Escherichia coli O145:NM str. 2010C-3526 | enterobacteria | 77.4 | 1 | Escherichia coli O145:NM str. 2010C-3526 hits |
| ....Escherichia coli O145:NM str. 2010C-3521 | enterobacteria | 77.4 | 1 | Escherichia coli O145:NM str. 2010C-3521 hits |
| ....Escherichia coli O145:NM str. 2010C-3517 | enterobacteria | 77.4 | 1 | Escherichia coli O145:NM str. 2010C-3517 hits |
| ....Escherichia coli O145:NM str. 2010C-3518 | enterobacteria | 77.4 | 1 | Escherichia coli O145:NM str. 2010C-3518 hits |
| ....Escherichia coli O145:NM str. 2010C-3516 | enterobacteria | 77.4 | 1 | Escherichia coli O145:NM str. 2010C-3516 hits |
| ....Escherichia coli O145:NM str. 2010C-3510 | enterobacteria | 77.4 | 1 | Escherichia coli O145:NM str. 2010C-3510 hits |
| ....Escherichia coli O145:NM str. 2010C-3509 | enterobacteria | 77.4 | 1 | Escherichia coli O145:NM str. 2010C-3509 hits |
| ....Escherichia coli O145:NM str. 2010C-3511 | enterobacteria | 77.4 | 1 | Escherichia coli O145:NM str. 2010C-3511 hits |
| ....Escherichia coli O145:NM str. 2010C-3507 | enterobacteria | 77.4 | 1 | Escherichia coli O145:NM str. 2010C-3507 hits |
| ....Escherichia coli O145:NM str. 08-4270 | enterobacteria | 77.4 | 1 | Escherichia coli O145:NM str. 08-4270 hits |
| ....Escherichia coli O145:NM str. 06-3484 | enterobacteria | 77.4 | 1 | Escherichia coli O145:NM str. 06-3484 hits |
| ....Escherichia coli O145:NM str. 2010C-3508 | enterobacteria | 77.4 | 1 | Escherichia coli O145:NM str. 2010C-3508 hits |
| ....Escherichia coli O145:H28 str. 4865/96 | enterobacteria | 77.4 | 1 | Escherichia coli O145:H28 str. 4865/96 hits |
| ....Escherichia coli O145:NM | enterobacteria | 77.4 | 1 | Escherichia coli O145:NM hits |
| ....Escherichia coli O157:H- str. 493-89 | enterobacteria | 77.4 | 1 | Escherichia coli O157:H- str. 493-89 hits |
| ....Escherichia coli O157:H- str. H 2687 | enterobacteria | 77.4 | 1 | Escherichia coli O157:H- str. H 2687 hits |
| ....Escherichia coli DEC3F | enterobacteria | 77.4 | 1 | Escherichia coli DEC3F hits |
| ....Escherichia coli TW06591 | enterobacteria | 77.4 | 1 | Escherichia coli TW06591 hits |
| ....Escherichia coli 5412 | enterobacteria | 77.4 | 1 | Escherichia coli 5412 hits |
| ....Escherichia coli TW10119 | enterobacteria | 77.4 | 1 | Escherichia coli TW10119 hits |
| ....Escherichia coli 90.0091 | enterobacteria | 77.4 | 1 | Escherichia coli 90.0091 hits |
| ....Escherichia coli 99.1805 | enterobacteria | 77.4 | 1 | Escherichia coli 99.1805 hits |
| ....Escherichia coli O157:NM str. 08-4540 | enterobacteria | 77.4 | 1 | Escherichia coli O157:NM str. 08-4540 hits |
| ....Escherichia coli Xuzhou21 | enterobacteria | 77.4 | 1 | Escherichia coli Xuzhou21 hits |
| ....Escherichia coli DEC3A | enterobacteria | 77.4 | 1 | Escherichia coli DEC3A hits |
| ....Escherichia coli DEC3B | enterobacteria | 77.4 | 1 | Escherichia coli DEC3B hits |
| ....Escherichia coli DEC3C | enterobacteria | 77.4 | 1 | Escherichia coli DEC3C hits |
| ....Escherichia coli DEC4E | enterobacteria | 77.4 | 1 | Escherichia coli DEC4E hits |
| ....Escherichia coli DEC4F | enterobacteria | 77.4 | 1 | Escherichia coli DEC4F hits |
| ....Escherichia coli FDA505 | enterobacteria | 77.4 | 1 | Escherichia coli FDA505 hits |
| ....Escherichia coli FDA517 | enterobacteria | 77.4 | 1 | Escherichia coli FDA517 hits |
| ....Escherichia coli FRIK1985 | enterobacteria | 77.4 | 1 | Escherichia coli FRIK1985 hits |
| ....Escherichia coli 93-001 | enterobacteria | 77.4 | 1 | Escherichia coli 93-001 hits |
| ....Escherichia coli PA3 | enterobacteria | 77.4 | 1 | Escherichia coli PA3 hits |
| ....Escherichia coli PA5 | enterobacteria | 77.4 | 1 | Escherichia coli PA5 hits |
| ....Escherichia coli PA9 | enterobacteria | 77.4 | 1 | Escherichia coli PA9 hits |
| ....Escherichia coli PA10 | enterobacteria | 77.4 | 1 | Escherichia coli PA10 hits |
| ....Escherichia coli PA14 | enterobacteria | 77.4 | 1 | Escherichia coli PA14 hits |
| ....Escherichia coli PA15 | enterobacteria | 77.4 | 1 | Escherichia coli PA15 hits |
| ....Escherichia coli PA25 | enterobacteria | 77.4 | 1 | Escherichia coli PA25 hits |
| ....Escherichia coli PA24 | enterobacteria | 77.4 | 1 | Escherichia coli PA24 hits |
| ....Escherichia coli PA28 | enterobacteria | 77.4 | 1 | Escherichia coli PA28 hits |
| ....Escherichia coli PA31 | enterobacteria | 77.4 | 1 | Escherichia coli PA31 hits |
| ....Escherichia coli PA32 | enterobacteria | 77.4 | 1 | Escherichia coli PA32 hits |
| ....Escherichia coli PA33 | enterobacteria | 77.4 | 1 | Escherichia coli PA33 hits |
| ....Escherichia coli PA39 | enterobacteria | 77.4 | 1 | Escherichia coli PA39 hits |
| ....Escherichia coli PA42 | enterobacteria | 77.4 | 1 | Escherichia coli PA42 hits |
| ....Escherichia coli TW07945 | enterobacteria | 77.4 | 1 | Escherichia coli TW07945 hits |
| ....Escherichia coli TW09098 | enterobacteria | 77.4 | 1 | Escherichia coli TW09098 hits |
| ....Escherichia coli TW09109 | enterobacteria | 77.4 | 1 | Escherichia coli TW09109 hits |
| ....Escherichia coli EC4203 | enterobacteria | 77.4 | 1 | Escherichia coli EC4203 hits |
| ....Escherichia coli EC4196 | enterobacteria | 77.4 | 1 | Escherichia coli EC4196 hits |
| ....Escherichia coli TW14301 | enterobacteria | 77.4 | 1 | Escherichia coli TW14301 hits |
| ....Escherichia coli EC4421 | enterobacteria | 77.4 | 1 | Escherichia coli EC4421 hits |
| ....Escherichia coli EC4422 | enterobacteria | 77.4 | 1 | Escherichia coli EC4422 hits |
| ....Escherichia coli EC4013 | enterobacteria | 77.4 | 1 | Escherichia coli EC4013 hits |
| ....Escherichia coli EC4402 | enterobacteria | 77.4 | 1 | Escherichia coli EC4402 hits |
| ....Escherichia coli EC4439 | enterobacteria | 77.4 | 1 | Escherichia coli EC4439 hits |
| ....Escherichia coli EC4436 | enterobacteria | 77.4 | 1 | Escherichia coli EC4436 hits |
| ....Escherichia coli EC4437 | enterobacteria | 77.4 | 1 | Escherichia coli EC4437 hits |
| ....Escherichia coli EC4448 | enterobacteria | 77.4 | 1 | Escherichia coli EC4448 hits |
| ....Escherichia coli EC1738 | enterobacteria | 77.4 | 1 | Escherichia coli EC1738 hits |
| ....Escherichia coli EC1734 | enterobacteria | 77.4 | 1 | Escherichia coli EC1734 hits |
| ....Escherichia coli EC1863 | enterobacteria | 77.4 | 1 | Escherichia coli EC1863 hits |
| ....Escherichia coli EC1845 | enterobacteria | 77.4 | 1 | Escherichia coli EC1845 hits |
| ....Escherichia coli PA7 | enterobacteria | 77.4 | 1 | Escherichia coli PA7 hits |
| ....Escherichia coli PA34 | enterobacteria | 77.4 | 1 | Escherichia coli PA34 hits |
| ....Escherichia coli FDA506 | enterobacteria | 77.4 | 1 | Escherichia coli FDA506 hits |
| ....Escherichia coli FDA507 | enterobacteria | 77.4 | 1 | Escherichia coli FDA507 hits |
| ....Escherichia coli FDA504 | enterobacteria | 77.4 | 1 | Escherichia coli FDA504 hits |
| ....Escherichia coli NE037 | enterobacteria | 77.4 | 1 | Escherichia coli NE037 hits |
| ....Escherichia coli PA4 | enterobacteria | 77.4 | 1 | Escherichia coli PA4 hits |
| ....Escherichia coli PA23 | enterobacteria | 77.4 | 1 | Escherichia coli PA23 hits |
| ....Escherichia coli PA49 | enterobacteria | 77.4 | 1 | Escherichia coli PA49 hits |
| ....Escherichia coli PA45 | enterobacteria | 77.4 | 1 | Escherichia coli PA45 hits |
| ....Escherichia coli TT12B | enterobacteria | 77.4 | 1 | Escherichia coli TT12B hits |
| ....Escherichia coli CB7326 | enterobacteria | 77.4 | 1 | Escherichia coli CB7326 hits |
| ....Escherichia coli EC96038 | enterobacteria | 77.4 | 1 | Escherichia coli EC96038 hits |
| ....Escherichia coli PA38 | enterobacteria | 77.4 | 1 | Escherichia coli PA38 hits |
| ....Escherichia coli EC1736 | enterobacteria | 77.4 | 1 | Escherichia coli EC1736 hits |
| ....Escherichia coli EC1846 | enterobacteria | 77.4 | 1 | Escherichia coli EC1846 hits |
| ....Escherichia coli EC1847 | enterobacteria | 77.4 | 1 | Escherichia coli EC1847 hits |
| ....Escherichia coli EC1848 | enterobacteria | 77.4 | 1 | Escherichia coli EC1848 hits |
| ....Escherichia coli EC1849 | enterobacteria | 77.4 | 1 | Escherichia coli EC1849 hits |
| ....Escherichia coli EC1850 | enterobacteria | 77.4 | 1 | Escherichia coli EC1850 hits |
| ....Escherichia coli EC1856 | enterobacteria | 77.4 | 1 | Escherichia coli EC1856 hits |
| ....Escherichia coli EC1862 | enterobacteria | 77.4 | 1 | Escherichia coli EC1862 hits |
| ....Escherichia coli EC1864 | enterobacteria | 77.4 | 1 | Escherichia coli EC1864 hits |
| ....Escherichia coli EC1868 | enterobacteria | 77.4 | 1 | Escherichia coli EC1868 hits |
| ....Escherichia coli EC1866 | enterobacteria | 77.4 | 1 | Escherichia coli EC1866 hits |
| ....Escherichia coli EC1869 | enterobacteria | 77.4 | 1 | Escherichia coli EC1869 hits |
| ....Escherichia coli EC1870 | enterobacteria | 77.4 | 1 | Escherichia coli EC1870 hits |
| ....Escherichia coli NE098 | enterobacteria | 77.4 | 1 | Escherichia coli NE098 hits |
| ....Escherichia coli FRIK523 | enterobacteria | 77.4 | 1 | Escherichia coli FRIK523 hits |
| ....Escherichia coli 0.1304 | enterobacteria | 77.4 | 1 | Escherichia coli 0.1304 hits |
| ....Escherichia coli 5.2239 | enterobacteria | 77.4 | 1 | Escherichia coli 5.2239 hits |
| ....Escherichia coli 3.4870 | enterobacteria | 77.4 | 1 | Escherichia coli 3.4870 hits |
| ....Escherichia coli 6.0172 | enterobacteria | 77.4 | 1 | Escherichia coli 6.0172 hits |
| ....Escherichia coli 8.0586 | enterobacteria | 77.4 | 1 | Escherichia coli 8.0586 hits |
| ....Escherichia coli 10.0833 | enterobacteria | 77.4 | 1 | Escherichia coli 10.0833 hits |
| ....Escherichia coli 8.2524 | enterobacteria | 77.4 | 1 | Escherichia coli 8.2524 hits |
| ....Escherichia coli 10.0869 | enterobacteria | 77.4 | 1 | Escherichia coli 10.0869 hits |
| ....Escherichia coli 10.0821 | enterobacteria | 77.4 | 1 | Escherichia coli 10.0821 hits |
| ....Escherichia coli 88.1042 | enterobacteria | 77.4 | 1 | Escherichia coli 88.1042 hits |
| ....Escherichia coli 89.0511 | enterobacteria | 77.4 | 1 | Escherichia coli 89.0511 hits |
| ....Escherichia coli 90.2281 | enterobacteria | 77.4 | 1 | Escherichia coli 90.2281 hits |
| ....Escherichia coli 93.0056 | enterobacteria | 77.4 | 1 | Escherichia coli 93.0056 hits |
| ....Escherichia coli 93.0055 | enterobacteria | 77.4 | 1 | Escherichia coli 93.0055 hits |
| ....Escherichia coli 94.0618 | enterobacteria | 77.4 | 1 | Escherichia coli 94.0618 hits |
| ....Escherichia coli 95.1288 | enterobacteria | 77.4 | 1 | Escherichia coli 95.1288 hits |
| ....Escherichia coli 96.0428 | enterobacteria | 77.4 | 1 | Escherichia coli 96.0428 hits |
| ....Escherichia coli 96.0427 | enterobacteria | 77.4 | 1 | Escherichia coli 96.0427 hits |
| ....Escherichia coli 96.0932 | enterobacteria | 77.4 | 1 | Escherichia coli 96.0932 hits |
| ....Escherichia coli 96.0107 | enterobacteria | 77.4 | 1 | Escherichia coli 96.0107 hits |
| ....Escherichia coli 97.0003 | enterobacteria | 77.4 | 1 | Escherichia coli 97.0003 hits |
| ....Escherichia coli 97.0007 | enterobacteria | 77.4 | 1 | Escherichia coli 97.0007 hits |
| ....Escherichia coli 99.0678 | enterobacteria | 77.4 | 1 | Escherichia coli 99.0678 hits |
| ....Escherichia coli 99.0713 | enterobacteria | 77.4 | 1 | Escherichia coli 99.0713 hits |
| ....Escherichia coli 96.0109 | enterobacteria | 77.4 | 1 | Escherichia coli 96.0109 hits |
| ....Escherichia coli 97.0010 | enterobacteria | 77.4 | 1 | Escherichia coli 97.0010 hits |
| ....Escherichia coli 09BKT078844 | enterobacteria | 77.4 | 1 | Escherichia coli 09BKT078844 hits |
| ....Escherichia coli 99.0839 | enterobacteria | 77.4 | 1 | Escherichia coli 99.0839 hits |
| ....Escherichia coli 99.0848 | enterobacteria | 77.4 | 1 | Escherichia coli 99.0848 hits |
| ....Escherichia coli PA11 | enterobacteria | 77.4 | 1 | Escherichia coli PA11 hits |
| ....Escherichia coli PA13 | enterobacteria | 77.4 | 1 | Escherichia coli PA13 hits |
| ....Escherichia coli PA19 | enterobacteria | 77.4 | 1 | Escherichia coli PA19 hits |
| ....Escherichia coli PA2 | enterobacteria | 77.4 | 1 | Escherichia coli PA2 hits |
| ....Escherichia coli PA47 | enterobacteria | 77.4 | 1 | Escherichia coli PA47 hits |
| ....Escherichia coli PA48 | enterobacteria | 77.4 | 1 | Escherichia coli PA48 hits |
| ....Escherichia coli PA8 | enterobacteria | 77.4 | 1 | Escherichia coli PA8 hits |
| ....Escherichia coli 7.1982 | enterobacteria | 77.4 | 1 | Escherichia coli 7.1982 hits |
| ....Escherichia coli 99.1762 | enterobacteria | 77.4 | 1 | Escherichia coli 99.1762 hits |
| ....Escherichia coli PA35 | enterobacteria | 77.4 | 1 | Escherichia coli PA35 hits |
| ....Escherichia coli 3.4880 | enterobacteria | 77.4 | 1 | Escherichia coli 3.4880 hits |
| ....Escherichia coli 95.0083 | enterobacteria | 77.4 | 1 | Escherichia coli 95.0083 hits |
| ....Escherichia coli 99.0670 | enterobacteria | 77.4 | 1 | Escherichia coli 99.0670 hits |
| ....Escherichia coli B28-1 | enterobacteria | 77.4 | 1 | Escherichia coli B28-1 hits |
| ....Escherichia coli B28-2 | enterobacteria | 77.4 | 1 | Escherichia coli B28-2 hits |
| ....Escherichia coli B29-1 | enterobacteria | 77.4 | 1 | Escherichia coli B29-1 hits |
| ....Escherichia coli B29-2 | enterobacteria | 77.4 | 1 | Escherichia coli B29-2 hits |
| ....Escherichia coli B36-1 | enterobacteria | 77.4 | 1 | Escherichia coli B36-1 hits |
| ....Escherichia coli B36-2 | enterobacteria | 77.4 | 1 | Escherichia coli B36-2 hits |
| ....Escherichia coli B7-1 | enterobacteria | 77.4 | 1 | Escherichia coli B7-1 hits |
| ....Escherichia coli B7-2 | enterobacteria | 77.4 | 1 | Escherichia coli B7-2 hits |
| ....Escherichia coli B93 | enterobacteria | 77.4 | 1 | Escherichia coli B93 hits |
| ....Escherichia coli B94 | enterobacteria | 77.4 | 1 | Escherichia coli B94 hits |
| ....Escherichia coli B95 | enterobacteria | 77.4 | 1 | Escherichia coli B95 hits |
| ....Escherichia coli Bd5610\_99 | enterobacteria | 77.4 | 1 | Escherichia coli Bd5610\_99 hits |
| ....Escherichia coli T1840\_97 | enterobacteria | 77.4 | 1 | Escherichia coli T1840\_97 hits |
| ....Escherichia coli T924\_01 | enterobacteria | 77.4 | 1 | Escherichia coli T924\_01 hits |
| ....Escherichia coli 2886-75 | enterobacteria | 77.4 | 1 | Escherichia coli 2886-75 hits |
| ....Escherichia coli B108 | enterobacteria | 77.4 | 1 | Escherichia coli B108 hits |
| ....Escherichia coli B109 | enterobacteria | 77.4 | 1 | Escherichia coli B109 hits |
| ....Escherichia coli B112 | enterobacteria | 77.4 | 1 | Escherichia coli B112 hits |
| ....Escherichia coli B113 | enterobacteria | 77.4 | 1 | Escherichia coli B113 hits |
| ....Escherichia coli B114 | enterobacteria | 77.4 | 1 | Escherichia coli B114 hits |
| ....Escherichia coli B15 | enterobacteria | 77.4 | 1 | Escherichia coli B15 hits |
| ....Escherichia coli B17 | enterobacteria | 77.4 | 1 | Escherichia coli B17 hits |
| ....Escherichia coli B5-2 | enterobacteria | 77.4 | 1 | Escherichia coli B5-2 hits |
| ....Escherichia coli 09BKT024447 | enterobacteria | 77.4 | 1 | Escherichia coli 09BKT024447 hits |
| ....Escherichia coli B89 | enterobacteria | 77.4 | 1 | Escherichia coli B89 hits |
| ....Escherichia coli B90 | enterobacteria | 77.4 | 1 | Escherichia coli B90 hits |
| ....Escherichia coli Tx1686 | enterobacteria | 77.4 | 1 | Escherichia coli Tx1686 hits |
| ....Escherichia coli Tx3800 | enterobacteria | 77.4 | 1 | Escherichia coli Tx3800 hits |
| ....Escherichia coli ATCC BAA-2192 | enterobacteria | 77.4 | 1 | Escherichia coli ATCC BAA-2192 hits |
| ....Escherichia coli O157: str. 2010EL-2045 | enterobacteria | 77.4 | 1 | Escherichia coli O157: str. 2010EL-2045 hits |
| ....Escherichia coli O157: str. 2010EL-2044 | enterobacteria | 77.4 | 1 | Escherichia coli O157: str. 2010EL-2044 hits |
| ....Escherichia coli SHECO003 | enterobacteria | 77.4 | 1 | Escherichia coli SHECO003 hits |
| ....Escherichia coli O157 | enterobacteria | 77.4 | 2 | Escherichia coli O157 hits |
| ....Escherichia coli O55:H7 str. USDA 5905 | enterobacteria | 77.4 | 2 | Escherichia coli O55:H7 str. USDA 5905 hits |
| ....Escherichia coli DEC5E | enterobacteria | 77.4 | 1 | Escherichia coli DEC5E hits |
| ....Escherichia coli 5905 | enterobacteria | 77.4 | 1 | Escherichia coli 5905 hits |
| ....Escherichia coli 09BKT076207 | enterobacteria | 77.4 | 1 | Escherichia coli 09BKT076207 hits |
| ....Escherichia coli O55:H7 str. CB9615 | enterobacteria | 77.4 | 2 | Escherichia coli O55:H7 str. CB9615 hits |
| ....Escherichia coli O55:H7 str. 3256-97 | enterobacteria | 77.4 | 1 | Escherichia coli O55:H7 str. 3256-97 hits |
| ....Escherichia coli DEC4A | enterobacteria | 77.4 | 1 | Escherichia coli DEC4A hits |
| ....Escherichia coli DEC5A | enterobacteria | 77.4 | 1 | Escherichia coli DEC5A hits |
| ....Escherichia coli DEC5B | enterobacteria | 77.4 | 1 | Escherichia coli DEC5B hits |
| ....Escherichia coli PA22 | enterobacteria | 77.4 | 1 | Escherichia coli PA22 hits |
| ....Escherichia coli TW10246 | enterobacteria | 77.4 | 1 | Escherichia coli TW10246 hits |
| ....Escherichia coli 8.0416 | enterobacteria | 77.4 | 1 | Escherichia coli 8.0416 hits |
| ....Escherichia coli O55:H7 str. 06-3555 | enterobacteria | 77.4 | 1 | Escherichia coli O55:H7 str. 06-3555 hits |
| ....Escherichia coli 88.1467 | enterobacteria | 77.4 | 1 | Escherichia coli 88.1467 hits |
| ....Escherichia coli TW11039 | enterobacteria | 77.4 | 1 | Escherichia coli TW11039 hits |
| ....Escherichia coli FRIK1997 | enterobacteria | 77.4 | 1 | Escherichia coli FRIK1997 hits |
| ....Escherichia coli EC1735 | enterobacteria | 77.4 | 1 | Escherichia coli EC1735 hits |
| ....Escherichia coli EC1737 | enterobacteria | 77.4 | 1 | Escherichia coli EC1737 hits |
| ....Escherichia coli 88.0221 | enterobacteria | 77.4 | 1 | Escherichia coli 88.0221 hits |
| ....Escherichia coli DEC4B | enterobacteria | 77.4 | 1 | Escherichia coli DEC4B hits |
| ....Escherichia coli ATCC 35150 | enterobacteria | 77.4 | 1 | Escherichia coli ATCC 35150 hits |
| ....Escherichia coli 97.1742 | enterobacteria | 77.4 | 1 | Escherichia coli 97.1742 hits |
| ....Escherichia coli DEC4D | enterobacteria | 77.4 | 1 | Escherichia coli DEC4D hits |
| ....Escherichia coli DEC3D | enterobacteria | 77.4 | 1 | Escherichia coli DEC3D hits |
| ....Escherichia coli DEC3E | enterobacteria | 77.4 | 1 | Escherichia coli DEC3E hits |
| ....Escherichia coli DEC4C | enterobacteria | 77.4 | 1 | Escherichia coli DEC4C hits |
| ...Escherichia coli | enterobacteria | 77.4 | 871 | Escherichia coli hits |
| ..Shigella boydii | enterobacteria | 77.4 | 3 | Shigella boydii hits |
| ..Shigella dysenteriae | enterobacteria | 77.4 | 1 | Shigella dysenteriae hits |
| ..Salmonella enterica subsp. enterica | enterobacteria | 77.4 | 1 | Salmonella enterica subsp. enterica hits |
| .synthetic construct | other sequences | 77.4 | 2 | synthetic construct hits |

## Organism Report

Lineage Report
Taxonomy Report

Organism Report

| Description | Score | E value | Accession |
| --- | --- | --- | --- |
| Escherichia coli O157:H7 str. EDL933 [enterobacteria]  Next Previous First | | | |
| --- | --- | --- | --- |
| Chain B, Crystal Structure Of Intimin-Tir90 Complex [Escherichia coli O157:H7 str. EDL933] | 77.4 | 3e-15 | 2ZWK\_B |
| Chain D, Crystal Structure Of Intimin-Tir90 Complex [Escherichia coli O157:H7 str. EDL933] | 77.4 | 3e-15 | 2ZWK\_D |
| Chain F, Crystal Structure Of Intimin-Tir90 Complex [Escherichia coli O157:H7 str. EDL933] | 77.4 | 3e-15 | 2ZWK\_F |
| L0027 [Escherichia coli O157:H7 str. EDL933] | 77.4 | 7e-15 | AAC31506 |
| putative translocated intimin receptor protein [Escherichia coli O157:H7 str. EDL933] | 77.4 | 7e-15 | AAG58825 |
| translocated intimin receptor Tir [Escherichia coli O157:H7 str. EDL933] | 77.4 | 7e-15 | AIG71090 |
| Escherichia coli O157:H7 [enterobacteria]  Next Previous First | | | |
| Chain C, Crystal Structure Of Intimin-Tir68 Complex [Escherichia coli O157:H7] | 77.4 | 4e-15 | 2ZQK\_C |
| Chain D, Crystal Structure Of Intimin-Tir68 Complex [Escherichia coli O157:H7] | 77.4 | 4e-15 | 2ZQK\_D |
| Chain M, Crystal Structure Of Intimin-Tir68 Complex [Escherichia coli O157:H7] | 77.4 | 4e-15 | 2ZQK\_M |
| Chain N, Crystal Structure Of Intimin-Tir68 Complex [Escherichia coli O157:H7] | 77.4 | 4e-15 | 2ZQK\_N |
| translocated intimin receptor Tir [Escherichia coli O157:H7] | 77.4 | 7e-15 | KKY45445 |
| type III secretion system LEE translocated intimin receptor Tir [Escherichia coli O157:H7] | 77.4 | 7e-15 | QAV75894 |
| translocated intimin receptor Tir [Escherichia coli O157:H7] | 77.4 | 7e-15 | ACG59623 |
| translocated intimin receptor Tir [Escherichia coli O157:H7] | 77.4 | 7e-15 | ANG71134 |
| translocated intimin receptor Tir [Escherichia coli O157:H7] | 77.4 | 7e-15 | ANG76633 |
| translocated intimin receptor Tir [Escherichia coli O157:H7] | 77.4 | 7e-15 | ANG82315 |
| translocated intimin receptor Tir [Escherichia coli O157:H7] | 77.4 | 7e-15 | ANW42615 |
| translocated intimin receptor Tir [Escherichia coli O157:H7] | 77.4 | 7e-15 | OPH49646 |
| translocated intimin receptor Tir [Escherichia coli O157:H7] | 77.4 | 7e-15 | OPH62768 |
| translocated intimin receptor Tir [Escherichia coli O157:H7] | 77.4 | 7e-15 | OPH68832 |
| type III secretion system LEE translocated intimin receptor Tir [Escherichia coli O157:H7] | 77.4 | 7e-15 | RXA10674 |
| RecName: Full=Translocated intimin receptor Tir; AltName: Full=Secreted effector protein Tir [Escherichia coli O157:H7] | 77.4 | 7e-15 | Q7DB77 |
| translocated intimin receptor Tir [Escherichia coli O157:H7] | 77.4 | 7e-15 | ALH92946 |
| translocated intimin receptor Tir [Escherichia coli O157:H7] | 77.4 | 7e-15 | AMG81031 |
| translocated intimin receptor Tir [Escherichia coli O157:H7] | 77.4 | 7e-15 | AOV19406 |
| translocated intimin receptor Tir [Escherichia coli O157:H7] | 77.4 | 7e-15 | AOV24760 |
| translocated intimin receptor Tir [Escherichia coli O157:H7] | 77.4 | 7e-15 | AOV30111 |
| translocated intimin receptor Tir [Escherichia coli O157:H7] | 77.4 | 7e-15 | AOV35478 |
| translocated intimin receptor Tir [Escherichia coli O157:H7] | 77.4 | 7e-15 | AOV40891 |
| translocated intimin receptor Tir [Escherichia coli O157:H7] | 77.4 | 7e-15 | AOV46236 |
| translocated intimin receptor Tir [Escherichia coli O157:H7] | 77.4 | 7e-15 | AOV51651 |
| type III secretion system LEE translocated intimin receptor Tir [Escherichia coli O157:H7] | 77.4 | 7e-15 | AYV43791 |
| translocated intimin receptor Tir [Escherichia coli O157:H7] | 77.4 | 7e-15 | KKF85659 |
| translocated intimin receptor Tir [Escherichia coli O157:H7] | 77.4 | 7e-15 | KRQ06022 |
| translocated intimin receptor Tir [Escherichia coli O157:H7] | 77.4 | 7e-15 | OAN06989 |
| type III secretion system LEE translocated intimin receptor Tir [Escherichia coli O157:H7] | 77.4 | 7e-15 | QCH80447 |
| type III secretion system LEE translocated intimin receptor Tir [Escherichia coli O157:H7] | 77.4 | 7e-15 | QCV05266 |
| type III secretion system LEE translocated intimin receptor Tir [Escherichia coli O157:H7] | 77.4 | 7e-15 | QCV15327 |
| type III secretion system LEE translocated intimin receptor Tir [Escherichia coli O157:H7] | 77.4 | 7e-15 | QDG07136 |
| type III secretion system LEE translocated intimin receptor Tir [Escherichia coli O157:H7] | 77.4 | 7e-15 | RNG05880 |
| type III secretion system LEE translocated intimin receptor Tir [Escherichia coli O157:H7] | 77.4 | 7e-15 | RYC22411 |
| T3SS translocated intimin receptor Tir [Escherichia coli O157:H7] | 77.4 | 7e-15 | BBC52819 |
| translocated intimin receptor Tir [Escherichia coli O157:H7] | 77.4 | 7e-15 | KKF75760 |
| Escherichia coli [enterobacteria]  Next Previous First | | | |
| type III secretion system LEE translocated intimin receptor Tir [Escherichia coli] | 77.4 | 7e-15 | WP\_052913078 |
| type III secretion system LEE translocated intimin receptor Tir [Escherichia coli] | 77.4 | 7e-15 | WP\_137526993 |
| T3SS translocated intimin receptor Tir [Escherichia coli] | 77.4 | 7e-15 | GDG48597 |
| type III secretion system LEE translocated intimin receptor Tir [Escherichia coli] | 77.4 | 7e-15 | WP\_052893799 |
| type III secretion system LEE translocated intimin receptor Tir [Escherichia coli] | 77.4 | 7e-15 | WP\_024240337 |
| translocated intimin receptor [Escherichia coli] | 77.4 | 7e-15 | AAX47730 |
| type III secretion system LEE translocated intimin receptor Tir [Escherichia coli] | 77.4 | 7e-15 | AVV77533 |
| type III secretion system LEE translocated intimin receptor Tir [Escherichia coli] | 77.4 | 7e-15 | EAB6055658 |
| translocated intimin receptor Tir [Escherichia coli] | 77.4 | 7e-15 | KYR40417 |
| translocated intimin receptor Tir [Escherichia coli] | 77.4 | 7e-15 | KYR57611 |
| translocated intimin receptor Tir [Escherichia coli] | 77.4 | 7e-15 | KYU02021 |
| translocated intimin receptor Tir [Escherichia coli] | 77.4 | 7e-15 | OTD12495 |
| type III secretion system LEE translocated intimin receptor Tir [Escherichia coli] | 77.4 | 7e-15 | QCD71644 |
| type III secretion system LEE translocated intimin receptor Tir [Escherichia coli] | 77.4 | 7e-15 | QCD76135 |
| type III secretion system LEE translocated intimin receptor Tir [Escherichia coli] | 77.4 | 7e-15 | WP\_107188580 |
| type III secretion system LEE translocated intimin receptor Tir [Escherichia coli] | 77.4 | 7e-15 | PSX47783 |
| type III secretion system LEE translocated intimin receptor Tir [Escherichia coli] | 77.4 | 7e-15 | WP\_052924572 |
| type III secretion system LEE translocated intimin receptor Tir [Escherichia coli] | 77.4 | 7e-15 | WP\_075860487 |
| type III secretion system LEE translocated intimin receptor Tir [Escherichia coli] | 77.4 | 7e-15 | WP\_021500921 |
| type III secretion system LEE translocated intimin receptor Tir [Escherichia coli] | 77.4 | 7e-15 | EAA1370227 |
| type III secretion system LEE translocated intimin receptor Tir [Escherichia coli] | 77.4 | 7e-15 | WP\_080855615 |
| type III secretion system LEE translocated intimin receptor Tir [Escherichia coli] | 77.4 | 7e-15 | WP\_121349350 |
| type III secretion system LEE translocated intimin receptor Tir [Escherichia coli] | 77.4 | 7e-15 | WP\_024231660 |
| translocated intimin receptor Tir [Escherichia coli] | 77.4 | 7e-15 | KYT20252 |
| type III secretion system LEE translocated intimin receptor Tir [Escherichia coli] | 77.4 | 7e-15 | PWR55429 |
| translocated intimin receptor Tir [Escherichia coli] | 77.4 | 7e-15 | BAF92845 |
| translocated intimin receptor Tir [Escherichia coli] | 77.4 | 7e-15 | CTR29780 |
| translocated intimin receptor Tir [Escherichia coli] | 77.4 | 7e-15 | CTU03478 |
| type III secretion system LEE translocated intimin receptor Tir [Escherichia coli] | 77.4 | 7e-15 | WP\_074464449 |
| type III secretion system LEE translocated intimin receptor Tir [Escherichia coli] | 77.4 | 7e-15 | WP\_001689362 |
| type III secretion system LEE translocated intimin receptor Tir [Escherichia coli] | 77.4 | 7e-15 | WP\_001509343 |
| translocated intimin receptor Tir [Escherichia coli] | 77.4 | 7e-15 | KKK30827 |
| translocated intimin receptor Tir [Escherichia coli] | 77.4 | 7e-15 | KPH39355 |
| type III secretion system LEE translocated intimin receptor Tir [Escherichia coli] | 77.4 | 7e-15 | MMB71790 |
| type III secretion system LEE translocated intimin receptor Tir [Escherichia coli] | 77.4 | 7e-15 | PDV11604 |
| type III secretion system LEE translocated intimin receptor Tir [Escherichia coli] | 77.4 | 7e-15 | RDH69431 |
| type III secretion system LEE translocated intimin receptor Tir [Escherichia coli] | 77.4 | 7e-15 | TJC95742 |
| type III secretion system LEE translocated intimin receptor Tir [Escherichia coli] | 77.4 | 7e-15 | WP\_001302942 |
| translocated intimin receptor Tir [Escherichia coli] | 77.4 | 7e-15 | API28647 |
| type III secretion system LEE translocated intimin receptor Tir [Escherichia coli] | 77.4 | 7e-15 | EAA0657069 |
| type III secretion system LEE translocated intimin receptor Tir [Escherichia coli] | 77.4 | 7e-15 | EAA0991685 |
| type III secretion system LEE translocated intimin receptor Tir [Escherichia coli] | 77.4 | 7e-15 | EAA1551151 |
| type III secretion system LEE translocated intimin receptor Tir [Escherichia coli] | 77.4 | 7e-15 | EAA1690080 |
| type III secretion system LEE translocated intimin receptor Tir [Escherichia coli] | 77.4 | 7e-15 | EAA1786492 |
| type III secretion system LEE translocated intimin receptor Tir [Escherichia coli] | 77.4 | 7e-15 | EAA2429909 |
| type III secretion system LEE translocated intimin receptor Tir [Escherichia coli] | 77.4 | 7e-15 | EAA2626994 |
| type III secretion system LEE translocated intimin receptor Tir [Escherichia coli] | 77.4 | 7e-15 | EAA2859956 |
| type III secretion system LEE translocated intimin receptor Tir [Escherichia coli] | 77.4 | 7e-15 | EAA3448985 |
| type III secretion system LEE translocated intimin receptor Tir [Escherichia coli] | 77.4 | 7e-15 | EAA3480380 |
| type III secretion system LEE translocated intimin receptor Tir [Escherichia coli] | 77.4 | 7e-15 | EAA6021187 |
| type III secretion system LEE translocated intimin receptor Tir [Escherichia coli] | 77.4 | 7e-15 | EAA6555249 |
| type III secretion system LEE translocated intimin receptor Tir [Escherichia coli] | 77.4 | 7e-15 | EAB0413355 |
| type III secretion system LEE translocated intimin receptor Tir [Escherichia coli] | 77.4 | 7e-15 | EAB0448180 |
| type III secretion system LEE translocated intimin receptor Tir [Escherichia coli] | 77.4 | 7e-15 | EAB0551229 |
| type III secretion system LEE translocated intimin receptor Tir [Escherichia coli] | 77.4 | 7e-15 | EAB0569213 |
| type III secretion system LEE translocated intimin receptor Tir [Escherichia coli] | 77.4 | 7e-15 | EAB0899903 |
| type III secretion system LEE translocated intimin receptor Tir [Escherichia coli] | 77.4 | 7e-15 | EAB0972883 |
| type III secretion system LEE translocated intimin receptor Tir [Escherichia coli] | 77.4 | 7e-15 | EAB1167039 |
| type III secretion system LEE translocated intimin receptor Tir [Escherichia coli] | 77.4 | 7e-15 | EAB1183048 |
| type III secretion system LEE translocated intimin receptor Tir [Escherichia coli] | 77.4 | 7e-15 | EAB1340699 |
| type III secretion system LEE translocated intimin receptor Tir [Escherichia coli] | 77.4 | 7e-15 | EAB6316642 |
| type III secretion system LEE translocated intimin receptor Tir [Escherichia coli] | 77.4 | 7e-15 | EAB6392670 |
| type III secretion system LEE translocated intimin receptor Tir [Escherichia coli] | 77.4 | 7e-15 | EAB6764227 |
| type III secretion system LEE translocated intimin receptor Tir [Escherichia coli] | 77.4 | 7e-15 | EAB7246311 |
| type III secretion system LEE translocated intimin receptor Tir [Escherichia coli] | 77.4 | 7e-15 | EAB7837403 |
| type III secretion system LEE translocated intimin receptor Tir [Escherichia coli] | 77.4 | 7e-15 | EAB8186946 |
| type III secretion system LEE translocated intimin receptor Tir [Escherichia coli] | 77.4 | 7e-15 | EAB9010298 |
| type III secretion system LEE translocated intimin receptor Tir [Escherichia coli] | 77.4 | 7e-15 | EAB9665950 |
| type III secretion system LEE translocated intimin receptor Tir [Escherichia coli] | 77.4 | 7e-15 | EAC0751089 |
| type III secretion system LEE translocated intimin receptor Tir [Escherichia coli] | 77.4 | 7e-15 | EAC0834017 |
| type III secretion system LEE translocated intimin receptor Tir [Escherichia coli] | 77.4 | 7e-15 | EAC1029061 |
| type III secretion system LEE translocated intimin receptor Tir [Escherichia coli] | 77.4 | 7e-15 | EAC1140562 |
| translocated intimin receptor Tir [Escherichia coli] | 77.4 | 7e-15 | KOZ16934 |
| translocated intimin receptor Tir [Escherichia coli] | 77.4 | 7e-15 | KPH43008 |
| type III secretion system LEE translocated intimin receptor Tir [Escherichia coli] | 77.4 | 7e-15 | MDL31379 |
| type III secretion system LEE translocated intimin receptor Tir [Escherichia coli] | 77.4 | 7e-15 | MDL55200 |
| type III secretion system LEE translocated intimin receptor Tir [Escherichia coli] | 77.4 | 7e-15 | MDO72625 |
| type III secretion system LEE translocated intimin receptor Tir [Escherichia coli] | 77.4 | 7e-15 | MEA40658 |
| type III secretion system LEE translocated intimin receptor Tir [Escherichia coli] | 77.4 | 7e-15 | MES81963 |
| type III secretion system LEE translocated intimin receptor Tir [Escherichia coli] | 77.4 | 7e-15 | MET65870 |
| type III secretion system LEE translocated intimin receptor Tir [Escherichia coli] | 77.4 | 7e-15 | MFD30863 |
| type III secretion system LEE translocated intimin receptor Tir [Escherichia coli] | 77.4 | 7e-15 | MFR22270 |
| type III secretion system LEE translocated intimin receptor Tir [Escherichia coli] | 77.4 | 7e-15 | MFV70666 |
| type III secretion system LEE translocated intimin receptor Tir [Escherichia coli] | 77.4 | 7e-15 | MFX18305 |
| type III secretion system LEE translocated intimin receptor Tir [Escherichia coli] | 77.4 | 7e-15 | MFZ39942 |
| type III secretion system LEE translocated intimin receptor Tir [Escherichia coli] | 77.4 | 7e-15 | MFZ57058 |
| type III secretion system LEE translocated intimin receptor Tir [Escherichia coli] | 77.4 | 7e-15 | MFZ60990 |
| type III secretion system LEE translocated intimin receptor Tir [Escherichia coli] | 77.4 | 7e-15 | MHL23201 |
| type III secretion system LEE translocated intimin receptor Tir [Escherichia coli] | 77.4 | 7e-15 | MJD41925 |
| type III secretion system LEE translocated intimin receptor Tir [Escherichia coli] | 77.4 | 7e-15 | MJF28839 |
| type III secretion system LEE translocated intimin receptor Tir [Escherichia coli] | 77.4 | 7e-15 | MJH42807 |
| type III secretion system LEE translocated intimin receptor Tir [Escherichia coli] | 77.4 | 7e-15 | MJI00433 |
| type III secretion system LEE translocated intimin receptor Tir [Escherichia coli] | 77.4 | 7e-15 | MJL83782 |
| type III secretion system LEE translocated intimin receptor Tir [Escherichia coli] | 77.4 | 7e-15 | MKF15412 |
| type III secretion system LEE translocated intimin receptor Tir [Escherichia coli] | 77.4 | 7e-15 | MKL38725 |
| type III secretion system LEE translocated intimin receptor Tir [Escherichia coli] | 77.4 | 7e-15 | MKM12620 |
| type III secretion system LEE translocated intimin receptor Tir [Escherichia coli] | 77.4 | 7e-15 | MKN47016 |
| type III secretion system LEE translocated intimin receptor Tir [Escherichia coli] | 77.4 | 7e-15 | MLK46122 |
| type III secretion system LEE translocated intimin receptor Tir [Escherichia coli] | 77.4 | 7e-15 | MLN80726 |
| type III secretion system LEE translocated intimin receptor Tir [Escherichia coli] | 77.4 | 7e-15 | MLS30438 |
| type III secretion system LEE translocated intimin receptor Tir [Escherichia coli] | 77.4 | 7e-15 | MMA69299 |
| type III secretion system LEE translocated intimin receptor Tir [Escherichia coli] | 77.4 | 7e-15 | MMJ70980 |
| type III secretion system LEE translocated intimin receptor Tir [Escherichia coli] | 77.4 | 7e-15 | MMR35826 |
| type III secretion system LEE translocated intimin receptor Tir [Escherichia coli] | 77.4 | 7e-15 | MMU15294 |
| translocated intimin receptor Tir [Escherichia coli] | 77.4 | 7e-15 | OEB94511 |
| translocated intimin receptor Tir [Escherichia coli] | 77.4 | 7e-15 | OTD34146 |
| translocated intimin receptor Tir [Escherichia coli] | 77.4 | 7e-15 | OVB20103 |
| translocated intimin receptor Tir [Escherichia coli] | 77.4 | 7e-15 | OVB36925 |
| translocated intimin receptor Tir [Escherichia coli] | 77.4 | 7e-15 | OVB40783 |
| translocated intimin receptor Tir [Escherichia coli] | 77.4 | 7e-15 | OVB41360 |
| translocated intimin receptor Tir [Escherichia coli] | 77.4 | 7e-15 | OVB57745 |
| translocated intimin receptor Tir [Escherichia coli] | 77.4 | 7e-15 | OVD05755 |
| translocated intimin receptor Tir [Escherichia coli] | 77.4 | 7e-15 | OVD15700 |
| translocated intimin receptor Tir [Escherichia coli] | 77.4 | 7e-15 | OVD25582 |
| translocated intimin receptor Tir [Escherichia coli] | 77.4 | 7e-15 | OVD31031 |
| translocated intimin receptor Tir [Escherichia coli] | 77.4 | 7e-15 | OVD31634 |
| translocated intimin receptor Tir [Escherichia coli] | 77.4 | 7e-15 | OVD42307 |
| translocated intimin receptor Tir [Escherichia coli] | 77.4 | 7e-15 | OVD47906 |
| translocated intimin receptor Tir [Escherichia coli] | 77.4 | 7e-15 | OVD49174 |
| translocated intimin receptor Tir [Escherichia coli] | 77.4 | 7e-15 | OVD67025 |
| translocated intimin receptor Tir [Escherichia coli] | 77.4 | 7e-15 | OVD77781 |
| translocated intimin receptor Tir [Escherichia coli] | 77.4 | 7e-15 | OVD80992 |
| translocated intimin receptor Tir [Escherichia coli] | 77.4 | 7e-15 | OVD95102 |
| translocated intimin receptor Tir [Escherichia coli] | 77.4 | 7e-15 | OVD99682 |
| type III secretion system LEE translocated intimin receptor Tir [Escherichia coli] | 77.4 | 7e-15 | QCL18216 |
| type III secretion system LEE translocated intimin receptor Tir [Escherichia coli] | 77.4 | 7e-15 | QCL40248 |
| type III secretion system LEE translocated intimin receptor Tir [Escherichia coli] | 77.4 | 7e-15 | RDH57674 |
| type III secretion system LEE translocated intimin receptor Tir [Escherichia coli] | 77.4 | 7e-15 | RDH68597 |
| type III secretion system LEE translocated intimin receptor Tir [Escherichia coli] | 77.4 | 7e-15 | TJD03967 |
| type III secretion system LEE translocated intimin receptor Tir [Escherichia coli] | 77.4 | 7e-15 | TJG22445 |
| T3SS translocated intimin receptor Tir [Escherichia coli] | 77.4 | 7e-15 | GDL10707 |
| translocated intimin receptor Tir [Escherichia coli] | 77.4 | 7e-15 | STE36073 |
| translocated intimin receptor Tir [Escherichia coli] | 77.4 | 7e-15 | STQ26944 |
| type III secretion system LEE translocated intimin receptor Tir [Escherichia coli] | 77.4 | 7e-15 | WP\_053888367 |
| translocated intimin receptor Tir [Escherichia coli] | 77.4 | 7e-15 | CTT53503 |
| type III secretion system LEE translocated intimin receptor Tir [Escherichia coli] | 77.4 | 7e-15 | WP\_069199550 |
| type III secretion system LEE translocated intimin receptor Tir [Escherichia coli] | 77.4 | 7e-15 | MDT83775 |
| type III secretion system LEE translocated intimin receptor Tir [Escherichia coli] | 77.4 | 7e-15 | MEZ50559 |
| type III secretion system LEE translocated intimin receptor Tir [Escherichia coli] | 77.4 | 7e-15 | WP\_001419236 |
| type III secretion system LEE translocated intimin receptor Tir [Escherichia coli] | 77.4 | 7e-15 | WP\_115455538 |
| type III secretion system LEE translocated intimin receptor Tir [Escherichia coli] | 77.4 | 7e-15 | WP\_074452394 |
| type III secretion system LEE translocated intimin receptor Tir [Escherichia coli] | 77.4 | 7e-15 | WP\_053901904 |
| translocated intimin receptor Tir [Escherichia coli] | 77.4 | 7e-15 | KOZ23365 |
| type III secretion system LEE translocated intimin receptor Tir [Escherichia coli] | 77.4 | 7e-15 | WP\_001685899 |
| type III secretion system LEE translocated intimin receptor Tir [Escherichia coli] | 77.4 | 7e-15 | MGW39154 |
| type III secretion system LEE translocated intimin receptor Tir [Escherichia coli] | 77.4 | 7e-15 | WP\_097209363 |
| type III secretion system LEE translocated intimin receptor Tir [Escherichia coli] | 77.4 | 7e-15 | EAB9759676 |
| translocated intimin receptor Tir [Escherichia coli] | 77.4 | 7e-15 | RFQ79236 |
| type III secretion system LEE translocated intimin receptor Tir [Escherichia coli] | 77.4 | 7e-15 | WP\_001507205 |
| type III secretion system LEE translocated intimin receptor Tir [Escherichia coli] | 77.4 | 7e-15 | EAB0936127 |
| type III secretion system LEE translocated intimin receptor Tir [Escherichia coli] | 77.4 | 7e-15 | EAB1390510 |
| translocated intimin receptor Tir [Escherichia coli] | 77.4 | 7e-15 | KPP37030 |
| type III secretion system LEE translocated intimin receptor Tir [Escherichia coli] | 77.4 | 7e-15 | MFA24395 |
| type III secretion system LEE translocated intimin receptor Tir [Escherichia coli] | 77.4 | 7e-15 | MFC88214 |
| translocated intimin receptor Tir [Escherichia coli] | 77.4 | 7e-15 | OTU99492 |
| translocated intimin receptor Tir [Escherichia coli] | 77.4 | 7e-15 | OTV08453 |
| type III secretion system LEE translocated intimin receptor Tir [Escherichia coli] | 77.4 | 7e-15 | TGG11220 |
| type III secretion system LEE translocated intimin receptor Tir [Escherichia coli] | 77.4 | 7e-15 | TGG24906 |
| type III secretion system LEE translocated intimin receptor Tir [Escherichia coli] | 77.4 | 7e-15 | TGG37765 |
| type III secretion system LEE translocated intimin receptor Tir [Escherichia coli] | 77.4 | 7e-15 | TGJ90995 |
| type III secretion system LEE translocated intimin receptor Tir [Escherichia coli] | 77.4 | 7e-15 | TJC69240 |
| translocated intimin receptor Tir [Escherichia coli] | 77.4 | 7e-15 | ACG59737 |
| type III secretion system LEE translocated intimin receptor Tir [Escherichia coli] | 77.4 | 7e-15 | WP\_137548124 |
| T3SS translocated intimin receptor Tir [Escherichia coli] | 77.4 | 7e-15 | GDP69936 |
| T3SS translocated intimin receptor Tir [Escherichia coli] | 77.4 | 7e-15 | GDV08525 |
| type III secretion system LEE translocated intimin receptor Tir [Escherichia coli] | 77.4 | 7e-15 | WP\_032316466 |
| type III secretion system LEE translocated intimin receptor Tir [Escherichia coli] | 77.4 | 7e-15 | WP\_001401250 |
| type III secretion system LEE translocated intimin receptor Tir [Escherichia coli] | 77.4 | 7e-15 | EAA0859685 |
| type III secretion system LEE translocated intimin receptor Tir [Escherichia coli] | 77.4 | 7e-15 | EAA2529720 |
| type III secretion system LEE translocated intimin receptor Tir [Escherichia coli] | 77.4 | 7e-15 | EAB0423077 |
| type III secretion system LEE translocated intimin receptor Tir [Escherichia coli] | 77.4 | 7e-15 | EAB0547476 |
| type III secretion system LEE translocated intimin receptor Tir [Escherichia coli] | 77.4 | 7e-15 | EAB0716069 |
| type III secretion system LEE translocated intimin receptor Tir [Escherichia coli] | 77.4 | 7e-15 | EAC0676640 |
| translocated intimin receptor Tir [Escherichia coli] | 77.4 | 7e-15 | KDM80760 |
| translocated intimin receptor Tir [Escherichia coli] | 77.4 | 7e-15 | KJW46392 |
| translocated intimin receptor Tir [Escherichia coli] | 77.4 | 7e-15 | KJW50862 |
| translocated intimin receptor Tir [Escherichia coli] | 77.4 | 7e-15 | KLG53658 |
| translocated intimin receptor Tir [Escherichia coli] | 77.4 | 7e-15 | KNF17046 |
| translocated intimin receptor Tir [Escherichia coli] | 77.4 | 7e-15 | KNF17570 |
| translocated intimin receptor Tir [Escherichia coli] | 77.4 | 7e-15 | KNF48830 |
| translocated intimin receptor Tir [Escherichia coli] | 77.4 | 7e-15 | KPP00118 |
| translocated intimin receptor Tir [Escherichia coli] | 77.4 | 7e-15 | KPP13101 |
| translocated intimin receptor Tir [Escherichia coli] | 77.4 | 7e-15 | KYR24630 |
| translocated intimin receptor Tir [Escherichia coli] | 77.4 | 7e-15 | KYS41869 |
| type III secretion system LEE translocated intimin receptor Tir [Escherichia coli] | 77.4 | 7e-15 | MDQ00425 |
| type III secretion system LEE translocated intimin receptor Tir [Escherichia coli] | 77.4 | 7e-15 | MEW66312 |
| type III secretion system LEE translocated intimin receptor Tir [Escherichia coli] | 77.4 | 7e-15 | MEY82878 |
| type III secretion system LEE translocated intimin receptor Tir [Escherichia coli] | 77.4 | 7e-15 | MEZ54072 |
| type III secretion system LEE translocated intimin receptor Tir [Escherichia coli] | 77.4 | 7e-15 | MFB69835 |
| type III secretion system LEE translocated intimin receptor Tir [Escherichia coli] | 77.4 | 7e-15 | MFD12875 |
| type III secretion system LEE translocated intimin receptor Tir [Escherichia coli] | 77.4 | 7e-15 | MFD49357 |
| type III secretion system LEE translocated intimin receptor Tir [Escherichia coli] | 77.4 | 7e-15 | MFS62090 |
| type III secretion system LEE translocated intimin receptor Tir [Escherichia coli] | 77.4 | 7e-15 | MFY65665 |
| type III secretion system LEE translocated intimin receptor Tir [Escherichia coli] | 77.4 | 7e-15 | MFZ82232 |
| type III secretion system LEE translocated intimin receptor Tir [Escherichia coli] | 77.4 | 7e-15 | MGZ44203 |
| type III secretion system LEE translocated intimin receptor Tir [Escherichia coli] | 77.4 | 7e-15 | MKI95435 |
| type III secretion system LEE translocated intimin receptor Tir [Escherichia coli] | 77.4 | 7e-15 | MLV71769 |
| type III secretion system LEE translocated intimin receptor Tir [Escherichia coli] | 77.4 | 7e-15 | MMF67343 |
| type III secretion system LEE translocated intimin receptor Tir [Escherichia coli] | 77.4 | 7e-15 | MMP12018 |
| type III secretion system LEE translocated intimin receptor Tir [Escherichia coli] | 77.4 | 7e-15 | PDV74335 |
| type III secretion system LEE translocated intimin receptor Tir [Escherichia coli] | 77.4 | 7e-15 | TJL89599 |
| type III secretion system LEE translocated intimin receptor Tir [Escherichia coli] | 77.4 | 7e-15 | TJS52695 |
| type III secretion system LEE translocated intimin receptor Tir [Escherichia coli] | 77.4 | 7e-15 | TJT64693 |
| type III secretion system LEE translocated intimin receptor Tir [Escherichia coli] | 77.4 | 7e-15 | TJT70848 |
| type III secretion system LEE translocated intimin receptor Tir [Escherichia coli] | 77.4 | 7e-15 | TJT92425 |
| T3SS translocated intimin receptor Tir [Escherichia coli] | 77.4 | 7e-15 | GDH52009 |
| T3SS translocated intimin receptor Tir [Escherichia coli] | 77.4 | 7e-15 | GDL41249 |
| T3SS translocated intimin receptor Tir [Escherichia coli] | 77.4 | 7e-15 | GDV02581 |
| translocated intimin receptor Tir [Escherichia coli] | 77.4 | 7e-15 | SPX28836 |
| type III secretion system LEE translocated intimin receptor Tir [Escherichia coli] | 77.4 | 7e-15 | WP\_096942567 |
| type III secretion system LEE translocated intimin receptor Tir [Escherichia coli] | 77.4 | 7e-15 | QCL57131 |
| type III secretion system LEE translocated intimin receptor Tir [Escherichia coli] | 77.4 | 7e-15 | QCL62619 |
| type III secretion system LEE translocated intimin receptor Tir [Escherichia coli] | 77.4 | 7e-15 | QCM14516 |
| type III secretion system LEE translocated intimin receptor Tir [Escherichia coli] | 77.4 | 7e-15 | QCM20005 |
| type III secretion system LEE translocated intimin receptor Tir [Escherichia coli] | 77.4 | 7e-15 | QCM25494 |
| type III secretion system LEE translocated intimin receptor Tir [Escherichia coli] | 77.4 | 7e-15 | QCM30983 |
| type III secretion system LEE translocated intimin receptor Tir [Escherichia coli] | 77.4 | 7e-15 | QCM36470 |
| type III secretion system LEE translocated intimin receptor Tir [Escherichia coli] | 77.4 | 7e-15 | QCM41957 |
| type III secretion system LEE translocated intimin receptor Tir [Escherichia coli] | 77.4 | 7e-15 | QCM47442 |
| type III secretion system LEE translocated intimin receptor Tir [Escherichia coli] | 77.4 | 7e-15 | QCM52928 |
| type III secretion system LEE translocated intimin receptor Tir [Escherichia coli] | 77.4 | 7e-15 | QCM58415 |
| type III secretion system LEE translocated intimin receptor Tir [Escherichia coli] | 77.4 | 7e-15 | QCM63903 |
| type III secretion system LEE translocated intimin receptor Tir [Escherichia coli] | 77.4 | 7e-15 | QCM85823 |
| type III secretion system LEE translocated intimin receptor Tir [Escherichia coli] | 77.4 | 7e-15 | QCM91313 |
| type III secretion system LEE translocated intimin receptor Tir [Escherichia coli] | 77.4 | 7e-15 | QCM96801 |
| type III secretion system LEE translocated intimin receptor Tir [Escherichia coli] | 77.4 | 7e-15 | QCN02290 |
| type III secretion system LEE translocated intimin receptor Tir [Escherichia coli] | 77.4 | 7e-15 | QCN07775 |
| type III secretion system LEE translocated intimin receptor Tir [Escherichia coli] | 77.4 | 7e-15 | QCN13263 |
| type III secretion system LEE translocated intimin receptor Tir [Escherichia coli] | 77.4 | 7e-15 | QCN18749 |
| type III secretion system LEE translocated intimin receptor Tir [Escherichia coli] | 77.4 | 7e-15 | QCN24159 |
| type III secretion system LEE translocated intimin receptor Tir [Escherichia coli] | 77.4 | 7e-15 | QCN29645 |
| type III secretion system LEE translocated intimin receptor Tir [Escherichia coli] | 77.4 | 7e-15 | WP\_136159100 |
| Tir, partial [Escherichia coli] | 77.4 | 7e-15 | ABE65381 |
| type III secretion system LEE translocated intimin receptor Tir [Escherichia coli] | 77.4 | 7e-15 | WP\_001359017 |
| translocated intimin receptor Tir [Escherichia coli] | 77.4 | 7e-15 | KJJ46919 |
| translocated intimin receptor Tir [Escherichia coli] | 77.4 | 7e-15 | KOZ65107 |
| translocated intimin receptor Tir [Escherichia coli] | 77.4 | 7e-15 | KOZ71389 |
| translocated intimin receptor Tir [Escherichia coli] | 77.4 | 7e-15 | KOZ78498 |
| translocated intimin receptor Tir [Escherichia coli] | 77.4 | 7e-15 | KOZ85526 |
| translocated intimin receptor Tir [Escherichia coli] | 77.4 | 7e-15 | KOZ86923 |
| type III secretion system LEE translocated intimin receptor Tir [Escherichia coli] | 77.4 | 7e-15 | MMZ22225 |
| translocated intimin receptor Tir [Escherichia coli] | 77.4 | 7e-15 | OTE03588 |
| type III secretion system LEE translocated intimin receptor Tir [Escherichia coli] | 77.4 | 7e-15 | EAB0833668 |
| type III secretion system LEE translocated intimin receptor Tir [Escherichia coli] | 77.4 | 7e-15 | WP\_001457743 |
| translocated intimin receptor Tir [Escherichia coli] | 77.4 | 7e-15 | API23152 |
| type III secretion system LEE translocated intimin receptor Tir [Escherichia coli] | 77.4 | 7e-15 | EAA2212869 |
| type III secretion system LEE translocated intimin receptor Tir [Escherichia coli] | 77.4 | 7e-15 | EAA3075855 |
| type III secretion system LEE translocated intimin receptor Tir [Escherichia coli] | 77.4 | 7e-15 | EAA3521398 |
| type III secretion system LEE translocated intimin receptor Tir [Escherichia coli] | 77.4 | 7e-15 | EAA4866853 |
| type III secretion system LEE translocated intimin receptor Tir [Escherichia coli] | 77.4 | 7e-15 | EAA6152894 |
| type III secretion system LEE translocated intimin receptor Tir [Escherichia coli] | 77.4 | 7e-15 | EAB0989559 |
| type III secretion system LEE translocated intimin receptor Tir [Escherichia coli] | 77.4 | 7e-15 | EAB6012116 |
| type III secretion system LEE translocated intimin receptor Tir [Escherichia coli] | 77.4 | 7e-15 | EAB6097082 |
| type III secretion system LEE translocated intimin receptor Tir [Escherichia coli] | 77.4 | 7e-15 | EAB6102665 |
| type III secretion system LEE translocated intimin receptor Tir [Escherichia coli] | 77.4 | 7e-15 | EAB6398162 |
| type III secretion system LEE translocated intimin receptor Tir [Escherichia coli] | 77.4 | 7e-15 | EAB6739987 |
| type III secretion system LEE translocated intimin receptor Tir [Escherichia coli] | 77.4 | 7e-15 | EAB6871963 |
| type III secretion system LEE translocated intimin receptor Tir [Escherichia coli] | 77.4 | 7e-15 | EAB7558509 |
| type III secretion system LEE translocated intimin receptor Tir [Escherichia coli] | 77.4 | 7e-15 | EAB7945944 |
| type III secretion system LEE translocated intimin receptor Tir [Escherichia coli] | 77.4 | 7e-15 | EAB8141095 |
| type III secretion system LEE translocated intimin receptor Tir [Escherichia coli] | 77.4 | 7e-15 | EAB8227922 |
| type III secretion system LEE translocated intimin receptor Tir [Escherichia coli] | 77.4 | 7e-15 | EAB9555924 |
| type III secretion system LEE translocated intimin receptor Tir [Escherichia coli] | 77.4 | 7e-15 | EAB9695030 |
| type III secretion system LEE translocated intimin receptor Tir [Escherichia coli] | 77.4 | 7e-15 | EAB9700147 |
| type III secretion system LEE translocated intimin receptor Tir [Escherichia coli] | 77.4 | 7e-15 | EAC0247279 |
| type III secretion system LEE translocated intimin receptor Tir [Escherichia coli] | 77.4 | 7e-15 | EAC1247203 |
| translocated intimin receptor Tir [Escherichia coli] | 77.4 | 7e-15 | KOZ68243 |
| type III secretion system LEE translocated intimin receptor Tir [Escherichia coli] | 77.4 | 7e-15 | MFX44795 |
| type III secretion system LEE translocated intimin receptor Tir [Escherichia coli] | 77.4 | 7e-15 | MFZ11311 |
| type III secretion system LEE translocated intimin receptor Tir [Escherichia coli] | 77.4 | 7e-15 | MJB12168 |
| type III secretion system LEE translocated intimin receptor Tir [Escherichia coli] | 77.4 | 7e-15 | MJI72184 |
| type III secretion system LEE translocated intimin receptor Tir [Escherichia coli] | 77.4 | 7e-15 | MJK89728 |
| type III secretion system LEE translocated intimin receptor Tir [Escherichia coli] | 77.4 | 7e-15 | MJM23094 |
| type III secretion system LEE translocated intimin receptor Tir [Escherichia coli] | 77.4 | 7e-15 | MJN85267 |
| type III secretion system LEE translocated intimin receptor Tir [Escherichia coli] | 77.4 | 7e-15 | MKB70978 |
| type III secretion system LEE translocated intimin receptor Tir [Escherichia coli] | 77.4 | 7e-15 | MKK43702 |
| type III secretion system LEE translocated intimin receptor Tir [Escherichia coli] | 77.4 | 7e-15 | MLA45822 |
| type III secretion system LEE translocated intimin receptor Tir [Escherichia coli] | 77.4 | 7e-15 | MME64858 |
| type III secretion system LEE translocated intimin receptor Tir [Escherichia coli] | 77.4 | 7e-15 | MMG54856 |
| type III secretion system LEE translocated intimin receptor Tir [Escherichia coli] | 77.4 | 7e-15 | MMI16889 |
| type III secretion system LEE translocated intimin receptor Tir [Escherichia coli] | 77.4 | 7e-15 | MMN00749 |
| type III secretion system LEE translocated intimin receptor Tir [Escherichia coli] | 77.4 | 7e-15 | MMQ52900 |
| type III secretion system LEE translocated intimin receptor Tir [Escherichia coli] | 77.4 | 7e-15 | POO40443 |
| type III secretion system LEE translocated intimin receptor Tir [Escherichia coli] | 77.4 | 7e-15 | TJK93584 |
| translocated intimin receptor Tir [Escherichia coli] | 77.4 | 7e-15 | STI71669 |
| type III secretion system LEE translocated intimin receptor Tir [Escherichia coli] | 77.4 | 7e-15 | WP\_053912377 |
| type III secretion system LEE translocated intimin receptor Tir [Escherichia coli] | 77.4 | 7e-15 | EAA2591134 |
| type III secretion system LEE translocated intimin receptor Tir [Escherichia coli] | 77.4 | 7e-15 | EAB1424403 |
| translocated intimin receptor Tir [Escherichia coli] | 77.4 | 7e-15 | KOZ06966 |
| translocated intimin receptor Tir [Escherichia coli] | 77.4 | 7e-15 | KOZ44291 |
| type III secretion system LEE translocated intimin receptor Tir [Escherichia coli] | 77.4 | 7e-15 | MEW34080 |
| translocated intimin receptor Tir [Escherichia coli] | 77.4 | 7e-15 | AAC69314 |
| type III secretion system LEE translocated intimin receptor Tir [Escherichia coli] | 77.4 | 7e-15 | WP\_115455646 |
| type III secretion system LEE translocated intimin receptor Tir [Escherichia coli] | 77.4 | 7e-15 | EAA2023322 |
| type III secretion system LEE translocated intimin receptor Tir [Escherichia coli] | 77.4 | 7e-15 | WP\_053893219 |
| translocated intimin receptor Tir [Escherichia coli] | 77.4 | 7e-15 | CTV81055 |
| type III secretion system LEE translocated intimin receptor Tir [Escherichia coli] | 77.4 | 7e-15 | WP\_001301454 |
| RecName: Full=Translocated intimin receptor Tir; AltName: Full=Secreted effector protein Tir [Escherichia coli] | 77.4 | 7e-15 | P0DJ92 |
| translocated intimin receptor Tir [Escherichia coli] | 77.4 | 7e-15 | AAD29391 |
| translocated intimin receptor [Escherichia coli] | 77.4 | 7e-15 | AAY25392 |
| translocated intimin receptor protein Tir [Escherichia coli] | 77.4 | 7e-15 | ACU09451 |
| translocated intimin receptor Tir [Escherichia coli] | 77.4 | 7e-15 | AMW43468 |
| translocated intimin receptor Tir [Escherichia coli] | 77.4 | 7e-15 | AMW48886 |
| translocated intimin receptor Tir [Escherichia coli] | 77.4 | 7e-15 | AOX50001 |
| translocated intimin receptor Tir [Escherichia coli] | 77.4 | 7e-15 | AOX55406 |
| translocated intimin receptor Tir [Escherichia coli] | 77.4 | 7e-15 | APA39889 |
| translocated intimin receptor Tir [Escherichia coli] | 77.4 | 7e-15 | API06369 |
| translocated intimin receptor Tir [Escherichia coli] | 77.4 | 7e-15 | API11944 |
| translocated intimin receptor Tir [Escherichia coli] | 77.4 | 7e-15 | API17508 |
| translocated intimin receptor Tir [Escherichia coli] | 77.4 | 7e-15 | API39878 |
| translocated intimin receptor Tir [Escherichia coli] | 77.4 | 7e-15 | AST63875 |
| type III secretion system LEE translocated intimin receptor Tir [Escherichia coli] | 77.4 | 7e-15 | EAA0546284 |
| type III secretion system LEE translocated intimin receptor Tir [Escherichia coli] | 77.4 | 7e-15 | EAA0729415 |
| type III secretion system LEE translocated intimin receptor Tir [Escherichia coli] | 77.4 | 7e-15 | EAA0817275 |
| type III secretion system LEE translocated intimin receptor Tir [Escherichia coli] | 77.4 | 7e-15 | EAA0871449 |
| type III secretion system LEE translocated intimin receptor Tir [Escherichia coli] | 77.4 | 7e-15 | EAA1264575 |
| type III secretion system LEE translocated intimin receptor Tir [Escherichia coli] | 77.4 | 7e-15 | EAA1318955 |
| type III secretion system LEE translocated intimin receptor Tir [Escherichia coli] | 77.4 | 7e-15 | EAA1385907 |
| type III secretion system LEE translocated intimin receptor Tir [Escherichia coli] | 77.4 | 7e-15 | EAA1646972 |
| type III secretion system LEE translocated intimin receptor Tir [Escherichia coli] | 77.4 | 7e-15 | EAA1668259 |
| type III secretion system LEE translocated intimin receptor Tir [Escherichia coli] | 77.4 | 7e-15 | EAA1848966 |
| type III secretion system LEE translocated intimin receptor Tir [Escherichia coli] | 77.4 | 7e-15 | EAA2028820 |
| type III secretion system LEE translocated intimin receptor Tir [Escherichia coli] | 77.4 | 7e-15 | EAA2162701 |
| type III secretion system LEE translocated intimin receptor Tir [Escherichia coli] | 77.4 | 7e-15 | EAA2196618 |
| type III secretion system LEE translocated intimin receptor Tir [Escherichia coli] | 77.4 | 7e-15 | EAA2281804 |
| type III secretion system LEE translocated intimin receptor Tir [Escherichia coli] | 77.4 | 7e-15 | EAA2338686 |
| type III secretion system LEE translocated intimin receptor Tir [Escherichia coli] | 77.4 | 7e-15 | EAA2483042 |
| type III secretion system LEE translocated intimin receptor Tir [Escherichia coli] | 77.4 | 7e-15 | EAA2643904 |
| type III secretion system LEE translocated intimin receptor Tir [Escherichia coli] | 77.4 | 7e-15 | EAA2767922 |
| type III secretion system LEE translocated intimin receptor Tir [Escherichia coli] | 77.4 | 7e-15 | EAA2891673 |
| type III secretion system LEE translocated intimin receptor Tir [Escherichia coli] | 77.4 | 7e-15 | EAA2911345 |
| type III secretion system LEE translocated intimin receptor Tir [Escherichia coli] | 77.4 | 7e-15 | EAA2956545 |
| type III secretion system LEE translocated intimin receptor Tir [Escherichia coli] | 77.4 | 7e-15 | EAA3145397 |
| type III secretion system LEE translocated intimin receptor Tir [Escherichia coli] | 77.4 | 7e-15 | EAA3346727 |
| type III secretion system LEE translocated intimin receptor Tir [Escherichia coli] | 77.4 | 7e-15 | EAA4600518 |
| type III secretion system LEE translocated intimin receptor Tir [Escherichia coli] | 77.4 | 7e-15 | EAA4767935 |
| type III secretion system LEE translocated intimin receptor Tir [Escherichia coli] | 77.4 | 7e-15 | EAA4784104 |
| type III secretion system LEE translocated intimin receptor Tir [Escherichia coli] | 77.4 | 7e-15 | EAA4950950 |
| type III secretion system LEE translocated intimin receptor Tir [Escherichia coli] | 77.4 | 7e-15 | EAA4971100 |
| type III secretion system LEE translocated intimin receptor Tir [Escherichia coli] | 77.4 | 7e-15 | EAA5001299 |
| type III secretion system LEE translocated intimin receptor Tir [Escherichia coli] | 77.4 | 7e-15 | EAA5073263 |
| type III secretion system LEE translocated intimin receptor Tir [Escherichia coli] | 77.4 | 7e-15 | EAA5291818 |
| type III secretion system LEE translocated intimin receptor Tir [Escherichia coli] | 77.4 | 7e-15 | EAB0429150 |
| type III secretion system LEE translocated intimin receptor Tir [Escherichia coli] | 77.4 | 7e-15 | EAB0466003 |
| type III secretion system LEE translocated intimin receptor Tir [Escherichia coli] | 77.4 | 7e-15 | EAB0469254 |
| type III secretion system LEE translocated intimin receptor Tir [Escherichia coli] | 77.4 | 7e-15 | EAB0485706 |
| type III secretion system LEE translocated intimin receptor Tir [Escherichia coli] | 77.4 | 7e-15 | EAB0504298 |
| type III secretion system LEE translocated intimin receptor Tir [Escherichia coli] | 77.4 | 7e-15 | EAB0536964 |
| type III secretion system LEE translocated intimin receptor Tir [Escherichia coli] | 77.4 | 7e-15 | EAB0563289 |
| type III secretion system LEE translocated intimin receptor Tir [Escherichia coli] | 77.4 | 7e-15 | EAB0581676 |
| type III secretion system LEE translocated intimin receptor Tir [Escherichia coli] | 77.4 | 7e-15 | EAB0609910 |
| type III secretion system LEE translocated intimin receptor Tir [Escherichia coli] | 77.4 | 7e-15 | EAB0625937 |
| type III secretion system LEE translocated intimin receptor Tir [Escherichia coli] | 77.4 | 7e-15 | EAB0629418 |
| type III secretion system LEE translocated intimin receptor Tir [Escherichia coli] | 77.4 | 7e-15 | EAB0640464 |
| type III secretion system LEE translocated intimin receptor Tir [Escherichia coli] | 77.4 | 7e-15 | EAB0647318 |
| type III secretion system LEE translocated intimin receptor Tir [Escherichia coli] | 77.4 | 7e-15 | EAB0652824 |
| type III secretion system LEE translocated intimin receptor Tir [Escherichia coli] | 77.4 | 7e-15 | EAB0685328 |
| type III secretion system LEE translocated intimin receptor Tir [Escherichia coli] | 77.4 | 7e-15 | EAB0688165 |
| type III secretion system LEE translocated intimin receptor Tir [Escherichia coli] | 77.4 | 7e-15 | EAB0700346 |
| type III secretion system LEE translocated intimin receptor Tir [Escherichia coli] | 77.4 | 7e-15 | EAB0721662 |
| type III secretion system LEE translocated intimin receptor Tir [Escherichia coli] | 77.4 | 7e-15 | EAB0761324 |
| type III secretion system LEE translocated intimin receptor Tir [Escherichia coli] | 77.4 | 7e-15 | EAB0795928 |
| type III secretion system LEE translocated intimin receptor Tir [Escherichia coli] | 77.4 | 7e-15 | EAB0808181 |
| type III secretion system LEE translocated intimin receptor Tir [Escherichia coli] | 77.4 | 7e-15 | EAB0817303 |
| type III secretion system LEE translocated intimin receptor Tir [Escherichia coli] | 77.4 | 7e-15 | EAB0843560 |
| type III secretion system LEE translocated intimin receptor Tir [Escherichia coli] | 77.4 | 7e-15 | EAB0862670 |
| type III secretion system LEE translocated intimin receptor Tir [Escherichia coli] | 77.4 | 7e-15 | EAB0867671 |
| type III secretion system LEE translocated intimin receptor Tir [Escherichia coli] | 77.4 | 7e-15 | EAB0873244 |
| type III secretion system LEE translocated intimin receptor Tir [Escherichia coli] | 77.4 | 7e-15 | EAB0910498 |
| type III secretion system LEE translocated intimin receptor Tir [Escherichia coli] | 77.4 | 7e-15 | EAB0924813 |
| type III secretion system LEE translocated intimin receptor Tir [Escherichia coli] | 77.4 | 7e-15 | EAB1011918 |
| type III secretion system LEE translocated intimin receptor Tir [Escherichia coli] | 77.4 | 7e-15 | EAB1049070 |
| type III secretion system LEE translocated intimin receptor Tir [Escherichia coli] | 77.4 | 7e-15 | EAB1060230 |
| type III secretion system LEE translocated intimin receptor Tir [Escherichia coli] | 77.4 | 7e-15 | EAB1065194 |
| type III secretion system LEE translocated intimin receptor Tir [Escherichia coli] | 77.4 | 7e-15 | EAB1142560 |
| type III secretion system LEE translocated intimin receptor Tir [Escherichia coli] | 77.4 | 7e-15 | EAB1153056 |
| type III secretion system LEE translocated intimin receptor Tir [Escherichia coli] | 77.4 | 7e-15 | EAB1156773 |
| type III secretion system LEE translocated intimin receptor Tir [Escherichia coli] | 77.4 | 7e-15 | EAB1163782 |
| type III secretion system LEE translocated intimin receptor Tir [Escherichia coli] | 77.4 | 7e-15 | EAB1174322 |
| type III secretion system LEE translocated intimin receptor Tir [Escherichia coli] | 77.4 | 7e-15 | EAB1193088 |
| type III secretion system LEE translocated intimin receptor Tir [Escherichia coli] | 77.4 | 7e-15 | EAB1244165 |
| type III secretion system LEE translocated intimin receptor Tir [Escherichia coli] | 77.4 | 7e-15 | EAB1254521 |
| type III secretion system LEE translocated intimin receptor Tir [Escherichia coli] | 77.4 | 7e-15 | EAB1271544 |
| type III secretion system LEE translocated intimin receptor Tir [Escherichia coli] | 77.4 | 7e-15 | EAB1314215 |
| type III secretion system LEE translocated intimin receptor Tir [Escherichia coli] | 77.4 | 7e-15 | EAB1330440 |
| type III secretion system LEE translocated intimin receptor Tir [Escherichia coli] | 77.4 | 7e-15 | EAB1335251 |
| type III secretion system LEE translocated intimin receptor Tir [Escherichia coli] | 77.4 | 7e-15 | EAB1380488 |
| type III secretion system LEE translocated intimin receptor Tir [Escherichia coli] | 77.4 | 7e-15 | EAB1435322 |
| type III secretion system LEE translocated intimin receptor Tir [Escherichia coli] | 77.4 | 7e-15 | EAB1444187 |
| type III secretion system LEE translocated intimin receptor Tir [Escherichia coli] | 77.4 | 7e-15 | EAB1462237 |
| type III secretion system LEE translocated intimin receptor Tir [Escherichia coli] | 77.4 | 7e-15 | EAB5532113 |
| type III secretion system LEE translocated intimin receptor Tir [Escherichia coli] | 77.4 | 7e-15 | EAB5570546 |
| type III secretion system LEE translocated intimin receptor Tir [Escherichia coli] | 77.4 | 7e-15 | EAB6006479 |
| type III secretion system LEE translocated intimin receptor Tir [Escherichia coli] | 77.4 | 7e-15 | EAB6068566 |
| type III secretion system LEE translocated intimin receptor Tir [Escherichia coli] | 77.4 | 7e-15 | EAB6140618 |
| type III secretion system LEE translocated intimin receptor Tir [Escherichia coli] | 77.4 | 7e-15 | EAB6330761 |
| type III secretion system LEE translocated intimin receptor Tir [Escherichia coli] | 77.4 | 7e-15 | EAB6734451 |
| type III secretion system LEE translocated intimin receptor Tir [Escherichia coli] | 77.4 | 7e-15 | EAB6782096 |
| type III secretion system LEE translocated intimin receptor Tir [Escherichia coli] | 77.4 | 7e-15 | EAB7173954 |
| type III secretion system LEE translocated intimin receptor Tir [Escherichia coli] | 77.4 | 7e-15 | EAB7183671 |
| type III secretion system LEE translocated intimin receptor Tir [Escherichia coli] | 77.4 | 7e-15 | EAB7212946 |
| type III secretion system LEE translocated intimin receptor Tir [Escherichia coli] | 77.4 | 7e-15 | EAB7379844 |
| type III secretion system LEE translocated intimin receptor Tir [Escherichia coli] | 77.4 | 7e-15 | EAB7851672 |
| type III secretion system LEE translocated intimin receptor Tir [Escherichia coli] | 77.4 | 7e-15 | EAB8376995 |
| type III secretion system LEE translocated intimin receptor Tir [Escherichia coli] | 77.4 | 7e-15 | EAB8652952 |
| type III secretion system LEE translocated intimin receptor Tir [Escherichia coli] | 77.4 | 7e-15 | EAB8831433 |
| type III secretion system LEE translocated intimin receptor Tir [Escherichia coli] | 77.4 | 7e-15 | EAB8887790 |
| type III secretion system LEE translocated intimin receptor Tir [Escherichia coli] | 77.4 | 7e-15 | EAB8990927 |
| type III secretion system LEE translocated intimin receptor Tir [Escherichia coli] | 77.4 | 7e-15 | EAB9191302 |
| type III secretion system LEE translocated intimin receptor Tir [Escherichia coli] | 77.4 | 7e-15 | EAB9439868 |
| type III secretion system LEE translocated intimin receptor Tir [Escherichia coli] | 77.4 | 7e-15 | EAC0106213 |
| type III secretion system LEE translocated intimin receptor Tir [Escherichia coli] | 77.4 | 7e-15 | EAC0692639 |
| type III secretion system LEE translocated intimin receptor Tir [Escherichia coli] | 77.4 | 7e-15 | EAC0713383 |
| type III secretion system LEE translocated intimin receptor Tir [Escherichia coli] | 77.4 | 7e-15 | EAC0721006 |
| type III secretion system LEE translocated intimin receptor Tir [Escherichia coli] | 77.4 | 7e-15 | EAC1170420 |
| type III secretion system LEE translocated intimin receptor Tir [Escherichia coli] | 77.4 | 7e-15 | EAC1178491 |
| type III secretion system LEE translocated intimin receptor Tir [Escherichia coli] | 77.4 | 7e-15 | EAC1284460 |
| translocated intimin receptor Tir [Escherichia coli] | 77.4 | 7e-15 | KIY30628 |
| translocated intimin receptor Tir [Escherichia coli] | 77.4 | 7e-15 | KIZ11205 |
| translocated intimin receptor Tir [Escherichia coli] | 77.4 | 7e-15 | KOZ25651 |
| translocated intimin receptor Tir [Escherichia coli] | 77.4 | 7e-15 | KOZ34152 |
| translocated intimin receptor Tir [Escherichia coli] | 77.4 | 7e-15 | KOZ48431 |
| translocated intimin receptor Tir [Escherichia coli] | 77.4 | 7e-15 | KOZ55600 |
| translocated intimin receptor Tir [Escherichia coli] | 77.4 | 7e-15 | KOZ56252 |
| translocated intimin receptor Tir [Escherichia coli] | 77.4 | 7e-15 | KOZ93790 |
| translocated intimin receptor Tir [Escherichia coli] | 77.4 | 7e-15 | KPO82465 |
| translocated intimin receptor Tir [Escherichia coli] | 77.4 | 7e-15 | KPP14863 |
| translocated intimin receptor Tir [Escherichia coli] | 77.4 | 7e-15 | KPP20028 |
| translocated intimin receptor Tir [Escherichia coli] | 77.4 | 7e-15 | KPP23353 |
| translocated intimin receptor Tir [Escherichia coli] | 77.4 | 7e-15 | KPP29464 |
| translocated intimin receptor Tir [Escherichia coli] | 77.4 | 7e-15 | KPP42670 |
| translocated intimin receptor Tir [Escherichia coli] | 77.4 | 7e-15 | KPP46308 |
| translocated intimin receptor Tir [Escherichia coli] | 77.4 | 7e-15 | KPP47532 |
| translocated intimin receptor Tir [Escherichia coli] | 77.4 | 7e-15 | KYT94909 |
| type III secretion system LEE translocated intimin receptor Tir [Escherichia coli] | 77.4 | 7e-15 | MDM08022 |
| type III secretion system LEE translocated intimin receptor Tir [Escherichia coli] | 77.4 | 7e-15 | MDO82146 |
| type III secretion system LEE translocated intimin receptor Tir [Escherichia coli] | 77.4 | 7e-15 | MDP28609 |
| type III secretion system LEE translocated intimin receptor Tir [Escherichia coli] | 77.4 | 7e-15 | MDP31845 |
| type III secretion system LEE translocated intimin receptor Tir [Escherichia coli] | 77.4 | 7e-15 | MDP85027 |
| type III secretion system LEE translocated intimin receptor Tir [Escherichia coli] | 77.4 | 7e-15 | MDQ92940 |
| type III secretion system LEE translocated intimin receptor Tir [Escherichia coli] | 77.4 | 7e-15 | MDS13711 |
| type III secretion system LEE translocated intimin receptor Tir [Escherichia coli] | 77.4 | 7e-15 | MDS18850 |
| type III secretion system LEE translocated intimin receptor Tir [Escherichia coli] | 77.4 | 7e-15 | MDT57503 |
| type III secretion system LEE translocated intimin receptor Tir [Escherichia coli] | 77.4 | 7e-15 | MDT63558 |
| type III secretion system LEE translocated intimin receptor Tir [Escherichia coli] | 77.4 | 7e-15 | MDU20152 |
| type III secretion system LEE translocated intimin receptor Tir [Escherichia coli] | 77.4 | 7e-15 | MDU37090 |
| type III secretion system LEE translocated intimin receptor Tir [Escherichia coli] | 77.4 | 7e-15 | MDU77433 |
| type III secretion system LEE translocated intimin receptor Tir [Escherichia coli] | 77.4 | 7e-15 | MDV51297 |
| type III secretion system LEE translocated intimin receptor Tir [Escherichia coli] | 77.4 | 7e-15 | MDV75174 |
| type III secretion system LEE translocated intimin receptor Tir [Escherichia coli] | 77.4 | 7e-15 | MDW01844 |
| type III secretion system LEE translocated intimin receptor Tir [Escherichia coli] | 77.4 | 7e-15 | MDW23706 |
| type III secretion system LEE translocated intimin receptor Tir [Escherichia coli] | 77.4 | 7e-15 | MDW33476 |
| type III secretion system LEE translocated intimin receptor Tir [Escherichia coli] | 77.4 | 7e-15 | MDW52214 |
| type III secretion system LEE translocated intimin receptor Tir [Escherichia coli] | 77.4 | 7e-15 | MDW71969 |
| type III secretion system LEE translocated intimin receptor Tir [Escherichia coli] | 77.4 | 7e-15 | MDW77336 |
| type III secretion system LEE translocated intimin receptor Tir [Escherichia coli] | 77.4 | 7e-15 | MDX06678 |
| type III secretion system LEE translocated intimin receptor Tir [Escherichia coli] | 77.4 | 7e-15 | MDX44681 |
| type III secretion system LEE translocated intimin receptor Tir [Escherichia coli] | 77.4 | 7e-15 | MES74310 |
| type III secretion system LEE translocated intimin receptor Tir [Escherichia coli] | 77.4 | 7e-15 | MES86924 |
| type III secretion system LEE translocated intimin receptor Tir [Escherichia coli] | 77.4 | 7e-15 | MES92075 |
| type III secretion system LEE translocated intimin receptor Tir [Escherichia coli] | 77.4 | 7e-15 | MES97660 |
| type III secretion system LEE translocated intimin receptor Tir [Escherichia coli] | 77.4 | 7e-15 | MET07301 |
| type III secretion system LEE translocated intimin receptor Tir [Escherichia coli] | 77.4 | 7e-15 | MET28737 |
| type III secretion system LEE translocated intimin receptor Tir [Escherichia coli] | 77.4 | 7e-15 | MET51844 |
| type III secretion system LEE translocated intimin receptor Tir [Escherichia coli] | 77.4 | 7e-15 | MET69522 |
| type III secretion system LEE translocated intimin receptor Tir [Escherichia coli] | 77.4 | 7e-15 | MET92645 |
| type III secretion system LEE translocated intimin receptor Tir [Escherichia coli] | 77.4 | 7e-15 | MEU06617 |
| type III secretion system LEE translocated intimin receptor Tir [Escherichia coli] | 77.4 | 7e-15 | MEV17930 |
| type III secretion system LEE translocated intimin receptor Tir [Escherichia coli] | 77.4 | 7e-15 | MEV32609 |
| type III secretion system LEE translocated intimin receptor Tir [Escherichia coli] | 77.4 | 7e-15 | MEV48466 |
| type III secretion system LEE translocated intimin receptor Tir [Escherichia coli] | 77.4 | 7e-15 | MEV69167 |
| type III secretion system LEE translocated intimin receptor Tir [Escherichia coli] | 77.4 | 7e-15 | MEV74658 |
| type III secretion system LEE translocated intimin receptor Tir [Escherichia coli] | 77.4 | 7e-15 | MEV98848 |
| type III secretion system LEE translocated intimin receptor Tir [Escherichia coli] | 77.4 | 7e-15 | MEW40491 |
| type III secretion system LEE translocated intimin receptor Tir [Escherichia coli] | 77.4 | 7e-15 | MEW72154 |
| type III secretion system LEE translocated intimin receptor Tir [Escherichia coli] | 77.4 | 7e-15 | MEW76670 |
| type III secretion system LEE translocated intimin receptor Tir [Escherichia coli] | 77.4 | 7e-15 | MEW89470 |
| type III secretion system LEE translocated intimin receptor Tir [Escherichia coli] | 77.4 | 7e-15 | MEX10149 |
| type III secretion system LEE translocated intimin receptor Tir [Escherichia coli] | 77.4 | 7e-15 | MEY11909 |
| type III secretion system LEE translocated intimin receptor Tir [Escherichia coli] | 77.4 | 7e-15 | MEZ86431 |
| type III secretion system LEE translocated intimin receptor Tir [Escherichia coli] | 77.4 | 7e-15 | MFA49603 |
| type III secretion system LEE translocated intimin receptor Tir [Escherichia coli] | 77.4 | 7e-15 | MFB92762 |
| type III secretion system LEE translocated intimin receptor Tir [Escherichia coli] | 77.4 | 7e-15 | MFB95835 |
| type III secretion system LEE translocated intimin receptor Tir [Escherichia coli] | 77.4 | 7e-15 | MFC15672 |
| type III secretion system LEE translocated intimin receptor Tir [Escherichia coli] | 77.4 | 7e-15 | MFC19133 |
| type III secretion system LEE translocated intimin receptor Tir [Escherichia coli] | 77.4 | 7e-15 | MFC30140 |
| type III secretion system LEE translocated intimin receptor Tir [Escherichia coli] | 77.4 | 7e-15 | MFC33088 |
| type III secretion system LEE translocated intimin receptor Tir [Escherichia coli] | 77.4 | 7e-15 | MFC85059 |
| type III secretion system LEE translocated intimin receptor Tir [Escherichia coli] | 77.4 | 7e-15 | MFE00075 |
| type III secretion system LEE translocated intimin receptor Tir [Escherichia coli] | 77.4 | 7e-15 | MFQ86455 |
| type III secretion system LEE translocated intimin receptor Tir [Escherichia coli] | 77.4 | 7e-15 | MFQ97516 |
| type III secretion system LEE translocated intimin receptor Tir [Escherichia coli] | 77.4 | 7e-15 | MFR12179 |
| type III secretion system LEE translocated intimin receptor Tir [Escherichia coli] | 77.4 | 7e-15 | MFR68653 |
| type III secretion system LEE translocated intimin receptor Tir [Escherichia coli] | 77.4 | 7e-15 | MFR77398 |
| type III secretion system LEE translocated intimin receptor Tir [Escherichia coli] | 77.4 | 7e-15 | MFR87915 |
| type III secretion system LEE translocated intimin receptor Tir [Escherichia coli] | 77.4 | 7e-15 | MFS07532 |
| type III secretion system LEE translocated intimin receptor Tir [Escherichia coli] | 77.4 | 7e-15 | MFT42544 |
| type III secretion system LEE translocated intimin receptor Tir [Escherichia coli] | 77.4 | 7e-15 | MFT48340 |
| type III secretion system LEE translocated intimin receptor Tir [Escherichia coli] | 77.4 | 7e-15 | MFT53714 |
| type III secretion system LEE translocated intimin receptor Tir [Escherichia coli] | 77.4 | 7e-15 | MFT57471 |
| type III secretion system LEE translocated intimin receptor Tir [Escherichia coli] | 77.4 | 7e-15 | MFT62435 |
| type III secretion system LEE translocated intimin receptor Tir [Escherichia coli] | 77.4 | 7e-15 | MFT67650 |
| type III secretion system LEE translocated intimin receptor Tir [Escherichia coli] | 77.4 | 7e-15 | MFU57456 |
| type III secretion system LEE translocated intimin receptor Tir [Escherichia coli] | 77.4 | 7e-15 | MFV56251 |
| type III secretion system LEE translocated intimin receptor Tir [Escherichia coli] | 77.4 | 7e-15 | MFW04363 |
| type III secretion system LEE translocated intimin receptor Tir [Escherichia coli] | 77.4 | 7e-15 | MFW40461 |
| type III secretion system LEE translocated intimin receptor Tir [Escherichia coli] | 77.4 | 7e-15 | MFW92034 |
| type III secretion system LEE translocated intimin receptor Tir [Escherichia coli] | 77.4 | 7e-15 | MFX56458 |
| type III secretion system LEE translocated intimin receptor Tir [Escherichia coli] | 77.4 | 7e-15 | MFX90267 |
| type III secretion system LEE translocated intimin receptor Tir [Escherichia coli] | 77.4 | 7e-15 | MFY24649 |
| type III secretion system LEE translocated intimin receptor Tir [Escherichia coli] | 77.4 | 7e-15 | MFY47555 |
| type III secretion system LEE translocated intimin receptor Tir [Escherichia coli] | 77.4 | 7e-15 | MFY60553 |
| type III secretion system LEE translocated intimin receptor Tir [Escherichia coli] | 77.4 | 7e-15 | MGC01145 |
| type III secretion system LEE translocated intimin receptor Tir [Escherichia coli] | 77.4 | 7e-15 | MGC03958 |
| type III secretion system LEE translocated intimin receptor Tir [Escherichia coli] | 77.4 | 7e-15 | MGV58420 |
| type III secretion system LEE translocated intimin receptor Tir [Escherichia coli] | 77.4 | 7e-15 | MGV82930 |
| type III secretion system LEE translocated intimin receptor Tir [Escherichia coli] | 77.4 | 7e-15 | MGX69990 |
| type III secretion system LEE translocated intimin receptor Tir [Escherichia coli] | 77.4 | 7e-15 | MHK23495 |
| type III secretion system LEE translocated intimin receptor Tir [Escherichia coli] | 77.4 | 7e-15 | MHL46755 |
| type III secretion system LEE translocated intimin receptor Tir [Escherichia coli] | 77.4 | 7e-15 | MHL60606 |
| type III secretion system LEE translocated intimin receptor Tir [Escherichia coli] | 77.4 | 7e-15 | MHL88953 |
| type III secretion system LEE translocated intimin receptor Tir [Escherichia coli] | 77.4 | 7e-15 | MIZ38302 |
| type III secretion system LEE translocated intimin receptor Tir [Escherichia coli] | 77.4 | 7e-15 | MJA00579 |
| type III secretion system LEE translocated intimin receptor Tir [Escherichia coli] | 77.4 | 7e-15 | MJA15371 |
| type III secretion system LEE translocated intimin receptor Tir [Escherichia coli] | 77.4 | 7e-15 | MJA63660 |
| type III secretion system LEE translocated intimin receptor Tir [Escherichia coli] | 77.4 | 7e-15 | MJC83531 |
| type III secretion system LEE translocated intimin receptor Tir [Escherichia coli] | 77.4 | 7e-15 | MJD06216 |
| type III secretion system LEE translocated intimin receptor Tir [Escherichia coli] | 77.4 | 7e-15 | MJD86918 |
| type III secretion system LEE translocated intimin receptor Tir [Escherichia coli] | 77.4 | 7e-15 | MJE39255 |
| type III secretion system LEE translocated intimin receptor Tir [Escherichia coli] | 77.4 | 7e-15 | MJF02736 |
| type III secretion system LEE translocated intimin receptor Tir [Escherichia coli] | 77.4 | 7e-15 | MJF36232 |
| type III secretion system LEE translocated intimin receptor Tir [Escherichia coli] | 77.4 | 7e-15 | MJH19591 |
| type III secretion system LEE translocated intimin receptor Tir [Escherichia coli] | 77.4 | 7e-15 | MJI94212 |
| type III secretion system LEE translocated intimin receptor Tir [Escherichia coli] | 77.4 | 7e-15 | MJJ47384 |
| type III secretion system LEE translocated intimin receptor Tir [Escherichia coli] | 77.4 | 7e-15 | MJN19205 |
| type III secretion system LEE translocated intimin receptor Tir [Escherichia coli] | 77.4 | 7e-15 | MJN40383 |
| type III secretion system LEE translocated intimin receptor Tir [Escherichia coli] | 77.4 | 7e-15 | MJO76667 |
| type III secretion system LEE translocated intimin receptor Tir [Escherichia coli] | 77.4 | 7e-15 | MJR58129 |
| type III secretion system LEE translocated intimin receptor Tir [Escherichia coli] | 77.4 | 7e-15 | MJR87140 |
| type III secretion system LEE translocated intimin receptor Tir [Escherichia coli] | 77.4 | 7e-15 | MKA50548 |
| type III secretion system LEE translocated intimin receptor Tir [Escherichia coli] | 77.4 | 7e-15 | MKK90677 |
| type III secretion system LEE translocated intimin receptor Tir [Escherichia coli] | 77.4 | 7e-15 | MKL01431 |
| type III secretion system LEE translocated intimin receptor Tir [Escherichia coli] | 77.4 | 7e-15 | MKL61842 |
| type III secretion system LEE translocated intimin receptor Tir [Escherichia coli] | 77.4 | 7e-15 | MKM17960 |
| type III secretion system LEE translocated intimin receptor Tir [Escherichia coli] | 77.4 | 7e-15 | MKQ79727 |
| type III secretion system LEE translocated intimin receptor Tir [Escherichia coli] | 77.4 | 7e-15 | MKR25146 |
| type III secretion system LEE translocated intimin receptor Tir [Escherichia coli] | 77.4 | 7e-15 | MKS88787 |
| type III secretion system LEE translocated intimin receptor Tir [Escherichia coli] | 77.4 | 7e-15 | MKX28781 |
| type III secretion system LEE translocated intimin receptor Tir [Escherichia coli] | 77.4 | 7e-15 | MKX55468 |
| type III secretion system LEE translocated intimin receptor Tir [Escherichia coli] | 77.4 | 7e-15 | MLA31034 |
| type III secretion system LEE translocated intimin receptor Tir [Escherichia coli] | 77.4 | 7e-15 | MLA72176 |
| type III secretion system LEE translocated intimin receptor Tir [Escherichia coli] | 77.4 | 7e-15 | MLB78398 |
| type III secretion system LEE translocated intimin receptor Tir [Escherichia coli] | 77.4 | 7e-15 | MLC40686 |
| type III secretion system LEE translocated intimin receptor Tir [Escherichia coli] | 77.4 | 7e-15 | MLE41203 |
| type III secretion system LEE translocated intimin receptor Tir [Escherichia coli] | 77.4 | 7e-15 | MLE77856 |
| type III secretion system LEE translocated intimin receptor Tir [Escherichia coli] | 77.4 | 7e-15 | MLL06575 |
| type III secretion system LEE translocated intimin receptor Tir [Escherichia coli] | 77.4 | 7e-15 | MLU30856 |
| type III secretion system LEE translocated intimin receptor Tir [Escherichia coli] | 77.4 | 7e-15 | MLY12123 |
| type III secretion system LEE translocated intimin receptor Tir [Escherichia coli] | 77.4 | 7e-15 | MLY33822 |
| type III secretion system LEE translocated intimin receptor Tir [Escherichia coli] | 77.4 | 7e-15 | MMA13947 |
| type III secretion system LEE translocated intimin receptor Tir [Escherichia coli] | 77.4 | 7e-15 | MMD83989 |
| type III secretion system LEE translocated intimin receptor Tir [Escherichia coli] | 77.4 | 7e-15 | MME83801 |
| type III secretion system LEE translocated intimin receptor Tir [Escherichia coli] | 77.4 | 7e-15 | MMF72329 |
| type III secretion system LEE translocated intimin receptor Tir [Escherichia coli] | 77.4 | 7e-15 | MMG37606 |
| type III secretion system LEE translocated intimin receptor Tir [Escherichia coli] | 77.4 | 7e-15 | MMG81888 |
| type III secretion system LEE translocated intimin receptor Tir [Escherichia coli] | 77.4 | 7e-15 | MMJ54413 |
| type III secretion system LEE translocated intimin receptor Tir [Escherichia coli] | 77.4 | 7e-15 | MML28676 |
| type III secretion system LEE translocated intimin receptor Tir [Escherichia coli] | 77.4 | 7e-15 | MML61762 |
| type III secretion system LEE translocated intimin receptor Tir [Escherichia coli] | 77.4 | 7e-15 | MMM15204 |
| type III secretion system LEE translocated intimin receptor Tir [Escherichia coli] | 77.4 | 7e-15 | MMN82612 |
| type III secretion system LEE translocated intimin receptor Tir [Escherichia coli] | 77.4 | 7e-15 | MMQ05137 |
| type III secretion system LEE translocated intimin receptor Tir [Escherichia coli] | 77.4 | 7e-15 | MMR46256 |
| type III secretion system LEE translocated intimin receptor Tir [Escherichia coli] | 77.4 | 7e-15 | MMS96912 |
| type III secretion system LEE translocated intimin receptor Tir [Escherichia coli] | 77.4 | 7e-15 | MMW10921 |
| type III secretion system LEE translocated intimin receptor Tir [Escherichia coli] | 77.4 | 7e-15 | MMW38134 |
| type III secretion system LEE translocated intimin receptor Tir [Escherichia coli] | 77.4 | 7e-15 | MMX19522 |
| type III secretion system LEE translocated intimin receptor Tir [Escherichia coli] | 77.4 | 7e-15 | MMX37288 |
| type III secretion system LEE translocated intimin receptor Tir [Escherichia coli] | 77.4 | 7e-15 | MMY54416 |
| type III secretion system LEE translocated intimin receptor Tir [Escherichia coli] | 77.4 | 7e-15 | MMY95003 |
| translocated intimin receptor Tir [Escherichia coli] | 77.4 | 7e-15 | OEG65118 |
| translocated intimin receptor Tir [Escherichia coli] | 77.4 | 7e-15 | OJF20527 |
| translocated intimin receptor Tir [Escherichia coli] | 77.4 | 7e-15 | OJF24030 |
| translocated intimin receptor Tir [Escherichia coli] | 77.4 | 7e-15 | OJF24395 |
| translocated intimin receptor Tir [Escherichia coli] | 77.4 | 7e-15 | OJF36318 |
| translocated intimin receptor Tir [Escherichia coli] | 77.4 | 7e-15 | OJF38114 |
| translocated intimin receptor Tir [Escherichia coli] | 77.4 | 7e-15 | OJF46965 |
| translocated intimin receptor Tir [Escherichia coli] | 77.4 | 7e-15 | OJF52955 |
| translocated intimin receptor Tir [Escherichia coli] | 77.4 | 7e-15 | OJF53707 |
| translocated intimin receptor Tir [Escherichia coli] | 77.4 | 7e-15 | OJF63158 |
| translocated intimin receptor Tir [Escherichia coli] | 77.4 | 7e-15 | OOC77434 |
| translocated intimin receptor Tir [Escherichia coli] | 77.4 | 7e-15 | OTB34068 |
| translocated intimin receptor Tir [Escherichia coli] | 77.4 | 7e-15 | OTB74802 |
| translocated intimin receptor Tir [Escherichia coli] | 77.4 | 7e-15 | OTC78441 |
| translocated intimin receptor Tir [Escherichia coli] | 77.4 | 7e-15 | OTC87889 |
| translocated intimin receptor Tir [Escherichia coli] | 77.4 | 7e-15 | OTC94109 |
| translocated intimin receptor Tir [Escherichia coli] | 77.4 | 7e-15 | OTD69207 |
| translocated intimin receptor Tir [Escherichia coli] | 77.4 | 7e-15 | OTE22514 |
| translocated intimin receptor Tir [Escherichia coli] | 77.4 | 7e-15 | OTE24970 |
| translocated intimin receptor Tir [Escherichia coli] | 77.4 | 7e-15 | OTE73105 |
| translocated intimin receptor Tir [Escherichia coli] | 77.4 | 7e-15 | OTV44945 |
| translocated intimin receptor Tir [Escherichia coli] | 77.4 | 7e-15 | OTV51704 |
| translocated intimin receptor Tir [Escherichia coli] | 77.4 | 7e-15 | OVA37096 |
| translocated intimin receptor Tir [Escherichia coli] | 77.4 | 7e-15 | OVA37495 |
| translocated intimin receptor Tir [Escherichia coli] | 77.4 | 7e-15 | OVA40087 |
| translocated intimin receptor Tir [Escherichia coli] | 77.4 | 7e-15 | OVA54071 |
| translocated intimin receptor Tir [Escherichia coli] | 77.4 | 7e-15 | OVA54292 |
| translocated intimin receptor Tir [Escherichia coli] | 77.4 | 7e-15 | OVA64055 |
| translocated intimin receptor Tir [Escherichia coli] | 77.4 | 7e-15 | OVA72712 |
| translocated intimin receptor Tir [Escherichia coli] | 77.4 | 7e-15 | OVA77821 |
| translocated intimin receptor Tir [Escherichia coli] | 77.4 | 7e-15 | OVA78240 |
| translocated intimin receptor Tir [Escherichia coli] | 77.4 | 7e-15 | OVA85924 |
| translocated intimin receptor Tir [Escherichia coli] | 77.4 | 7e-15 | OVA91761 |
| translocated intimin receptor Tir [Escherichia coli] | 77.4 | 7e-15 | OVA98980 |
| translocated intimin receptor Tir [Escherichia coli] | 77.4 | 7e-15 | OVB01646 |
| translocated intimin receptor Tir [Escherichia coli] | 77.4 | 7e-15 | OVB11654 |
| translocated intimin receptor Tir [Escherichia coli] | 77.4 | 7e-15 | OVB11867 |
| translocated intimin receptor Tir [Escherichia coli] | 77.4 | 7e-15 | OVB22213 |
| translocated intimin receptor Tir [Escherichia coli] | 77.4 | 7e-15 | OVB31465 |
| translocated intimin receptor Tir [Escherichia coli] | 77.4 | 7e-15 | OVB54718 |
| translocated intimin receptor Tir [Escherichia coli] | 77.4 | 7e-15 | OVB60264 |
| translocated intimin receptor Tir [Escherichia coli] | 77.4 | 7e-15 | OVB72274 |
| translocated intimin receptor Tir [Escherichia coli] | 77.4 | 7e-15 | OVB73847 |
| translocated intimin receptor Tir [Escherichia coli] | 77.4 | 7e-15 | OVB75659 |
| translocated intimin receptor Tir [Escherichia coli] | 77.4 | 7e-15 | OVB86178 |
| translocated intimin receptor Tir [Escherichia coli] | 77.4 | 7e-15 | OVB90642 |
| translocated intimin receptor Tir [Escherichia coli] | 77.4 | 7e-15 | OVB91318 |
| translocated intimin receptor Tir [Escherichia coli] | 77.4 | 7e-15 | OVC02690 |
| translocated intimin receptor Tir [Escherichia coli] | 77.4 | 7e-15 | OVC06846 |
| translocated intimin receptor Tir [Escherichia coli] | 77.4 | 7e-15 | OVC06972 |
| translocated intimin receptor Tir [Escherichia coli] | 77.4 | 7e-15 | OVC12404 |
| translocated intimin receptor Tir [Escherichia coli] | 77.4 | 7e-15 | OVC20458 |
| translocated intimin receptor Tir [Escherichia coli] | 77.4 | 7e-15 | OVC29001 |
| translocated intimin receptor Tir [Escherichia coli] | 77.4 | 7e-15 | OVC34126 |
| translocated intimin receptor Tir [Escherichia coli] | 77.4 | 7e-15 | OVC35376 |
| translocated intimin receptor Tir [Escherichia coli] | 77.4 | 7e-15 | OVC39563 |
| translocated intimin receptor Tir [Escherichia coli] | 77.4 | 7e-15 | OVC52802 |
| translocated intimin receptor Tir [Escherichia coli] | 77.4 | 7e-15 | OVC53274 |
| translocated intimin receptor Tir [Escherichia coli] | 77.4 | 7e-15 | OVC57627 |
| translocated intimin receptor Tir [Escherichia coli] | 77.4 | 7e-15 | OVC69909 |
| translocated intimin receptor Tir [Escherichia coli] | 77.4 | 7e-15 | OVC71262 |
| translocated intimin receptor Tir [Escherichia coli] | 77.4 | 7e-15 | OVC73065 |
| translocated intimin receptor Tir [Escherichia coli] | 77.4 | 7e-15 | OVC81478 |
| translocated intimin receptor Tir [Escherichia coli] | 77.4 | 7e-15 | OVC87659 |
| translocated intimin receptor Tir [Escherichia coli] | 77.4 | 7e-15 | OVC92429 |
| translocated intimin receptor Tir [Escherichia coli] | 77.4 | 7e-15 | OVD00710 |
| translocated intimin receptor Tir [Escherichia coli] | 77.4 | 7e-15 | OVD56047 |
| translocated intimin receptor Tir [Escherichia coli] | 77.4 | 7e-15 | OVD67167 |
| translocated intimin receptor Tir [Escherichia coli] | 77.4 | 7e-15 | OVD73148 |
| translocated intimin receptor Tir [Escherichia coli] | 77.4 | 7e-15 | OVD74380 |
| translocated intimin receptor Tir [Escherichia coli] | 77.4 | 7e-15 | OVE21207 |
| translocated intimin receptor Tir [Escherichia coli] | 77.4 | 7e-15 | OVE24385 |
| translocated intimin receptor Tir [Escherichia coli] | 77.4 | 7e-15 | OVE26846 |
| translocated intimin receptor Tir [Escherichia coli] | 77.4 | 7e-15 | PAU04979 |
| type III secretion system LEE translocated intimin receptor Tir [Escherichia coli] | 77.4 | 7e-15 | PDV06085 |
| type III secretion system LEE translocated intimin receptor Tir [Escherichia coli] | 77.4 | 7e-15 | PDV15138 |
| type III secretion system LEE translocated intimin receptor Tir [Escherichia coli] | 77.4 | 7e-15 | PDV32395 |
| type III secretion system LEE translocated intimin receptor Tir [Escherichia coli] | 77.4 | 7e-15 | PDV38823 |
| type III secretion system LEE translocated intimin receptor Tir [Escherichia coli] | 77.4 | 7e-15 | PDV62905 |
| type III secretion system LEE translocated intimin receptor Tir [Escherichia coli] | 77.4 | 7e-15 | PDV63541 |
| type III secretion system LEE translocated intimin receptor Tir [Escherichia coli] | 77.4 | 7e-15 | PEH63758 |
| type III secretion system LEE translocated intimin receptor Tir [Escherichia coli] | 77.4 | 7e-15 | PEH94317 |
| type III secretion system LEE translocated intimin receptor Tir [Escherichia coli] | 77.4 | 7e-15 | PEI19106 |
| type III secretion system LEE translocated intimin receptor Tir [Escherichia coli] | 77.4 | 7e-15 | PJG20414 |
| type III secretion system LEE translocated intimin receptor Tir [Escherichia coli] | 77.4 | 7e-15 | PJG30671 |
| type III secretion system LEE translocated intimin receptor Tir [Escherichia coli] | 77.4 | 7e-15 | PSX47916 |
| type III secretion system LEE translocated intimin receptor Tir [Escherichia coli] | 77.4 | 7e-15 | PSX56757 |
| type III secretion system LEE translocated intimin receptor Tir [Escherichia coli] | 77.4 | 7e-15 | PSX64811 |
| type III secretion system LEE translocated intimin receptor Tir [Escherichia coli] | 77.4 | 7e-15 | PSX64883 |
| type III secretion system LEE translocated intimin receptor Tir [Escherichia coli] | 77.4 | 7e-15 | PSX75522 |
| type III secretion system LEE translocated intimin receptor Tir [Escherichia coli] | 77.4 | 7e-15 | PSX80676 |
| type III secretion system LEE translocated intimin receptor Tir [Escherichia coli] | 77.4 | 7e-15 | PSX85177 |
| type III secretion system LEE translocated intimin receptor Tir [Escherichia coli] | 77.4 | 7e-15 | PSX86822 |
| type III secretion system LEE translocated intimin receptor Tir [Escherichia coli] | 77.4 | 7e-15 | PSX94871 |
| type III secretion system LEE translocated intimin receptor Tir [Escherichia coli] | 77.4 | 7e-15 | PSY00392 |
| type III secretion system LEE translocated intimin receptor Tir [Escherichia coli] | 77.4 | 7e-15 | PSY02007 |
| type III secretion system LEE translocated intimin receptor Tir [Escherichia coli] | 77.4 | 7e-15 | PSY09296 |
| type III secretion system LEE translocated intimin receptor Tir [Escherichia coli] | 77.4 | 7e-15 | PSY23673 |
| type III secretion system LEE translocated intimin receptor Tir [Escherichia coli] | 77.4 | 7e-15 | QCL23871 |
| type III secretion system LEE translocated intimin receptor Tir [Escherichia coli] | 77.4 | 7e-15 | QCL29304 |
| type III secretion system LEE translocated intimin receptor Tir [Escherichia coli] | 77.4 | 7e-15 | QCL34737 |
| type III secretion system LEE translocated intimin receptor Tir [Escherichia coli] | 77.4 | 7e-15 | QCL45818 |
| type III secretion system LEE translocated intimin receptor Tir [Escherichia coli] | 77.4 | 7e-15 | QCL51484 |
| type III secretion system LEE translocated intimin receptor Tir [Escherichia coli] | 77.4 | 7e-15 | QCM69393 |
| type III secretion system LEE translocated intimin receptor Tir [Escherichia coli] | 77.4 | 7e-15 | QCM74867 |
| type III secretion system LEE translocated intimin receptor Tir [Escherichia coli] | 77.4 | 7e-15 | QCM80340 |
| type III secretion system LEE translocated intimin receptor Tir [Escherichia coli] | 77.4 | 7e-15 | QCN35133 |
| type III secretion system LEE translocated intimin receptor Tir [Escherichia coli] | 77.4 | 7e-15 | QCN40684 |
| type III secretion system LEE translocated intimin receptor Tir [Escherichia coli] | 77.4 | 7e-15 | QCN46233 |
| type III secretion system LEE translocated intimin receptor Tir [Escherichia coli] | 77.4 | 7e-15 | QCN51707 |
| type III secretion system LEE translocated intimin receptor Tir [Escherichia coli] | 77.4 | 7e-15 | QCN57182 |
| type III secretion system LEE translocated intimin receptor Tir [Escherichia coli] | 77.4 | 7e-15 | QCN62665 |
| type III secretion system LEE translocated intimin receptor Tir [Escherichia coli] | 77.4 | 7e-15 | QCN68142 |
| type III secretion system LEE translocated intimin receptor Tir [Escherichia coli] | 77.4 | 7e-15 | QCN73595 |
| type III secretion system LEE translocated intimin receptor Tir [Escherichia coli] | 77.4 | 7e-15 | RDH58702 |
| type III secretion system LEE translocated intimin receptor Tir [Escherichia coli] | 77.4 | 7e-15 | RFQ84565 |
| type III secretion system LEE translocated intimin receptor Tir [Escherichia coli] | 77.4 | 7e-15 | TGF04693 |
| type III secretion system LEE translocated intimin receptor Tir [Escherichia coli] | 77.4 | 7e-15 | TGF10883 |
| type III secretion system LEE translocated intimin receptor Tir [Escherichia coli] | 77.4 | 7e-15 | TGF12051 |
| type III secretion system LEE translocated intimin receptor Tir [Escherichia coli] | 77.4 | 7e-15 | TGF19865 |
| type III secretion system LEE translocated intimin receptor Tir [Escherichia coli] | 77.4 | 7e-15 | TGF27080 |
| type III secretion system LEE translocated intimin receptor Tir [Escherichia coli] | 77.4 | 7e-15 | TGF27236 |
| type III secretion system LEE translocated intimin receptor Tir [Escherichia coli] | 77.4 | 7e-15 | TGF36289 |
| type III secretion system LEE translocated intimin receptor Tir [Escherichia coli] | 77.4 | 7e-15 | TGF41619 |
| type III secretion system LEE translocated intimin receptor Tir [Escherichia coli] | 77.4 | 7e-15 | TJC43781 |
| type III secretion system LEE translocated intimin receptor Tir [Escherichia coli] | 77.4 | 7e-15 | TJC65990 |
| type III secretion system LEE translocated intimin receptor Tir [Escherichia coli] | 77.4 | 7e-15 | TJC74039 |
| type III secretion system LEE translocated intimin receptor Tir [Escherichia coli] | 77.4 | 7e-15 | TJC80420 |
| type III secretion system LEE translocated intimin receptor Tir [Escherichia coli] | 77.4 | 7e-15 | TJC88567 |
| type III secretion system LEE translocated intimin receptor Tir [Escherichia coli] | 77.4 | 7e-15 | TJE01803 |
| type III secretion system LEE translocated intimin receptor Tir [Escherichia coli] | 77.4 | 7e-15 | TJE57115 |
| type III secretion system LEE translocated intimin receptor Tir [Escherichia coli] | 77.4 | 7e-15 | TJE96594 |
| type III secretion system LEE translocated intimin receptor Tir [Escherichia coli] | 77.4 | 7e-15 | TJF52290 |
| type III secretion system LEE translocated intimin receptor Tir [Escherichia coli] | 77.4 | 7e-15 | TJF66002 |
| type III secretion system LEE translocated intimin receptor Tir [Escherichia coli] | 77.4 | 7e-15 | TJF66235 |
| type III secretion system LEE translocated intimin receptor Tir [Escherichia coli] | 77.4 | 7e-15 | TJF95617 |
| type III secretion system LEE translocated intimin receptor Tir [Escherichia coli] | 77.4 | 7e-15 | TJG01395 |
| type III secretion system LEE translocated intimin receptor Tir [Escherichia coli] | 77.4 | 7e-15 | TJG32558 |
| type III secretion system LEE translocated intimin receptor Tir [Escherichia coli] | 77.4 | 7e-15 | TJG57603 |
| type III secretion system LEE translocated intimin receptor Tir [Escherichia coli] | 77.4 | 7e-15 | TJG70412 |
| type III secretion system LEE translocated intimin receptor Tir [Escherichia coli] | 77.4 | 7e-15 | TJH57638 |
| type III secretion system LEE translocated intimin receptor Tir [Escherichia coli] | 77.4 | 7e-15 | TJH87760 |
| type III secretion system LEE translocated intimin receptor Tir [Escherichia coli] | 77.4 | 7e-15 | TKT15197 |
| type III secretion system LEE translocated intimin receptor Tir [Escherichia coli] | 77.4 | 7e-15 | TKT18797 |
| type III secretion system LEE translocated intimin receptor Tir [Escherichia coli] | 77.4 | 7e-15 | TKT23343 |
| type III secretion system LEE translocated intimin receptor Tir [Escherichia coli] | 77.4 | 7e-15 | TKT36234 |
| type III secretion system LEE translocated intimin receptor Tir [Escherichia coli] | 77.4 | 7e-15 | TKT52193 |
| type III secretion system LEE translocated intimin receptor Tir [Escherichia coli] | 77.4 | 7e-15 | TKT62642 |
| T3SS translocated intimin receptor Tir [Escherichia coli] | 77.4 | 7e-15 | GCH51304 |
| type III secretion system LEE translocated intimin receptor Tir [Escherichia coli] | 77.4 | 7e-15 | WP\_136145139 |
| type III secretion system LEE translocated intimin receptor Tir [Escherichia coli] | 77.4 | 7e-15 | WP\_001359655 |
| translocated intimin receptor [Escherichia coli] | 77.4 | 7e-15 | AAY25390 |
| translocated intimin receptor [Escherichia coli] | 77.4 | 7e-15 | AAY25391 |
| translocated intimin receptor Tir [Escherichia coli] | 77.4 | 7e-15 | KNG38771 |
| type III secretion system LEE translocated intimin receptor Tir [Escherichia coli] | 77.4 | 7e-15 | MDP71350 |
| translocated intimin receptor Tir [Escherichia coli] | 77.4 | 7e-15 | OWC16444 |
| type III secretion system LEE translocated intimin receptor Tir [Escherichia coli] | 77.4 | 7e-15 | RCP40748 |
| translocated intimin receptor Tir [Escherichia coli] | 77.4 | 7e-15 | CTS02837 |
| intimin receptor protein [Escherichia coli] | 77.4 | 7e-15 | ADK73987 |
| type III secretion system LEE translocated intimin receptor Tir [Escherichia coli] | 77.4 | 7e-15 | WP\_021497278 |
| type III secretion system LEE translocated intimin receptor Tir [Escherichia coli] | 77.4 | 7e-15 | WP\_107154545 |
| type III secretion system LEE translocated intimin receptor Tir [Escherichia coli] | 77.4 | 7e-15 | PSY14502 |
| type III secretion system LEE translocated intimin receptor Tir [Escherichia coli] | 77.4 | 7e-15 | WP\_053919485 |
| translocated intimin receptor Tir [Escherichia coli] | 77.4 | 7e-15 | CUA38865 |
| type III secretion system LEE translocated intimin receptor Tir [Escherichia coli] | 77.4 | 7e-15 | WP\_001359418 |
| translocated intimin receptor Tir [Escherichia coli] | 77.4 | 7e-15 | ANE64693 |
| translocated intimin receptor Tir [Escherichia coli] | 77.4 | 7e-15 | API00751 |
| translocated intimin receptor Tir [Escherichia coli] | 77.4 | 7e-15 | API34317 |
| type III secretion system LEE translocated intimin receptor Tir [Escherichia coli] | 77.4 | 7e-15 | EAA0784888 |
| type III secretion system LEE translocated intimin receptor Tir [Escherichia coli] | 77.4 | 7e-15 | EAA0796934 |
| type III secretion system LEE translocated intimin receptor Tir [Escherichia coli] | 77.4 | 7e-15 | EAA1173946 |
| type III secretion system LEE translocated intimin receptor Tir [Escherichia coli] | 77.4 | 7e-15 | EAA1418060 |
| type III secretion system LEE translocated intimin receptor Tir [Escherichia coli] | 77.4 | 7e-15 | EAA1567780 |
| type III secretion system LEE translocated intimin receptor Tir [Escherichia coli] | 77.4 | 7e-15 | EAA1876882 |
| type III secretion system LEE translocated intimin receptor Tir [Escherichia coli] | 77.4 | 7e-15 | EAA2311713 |
| type III secretion system LEE translocated intimin receptor Tir [Escherichia coli] | 77.4 | 7e-15 | EAA2382784 |
| type III secretion system LEE translocated intimin receptor Tir [Escherichia coli] | 77.4 | 7e-15 | EAA3158143 |
| type III secretion system LEE translocated intimin receptor Tir [Escherichia coli] | 77.4 | 7e-15 | EAA3170195 |
| type III secretion system LEE translocated intimin receptor Tir [Escherichia coli] | 77.4 | 7e-15 | EAA3266476 |
| type III secretion system LEE translocated intimin receptor Tir [Escherichia coli] | 77.4 | 7e-15 | EAA3495616 |
| type III secretion system LEE translocated intimin receptor Tir [Escherichia coli] | 77.4 | 7e-15 | EAA3531237 |
| type III secretion system LEE translocated intimin receptor Tir [Escherichia coli] | 77.4 | 7e-15 | EAA4676403 |
| type III secretion system LEE translocated intimin receptor Tir [Escherichia coli] | 77.4 | 7e-15 | EAA4773340 |
| type III secretion system LEE translocated intimin receptor Tir [Escherichia coli] | 77.4 | 7e-15 | EAA4781438 |
| type III secretion system LEE translocated intimin receptor Tir [Escherichia coli] | 77.4 | 7e-15 | EAA4832923 |
| type III secretion system LEE translocated intimin receptor Tir [Escherichia coli] | 77.4 | 7e-15 | EAA4985799 |
| type III secretion system LEE translocated intimin receptor Tir [Escherichia coli] | 77.4 | 7e-15 | EAB0500635 |
| type III secretion system LEE translocated intimin receptor Tir [Escherichia coli] | 77.4 | 7e-15 | EAB6808351 |
| type III secretion system LEE translocated intimin receptor Tir [Escherichia coli] | 77.4 | 7e-15 | EAB7255787 |
| type III secretion system LEE translocated intimin receptor Tir [Escherichia coli] | 77.4 | 7e-15 | EAB7478557 |
| type III secretion system LEE translocated intimin receptor Tir [Escherichia coli] | 77.4 | 7e-15 | EAB7875383 |
| type III secretion system LEE translocated intimin receptor Tir [Escherichia coli] | 77.4 | 7e-15 | EAB8105843 |
| type III secretion system LEE translocated intimin receptor Tir [Escherichia coli] | 77.4 | 7e-15 | EAB8981277 |
| type III secretion system LEE translocated intimin receptor Tir [Escherichia coli] | 77.4 | 7e-15 | EAB9181140 |
| type III secretion system LEE translocated intimin receptor Tir [Escherichia coli] | 77.4 | 7e-15 | EAB9270422 |
| type III secretion system LEE translocated intimin receptor Tir [Escherichia coli] | 77.4 | 7e-15 | EAB9405928 |
| type III secretion system LEE translocated intimin receptor Tir [Escherichia coli] | 77.4 | 7e-15 | EAB9414435 |
| type III secretion system LEE translocated intimin receptor Tir [Escherichia coli] | 77.4 | 7e-15 | EAB9671572 |
| type III secretion system LEE translocated intimin receptor Tir [Escherichia coli] | 77.4 | 7e-15 | EAC0214478 |
| type III secretion system LEE translocated intimin receptor Tir [Escherichia coli] | 77.4 | 7e-15 | EAC0439408 |
| type III secretion system LEE translocated intimin receptor Tir [Escherichia coli] | 77.4 | 7e-15 | EAC0736011 |
| type III secretion system LEE translocated intimin receptor Tir [Escherichia coli] | 77.4 | 7e-15 | EAC0743334 |
| type III secretion system LEE translocated intimin receptor Tir [Escherichia coli] | 77.4 | 7e-15 | EAC1302834 |
| translocated intimin receptor Tir [Escherichia coli] | 77.4 | 7e-15 | KOZ10503 |
| translocated intimin receptor Tir [Escherichia coli] | 77.4 | 7e-15 | KPH33876 |
| type III secretion system LEE translocated intimin receptor Tir [Escherichia coli] | 77.4 | 7e-15 | MDL65497 |
| type III secretion system LEE translocated intimin receptor Tir [Escherichia coli] | 77.4 | 7e-15 | MDL78174 |
| type III secretion system LEE translocated intimin receptor Tir [Escherichia coli] | 77.4 | 7e-15 | MDX24369 |
| type III secretion system LEE translocated intimin receptor Tir [Escherichia coli] | 77.4 | 7e-15 | MIT60410 |
| type III secretion system LEE translocated intimin receptor Tir [Escherichia coli] | 77.4 | 7e-15 | MIZ88508 |
| type III secretion system LEE translocated intimin receptor Tir [Escherichia coli] | 77.4 | 7e-15 | MJF67284 |
| type III secretion system LEE translocated intimin receptor Tir [Escherichia coli] | 77.4 | 7e-15 | MJH24694 |
| type III secretion system LEE translocated intimin receptor Tir [Escherichia coli] | 77.4 | 7e-15 | MJK12797 |
| type III secretion system LEE translocated intimin receptor Tir [Escherichia coli] | 77.4 | 7e-15 | MJK28675 |
| type III secretion system LEE translocated intimin receptor Tir [Escherichia coli] | 77.4 | 7e-15 | MJK83841 |
| type III secretion system LEE translocated intimin receptor Tir [Escherichia coli] | 77.4 | 7e-15 | MJQ59598 |
| type III secretion system LEE translocated intimin receptor Tir [Escherichia coli] | 77.4 | 7e-15 | MJR34110 |
| type III secretion system LEE translocated intimin receptor Tir [Escherichia coli] | 77.4 | 7e-15 | MJR54319 |
| type III secretion system LEE translocated intimin receptor Tir [Escherichia coli] | 77.4 | 7e-15 | MJX81190 |
| type III secretion system LEE translocated intimin receptor Tir [Escherichia coli] | 77.4 | 7e-15 | MKB18079 |
| type III secretion system LEE translocated intimin receptor Tir [Escherichia coli] | 77.4 | 7e-15 | MKD21740 |
| type III secretion system LEE translocated intimin receptor Tir [Escherichia coli] | 77.4 | 7e-15 | MKF81505 |
| type III secretion system LEE translocated intimin receptor Tir [Escherichia coli] | 77.4 | 7e-15 | MKV97427 |
| type III secretion system LEE translocated intimin receptor Tir [Escherichia coli] | 77.4 | 7e-15 | MLI71401 |
| type III secretion system LEE translocated intimin receptor Tir [Escherichia coli] | 77.4 | 7e-15 | MLK08234 |
| type III secretion system LEE translocated intimin receptor Tir [Escherichia coli] | 77.4 | 7e-15 | MLK80095 |
| type III secretion system LEE translocated intimin receptor Tir [Escherichia coli] | 77.4 | 7e-15 | MLL72176 |
| type III secretion system LEE translocated intimin receptor Tir [Escherichia coli] | 77.4 | 7e-15 | MLY65862 |
| type III secretion system LEE translocated intimin receptor Tir [Escherichia coli] | 77.4 | 7e-15 | MMN32330 |
| type III secretion system LEE translocated intimin receptor Tir [Escherichia coli] | 77.4 | 7e-15 | MMV78325 |
| translocated intimin receptor Tir [Escherichia coli] | 77.4 | 7e-15 | OTD78008 |
| type III secretion system LEE translocated intimin receptor Tir [Escherichia coli] | 77.4 | 7e-15 | RZN92583 |
| type III secretion system LEE translocated intimin receptor Tir [Escherichia coli] | 77.4 | 7e-15 | TJR87013 |
| translocated intimin receptor Tir [Escherichia coli] | 77.4 | 7e-15 | SQN17067 |
| translocated intimin receptor Tir [Escherichia coli] | 77.4 | 7e-15 | STK11012 |
| translocated intimin receptor protein Tir [Escherichia coli] | 77.4 | 7e-15 | ACG59681 |
| type III secretion system LEE translocated intimin receptor Tir [Escherichia coli] | 77.4 | 7e-15 | MJI06784 |
| translocated intimin receptor [Escherichia coli] | 77.4 | 7e-15 | AVZ66232 |
| type III secretion system LEE translocated intimin receptor Tir [Escherichia coli] | 77.4 | 7e-15 | MFA55483 |
| type III secretion system LEE translocated intimin receptor Tir [Escherichia coli] | 77.4 | 7e-15 | WP\_096167951 |
| type III secretion system LEE translocated intimin receptor Tir [Escherichia coli] | 77.4 | 7e-15 | WP\_137525667 |
| T3SS translocated intimin receptor Tir [Escherichia coli] | 77.4 | 7e-15 | GDG95579 |
| type III secretion system LEE translocated intimin receptor Tir [Escherichia coli] | 77.4 | 7e-15 | WP\_053913357 |
| translocated intimin receptor Tir [Escherichia coli] | 77.4 | 7e-15 | KOZ38081 |
| type III secretion system LEE translocated intimin receptor Tir [Escherichia coli] | 77.4 | 7e-15 | WP\_078165107 |
| translocated intimin receptor Tir [Escherichia coli] | 77.4 | 7e-15 | OON51781 |
| type III secretion system LEE translocated intimin receptor Tir [Escherichia coli] | 77.4 | 7e-15 | WP\_089563420 |
| type III secretion system LEE translocated intimin receptor Tir [Escherichia coli] | 77.4 | 7e-15 | MEZ70898 |
| translocated intimin receptor Tir [Escherichia coli] | 77.4 | 7e-15 | OXL48398 |
| type III secretion system LEE translocated intimin receptor Tir [Escherichia coli] | 77.4 | 7e-15 | MFD23574 |
| type III secretion system LEE translocated intimin receptor Tir [Escherichia coli] | 77.4 | 7e-15 | MHK42813 |
| type III secretion system LEE translocated intimin receptor Tir [Escherichia coli] | 77.4 | 7e-15 | MML49070 |
| type III secretion system LEE translocated intimin receptor Tir [Escherichia coli] | 77.4 | 7e-15 | MFZ21848 |
| type III secretion system LEE translocated intimin receptor Tir [Escherichia coli] | 77.4 | 7e-15 | MFZ32475 |
| type III secretion system LEE translocated intimin receptor Tir [Escherichia coli] | 77.4 | 7e-15 | EAB8167729 |
| type III secretion system LEE translocated intimin receptor Tir [Escherichia coli] | 77.4 | 7e-15 | MFY97119 |
| type III secretion system LEE translocated intimin receptor Tir [Escherichia coli] | 77.4 | 7e-15 | WP\_054194120 |
| translocated intimin receptor Tir [Escherichia coli] | 77.4 | 7e-15 | KPH49271 |
| type III secretion system LEE translocated intimin receptor Tir [Escherichia coli] | 77.4 | 7e-15 | WP\_001455714 |
| type III secretion system LEE translocated intimin receptor Tir [Escherichia coli] | 77.4 | 7e-15 | MLZ18758 |
| type III secretion system LEE translocated intimin receptor Tir [Escherichia coli] | 77.4 | 7e-15 | WP\_136145334 |
| type III secretion system LEE translocated intimin receptor Tir [Escherichia coli] | 77.4 | 7e-15 | WP\_100084875 |
| translocated intimin receptor protein [Escherichia coli] | 77.4 | 7e-15 | ASL56831 |
| translocated intimin receptor Tir [Escherichia coli] | 77.4 | 7e-15 | STI62682 |
| type III secretion system LEE translocated intimin receptor Tir [Escherichia coli] | 77.4 | 7e-15 | MFC55688 |
| type III secretion system LEE translocated intimin receptor Tir [Escherichia coli] | 77.4 | 7e-15 | MDV13763 |
| type III secretion system LEE translocated intimin receptor Tir, partial [Escherichia coli] | 77.4 | 7e-15 | WP\_113560709 |
| type III secretion system LEE translocated intimin receptor Tir, partial [Escherichia coli] | 77.4 | 7e-15 | WP\_064771187 |
| type III secretion system LEE translocated intimin receptor Tir, partial [Escherichia coli] | 77.4 | 7e-15 | WP\_032168716 |
| type III secretion system LEE translocated intimin receptor Tir, partial [Escherichia coli] | 77.4 | 7e-15 | WP\_064771229 |
| type III secretion system LEE translocated intimin receptor Tir [Escherichia coli] | 77.4 | 7e-15 | EAA2987554 |
| type III secretion system LEE translocated intimin receptor Tir, partial [Escherichia coli] | 77.4 | 7e-15 | WP\_032310298 |
| type III secretion system LEE translocated intimin receptor Tir, partial [Escherichia coli] | 77.4 | 7e-15 | WP\_064769689 |
| type III secretion system LEE translocated intimin receptor Tir [Escherichia coli] | 77.4 | 7e-15 | EAA2878035 |
| type III secretion system LEE translocated intimin receptor Tir, partial [Escherichia coli] | 77.4 | 7e-15 | WP\_105495921 |
| type III secretion system LEE translocated intimin receptor Tir, partial [Escherichia coli] | 77.4 | 7e-15 | WP\_062897189 |
| translocated intimin receptor Tir, partial [Escherichia coli] | 77.4 | 7e-15 | KYR77083 |
| type III secretion system LEE translocated intimin receptor Tir, partial [Escherichia coli] | 77.4 | 7e-15 | WP\_096937792 |
| type III secretion system LEE translocated intimin receptor Tir, partial [Escherichia coli] | 77.4 | 7e-15 | WP\_087676104 |
| translocated intimin receptor Tir, partial [Escherichia coli] | 77.4 | 7e-15 | OVD05201 |
| type III secretion system LEE translocated intimin receptor Tir [Escherichia coli] | 73.6 | 1e-13 | WP\_097735753 |
| type III secretion system LEE translocated intimin receptor Tir [Escherichia coli] | 72.3 | 4e-13 | WP\_024219034 |
| translocated intimin receptor Tir [Escherichia coli] | 72.3 | 4e-13 | CTT81489 |
| type III secretion system LEE translocated intimin receptor Tir [Escherichia coli] | 72.3 | 4e-13 | WP\_052935730 |
| type III secretion system LEE translocated intimin receptor Tir [Escherichia coli] | 72.3 | 4e-13 | PCS94128 |
| translocated intimin receptor Tir [Escherichia coli] | 72.3 | 4e-13 | CTR92821 |
| translocated intimin receptor Tir [Escherichia coli] | 72.3 | 4e-13 | CTT16297 |
| translocated intimin receptor Tir [Escherichia coli] | 72.3 | 4e-13 | CTU18965 |
| translocated intimin receptor Tir [Escherichia coli] | 72.3 | 4e-13 | STJ90130 |
| type III secretion system LEE translocated intimin receptor Tir [Escherichia coli] | 72.3 | 4e-13 | WP\_053881923 |
| type III secretion system LEE translocated intimin receptor Tir [Escherichia coli] | 72.3 | 4e-13 | EAA2293198 |
| translocated intimin receptor Tir [Escherichia coli] | 72.3 | 4e-13 | CTV06133 |
| Escherichia coli 6-175-07\_S1\_C1 [enterobacteria]  Next Previous First | | | |
| tir [Escherichia coli 6-175-07\_S1\_C1] | 77.4 | 7e-15 | KEL52623 |
| Escherichia coli T1282\_01 [enterobacteria]  Next Previous First | | | |
| tir [Escherichia coli T1282\_01] | 77.4 | 7e-15 | ERE15302 |
| Escherichia coli 99.1775 [enterobacteria]  Next Previous First | | | |
| tir [Escherichia coli 99.1775] | 77.4 | 7e-15 | ELV49521 |
| Escherichia coli 99.1781 [enterobacteria]  Next Previous First | | | |
| tir [Escherichia coli 99.1781] | 77.4 | 7e-15 | ELW11313 |
| Escherichia coli 96.0939 [enterobacteria]  Next Previous First | | | |
| tir [Escherichia coli 96.0939] | 77.4 | 7e-15 | EKW44965 |
| Escherichia coli O157:H7 str. EC869 [enterobacteria]  Next Previous First | | | |
| Tir [Escherichia coli O157:H7 str. EC869] | 77.4 | 7e-15 | EDU93042 |
| Escherichia coli FRIK1996 [enterobacteria]  Next Previous First | | | |
| putative translocated intimin receptor protein [Escherichia coli FRIK1996] | 77.4 | 7e-15 | EIN17398 |
| Escherichia coli FRIK1990 [enterobacteria]  Next Previous First | | | |
| putative translocated intimin receptor protein [Escherichia coli FRIK1990] | 77.4 | 7e-15 | EIN37967 |
| Escherichia coli PA40 [enterobacteria]  Next Previous First | | | |
| putative translocated intimin receptor protein [Escherichia coli PA40] | 77.4 | 7e-15 | EIO25477 |
| Escherichia coli PA41 [enterobacteria]  Next Previous First | | | |
| putative translocated intimin receptor protein [Escherichia coli PA41] | 77.4 | 7e-15 | EIO32767 |
| Escherichia coli TW09195 [enterobacteria]  Next Previous First | | | |
| putative translocated intimin receptor protein [Escherichia coli TW09195] | 77.4 | 7e-15 | EIO89408 |
| Escherichia coli FRIK920 [enterobacteria]  Next Previous First | | | |
| putative translocated intimin receptor protein [Escherichia coli FRIK920] | 77.4 | 7e-15 | EKG96487 |
| Escherichia coli FRIK1999 [enterobacteria]  Next Previous First | | | |
| putative translocated intimin receptor protein [Escherichia coli FRIK1999] | 77.4 | 7e-15 | EKH27656 |
| Escherichia coli NE1487 [enterobacteria]  Next Previous First | | | |
| putative translocated intimin receptor protein [Escherichia coli NE1487] | 77.4 | 7e-15 | EKH37994 |
| Escherichia coli FRIK2001 [enterobacteria]  Next Previous First | | | |
| putative translocated intimin receptor protein [Escherichia coli FRIK2001] | 77.4 | 7e-15 | EKH49985 |
| Escherichia coli MA6 [enterobacteria]  Next Previous First | | | |
| putative translocated intimin receptor protein [Escherichia coli MA6] | 77.4 | 7e-15 | EKH85424 |
| Escherichia coli 90.0039 [enterobacteria]  Next Previous First | | | |
| tir [Escherichia coli 90.0039] | 77.4 | 7e-15 | EKV93650 |
| Escherichia coli 95.0183 [enterobacteria]  Next Previous First | | | |
| tir [Escherichia coli 95.0183] | 77.4 | 7e-15 | EKW22648 |
| Escherichia coli 99.0672 [enterobacteria]  Next Previous First | | | |
| tir [Escherichia coli 99.0672] | 77.4 | 7e-15 | EKW78156 |
| Escherichia coli ATCC 700728 [enterobacteria]  Next Previous First | | | |
| tir [Escherichia coli ATCC 700728] | 77.4 | 7e-15 | ELV64891 |
| Escherichia coli 08BKT055439 [enterobacteria]  Next Previous First | | | |
| tir [Escherichia coli 08BKT055439] | 77.4 | 7e-15 | ERC71842 |
| Escherichia coli T234\_00 [enterobacteria]  Next Previous First | | | |
| tir [Escherichia coli T234\_00] | 77.4 | 7e-15 | ERC75644 |
| Escherichia coli 14A [enterobacteria]  Next Previous First | | | |
| tir [Escherichia coli 14A] | 77.4 | 7e-15 | ERC79241 |
| Escherichia coli 08BKT77219 [enterobacteria]  Next Previous First | | | |
| tir [Escherichia coli 08BKT77219] | 77.4 | 7e-15 | ERE01392 |
| Escherichia coli O157:H7 str. 2009C-4258 [enterobacteria]  Next Previous First | | | |
| translocated intimin receptor Tir [Escherichia coli O157:H7 str. 2009C-4258] | 77.4 | 7e-15 | EZE60362 |
| Escherichia coli O55:H7 str. RM12579 [enterobacteria]  Next Previous First | | | |
| translocated intimin receptor Tir [Escherichia coli O55:H7 str. RM12579] | 77.4 | 7e-15 | AEZ42789 |
| Escherichia coli DEC5C [enterobacteria]  Next Previous First | | | |
| putative translocated intimin receptor protein [Escherichia coli DEC5C] | 77.4 | 7e-15 | EHV33451 |
| Escherichia coli DEC5D [enterobacteria]  Next Previous First | | | |
| putative translocated intimin receptor protein [Escherichia coli DEC5D] | 77.4 | 7e-15 | EHV33911 |
| Escherichia coli O55:H7 str. TB182A [enterobacteria]  Next Previous First | | | |
| translocated intimin receptor Tir [Escherichia coli O55:H7 str. TB182A] | 77.4 | 7e-15 | PJR38933 |
| Escherichia coli 99.0814 [enterobacteria]  Next Previous First | | | |
| tir [Escherichia coli 99.0814] | 77.4 | 7e-15 | ELV15361 |
| Escherichia coli 99.0815 [enterobacteria]  Next Previous First | | | |
| tir [Escherichia coli 99.0815] | 77.4 | 7e-15 | ELV24451 |
| Escherichia coli 99.0816 [enterobacteria]  Next Previous First | | | |
| tir [Escherichia coli 99.0816] | 77.4 | 7e-15 | ELV32412 |
| Escherichia coli 95.0943 [enterobacteria]  Next Previous First | | | |
| tir [Escherichia coli 95.0943] | 77.4 | 7e-15 | EKW24224 |
| Escherichia coli 99.1753 [enterobacteria]  Next Previous First | | | |
| tir [Escherichia coli 99.1753] | 77.4 | 7e-15 | ELV45934 |
| Escherichia coli B107 [enterobacteria]  Next Previous First | | | |
| tir [Escherichia coli B107] | 77.4 | 7e-15 | ERB68619 |
| Escherichia coli B102 [enterobacteria]  Next Previous First | | | |
| tir [Escherichia coli B102] | 77.4 | 7e-15 | ERB69210 |
| Escherichia coli B26-1 [enterobacteria]  Next Previous First | | | |
| tir [Escherichia coli B26-1] | 77.4 | 7e-15 | ERB82141 |
| Escherichia coli B26-2 [enterobacteria]  Next Previous First | | | |
| tir [Escherichia coli B26-2] | 77.4 | 7e-15 | ERB86277 |
| Escherichia coli B103 [enterobacteria]  Next Previous First | | | |
| tir [Escherichia coli B103] | 77.4 | 7e-15 | ERC95532 |
| Escherichia coli B104 [enterobacteria]  Next Previous First | | | |
| tir [Escherichia coli B104] | 77.4 | 7e-15 | ERC95799 |
| Escherichia coli B105 [enterobacteria]  Next Previous First | | | |
| tir [Escherichia coli B105] | 77.4 | 7e-15 | ERD07114 |
| Escherichia coli B106 [enterobacteria]  Next Previous First | | | |
| tir [Escherichia coli B106] | 77.4 | 7e-15 | ERD11158 |
| Escherichia coli B40-2 [enterobacteria]  Next Previous First | | | |
| tir [Escherichia coli B40-2] | 77.4 | 7e-15 | ERD56582 |
| Escherichia coli B40-1 [enterobacteria]  Next Previous First | | | |
| tir [Escherichia coli B40-1] | 77.4 | 7e-15 | ERD57890 |
| Escherichia coli B49-2 [enterobacteria]  Next Previous First | | | |
| tir [Escherichia coli B49-2] | 77.4 | 7e-15 | ERD60716 |
| Escherichia coli B83 [enterobacteria]  Next Previous First | | | |
| tir [Escherichia coli B83] | 77.4 | 7e-15 | ERD74563 |
| Escherichia coli B84 [enterobacteria]  Next Previous First | | | |
| tir [Escherichia coli B84] | 77.4 | 7e-15 | ERD77967 |
| Escherichia coli B85 [enterobacteria]  Next Previous First | | | |
| tir [Escherichia coli B85] | 77.4 | 7e-15 | ERD85177 |
| Escherichia coli B86 [enterobacteria]  Next Previous First | | | |
| tir [Escherichia coli B86] | 77.4 | 7e-15 | ERD89422 |
| Escherichia coli O157:H7 str. F7350 [enterobacteria]  Next Previous First | | | |
| translocated intimin receptor Tir [Escherichia coli O157:H7 str. F7350] | 77.4 | 7e-15 | EYV94022 |
| Escherichia coli O157:H7 str. F7377 [enterobacteria]  Next Previous First | | | |
| translocated intimin receptor Tir [Escherichia coli O157:H7 str. F7377] | 77.4 | 7e-15 | EZB17380 |
| Escherichia coli O145:NM str. 2010C-4557C2 [enterobacteria]  Next Previous First | | | |
| translocated intimin receptor Tir [Escherichia coli O145:NM str. 2010C-4557C2] | 77.4 | 7e-15 | EYZ14778 |
| synthetic construct [other sequences]  Next Previous First | | | |
| EspA/intimin/Tir fusion protein [synthetic construct] | 77.4 | 7e-15 | ACS74757 |
| EspA/intimin/Tir fusion protein [synthetic construct] | 77.4 | 7e-15 | ACN62547 |
| Escherichia coli O145:H28 str. RM13514 [enterobacteria]  Next Previous First | | | |
| translocated intimin receptor Tir [Escherichia coli O145:H28 str. RM13514] | 77.4 | 7e-15 | AHG11345 |
| Escherichia coli O145:H28 str. RM13516 [enterobacteria]  Next Previous First | | | |
| translocated intimin receptor Tir [Escherichia coli O145:H28 str. RM13516] | 77.4 | 7e-15 | AHG17093 |
| Escherichia coli O145:H28 str. RM12761 [enterobacteria]  Next Previous First | | | |
| translocated intimin receptor Tir [Escherichia coli O145:H28 str. RM12761] | 77.4 | 7e-15 | AHY67416 |
| Escherichia coli O145:H28 str. RM12581 [enterobacteria]  Next Previous First | | | |
| translocated intimin receptor Tir [Escherichia coli O145:H28 str. RM12581] | 77.4 | 7e-15 | AHY73167 |
| Escherichia coli O145 str. RM9872 [enterobacteria]  Next Previous First | | | |
| type III secretion system LEE translocated intimin receptor Tir [Escherichia coli O145 str. RM9872] | 77.4 | 7e-15 | AWN80160 |
| Escherichia coli O145 [enterobacteria]  Next Previous First | | | |
| type III secretion system LEE translocated intimin receptor Tir [Escherichia coli O145] | 77.4 | 7e-15 | EAB0801437 |
| Escherichia coli O145:NM str. 2010C-3526 [enterobacteria]  Next Previous First | | | |
| translocated intimin receptor Tir [Escherichia coli O145:NM str. 2010C-3526] | 77.4 | 7e-15 | EYV08938 |
| Escherichia coli O145:NM str. 2010C-3521 [enterobacteria]  Next Previous First | | | |
| translocated intimin receptor Tir [Escherichia coli O145:NM str. 2010C-3521] | 77.4 | 7e-15 | EYV20308 |
| Escherichia coli O145:NM str. 2010C-3517 [enterobacteria]  Next Previous First | | | |
| translocated intimin receptor Tir [Escherichia coli O145:NM str. 2010C-3517] | 77.4 | 7e-15 | EYV26859 |
| Escherichia coli O145:NM str. 2010C-3518 [enterobacteria]  Next Previous First | | | |
| translocated intimin receptor Tir [Escherichia coli O145:NM str. 2010C-3518] | 77.4 | 7e-15 | EYV28813 |
| Escherichia coli O145:NM str. 2010C-3516 [enterobacteria]  Next Previous First | | | |
| translocated intimin receptor Tir [Escherichia coli O145:NM str. 2010C-3516] | 77.4 | 7e-15 | EYV31582 |
| Escherichia coli O145:NM str. 2010C-3510 [enterobacteria]  Next Previous First | | | |
| translocated intimin receptor Tir [Escherichia coli O145:NM str. 2010C-3510] | 77.4 | 7e-15 | EYV36024 |
| Escherichia coli O145:NM str. 2010C-3509 [enterobacteria]  Next Previous First | | | |
| translocated intimin receptor Tir [Escherichia coli O145:NM str. 2010C-3509] | 77.4 | 7e-15 | EYV38638 |
| Escherichia coli O145:NM str. 2010C-3511 [enterobacteria]  Next Previous First | | | |
| translocated intimin receptor Tir [Escherichia coli O145:NM str. 2010C-3511] | 77.4 | 7e-15 | EYV45256 |
| Escherichia coli O145:NM str. 2010C-3507 [enterobacteria]  Next Previous First | | | |
| translocated intimin receptor Tir [Escherichia coli O145:NM str. 2010C-3507] | 77.4 | 7e-15 | EYV56469 |
| Escherichia coli O145:NM str. 08-4270 [enterobacteria]  Next Previous First | | | |
| translocated intimin receptor Tir [Escherichia coli O145:NM str. 08-4270] | 77.4 | 7e-15 | EYW94186 |
| Escherichia coli O145:NM str. 06-3484 [enterobacteria]  Next Previous First | | | |
| translocated intimin receptor Tir [Escherichia coli O145:NM str. 06-3484] | 77.4 | 7e-15 | EYZ72793 |
| Escherichia coli O145:NM str. 2010C-3508 [enterobacteria]  Next Previous First | | | |
| translocated intimin receptor Tir [Escherichia coli O145:NM str. 2010C-3508] | 77.4 | 7e-15 | EZE93267 |
| Escherichia coli O145:H28 str. 4865/96 [enterobacteria]  Next Previous First | | | |
| translocated intimin receptor Tir [Escherichia coli O145:H28 str. 4865/96] | 77.4 | 7e-15 | KDM76967 |
| Escherichia coli O145:NM [enterobacteria]  Next Previous First | | | |
| type III secretion system LEE translocated intimin receptor Tir [Escherichia coli O145:NM] | 77.4 | 7e-15 | QCH91477 |
| Shigella boydii [enterobacteria]  Next Previous First | | | |
| type III secretion system LEE translocated intimin receptor Tir [Shigella boydii] | 77.4 | 7e-15 | WP\_075330760 |
| translocated intimin receptor Tir [Shigella boydii] | 77.4 | 7e-15 | OLM40674 |
| translocated intimin receptor Tir [Shigella boydii] | 77.4 | 7e-15 | OOO78464 |
| Shigella dysenteriae [enterobacteria]  Next Previous First | | | |
| type III secretion system LEE translocated intimin receptor Tir [Shigella dysenteriae] | 77.4 | 7e-15 | MLU14253 |
| Escherichia coli O157:H- str. 493-89 [enterobacteria]  Next Previous First | | | |
| Translocated intimin receptor Tir [Escherichia coli O157:H- str. 493-89] | 77.4 | 7e-15 | EFX13832 |
| Escherichia coli O157:H- str. H 2687 [enterobacteria]  Next Previous First | | | |
| Translocated intimin receptor Tir [Escherichia coli O157:H- str. H 2687] | 77.4 | 7e-15 | EFX18556 |
| Escherichia coli DEC3F [enterobacteria]  Next Previous First | | | |
| putative translocated intimin receptor protein [Escherichia coli DEC3F] | 77.4 | 7e-15 | EHU81637 |
| Escherichia coli TW06591 [enterobacteria]  Next Previous First | | | |
| putative translocated intimin receptor protein [Escherichia coli TW06591] | 77.4 | 7e-15 | EIO47265 |
| Escherichia coli 5412 [enterobacteria]  Next Previous First | | | |
| putative translocated intimin receptor protein [Escherichia coli 5412] | 77.4 | 7e-15 | EKI07127 |
| Escherichia coli TW10119 [enterobacteria]  Next Previous First | | | |
| putative translocated intimin receptor protein [Escherichia coli TW10119] | 77.4 | 7e-15 | EIO80429 |
| Escherichia coli 90.0091 [enterobacteria]  Next Previous First | | | |
| tir [Escherichia coli 90.0091] | 77.4 | 7e-15 | EKV87175 |
| Escherichia coli 99.1805 [enterobacteria]  Next Previous First | | | |
| tir [Escherichia coli 99.1805] | 77.4 | 7e-15 | ELV64471 |
| Escherichia coli O157:NM str. 08-4540 [enterobacteria]  Next Previous First | | | |
| translocated intimin receptor Tir [Escherichia coli O157:NM str. 08-4540] | 77.4 | 7e-15 | EZD87604 |
| Escherichia coli O157:H7 str. Sakai [enterobacteria]  Next Previous First | | | |
| T3SS translocated intimin receptor Tir [Escherichia coli O157:H7 str. Sakai] | 77.4 | 7e-15 | NP\_312588 |
| T3SS translocated intimin receptor Tir [Escherichia coli O157:H7 str. Sakai] | 77.4 | 7e-15 | BAB37984 |
| Escherichia coli O157:H7 str. EC4115 [enterobacteria]  Next Previous First | | | |
| RecName: Full=Translocated intimin receptor Tir; AltName: Full=Secreted effector protein Tir [Escherichia coli O157:H7 str. EC4115] | 77.4 | 7e-15 | B5YWI0 |
| Tir [Escherichia coli O157:H7 str. EC4115] | 77.4 | 7e-15 | ACI36828 |
| Escherichia coli O157:H7 str. TW14359 [enterobacteria]  Next Previous First | | | |
| RecName: Full=Translocated intimin receptor Tir; AltName: Full=Secreted effector protein Tir [Escherichia coli O157:H7 str. TW14359] | 77.4 | 7e-15 | C6UYL8 |
| translocated intimin receptor protein [Escherichia coli O157:H7 str. TW14359] | 77.4 | 7e-15 | ACT74397 |
| Escherichia coli Xuzhou21 [enterobacteria]  Next Previous First | | | |
| hypothetical protein CDCO157\_4295 [Escherichia coli Xuzhou21] | 77.4 | 7e-15 | AFJ31351 |
| Escherichia coli O157:H7 str. SS17 [enterobacteria]  Next Previous First | | | |
| translocated intimin receptor Tir [Escherichia coli O157:H7 str. SS17] | 77.4 | 7e-15 | AIF96252 |
| Escherichia coli O157:H7 str. SS52 [enterobacteria]  Next Previous First | | | |
| translocated intimin receptor Tir [Escherichia coli O157:H7 str. SS52] | 77.4 | 7e-15 | AJA28725 |
| Escherichia coli O157:H7 str. EC4196 [enterobacteria]  Next Previous First | | | |
| translocated intimin receptor Tir [Escherichia coli O157:H7 str. EC4196] | 77.4 | 7e-15 | EDU35566 |
| Escherichia coli O157:H7 str. EC4113 [enterobacteria]  Next Previous First | | | |
| translocated intimin receptor Tir [Escherichia coli O157:H7 str. EC4113] | 77.4 | 7e-15 | EDU55117 |
| Escherichia coli O157:H7 str. EC4076 [enterobacteria]  Next Previous First | | | |
| translocated intimin receptor Tir [Escherichia coli O157:H7 str. EC4076] | 77.4 | 7e-15 | EDU71612 |
| Escherichia coli O157:H7 str. EC4401 [enterobacteria]  Next Previous First | | | |
| translocated intimin receptor Tir [Escherichia coli O157:H7 str. EC4401] | 77.4 | 7e-15 | EDU77751 |
| Escherichia coli O157:H7 str. EC4486 [enterobacteria]  Next Previous First | | | |
| translocated intimin receptor Tir [Escherichia coli O157:H7 str. EC4486] | 77.4 | 7e-15 | EDU83183 |
| Escherichia coli O157:H7 str. EC4501 [enterobacteria]  Next Previous First | | | |
| translocated intimin receptor Tir [Escherichia coli O157:H7 str. EC4501] | 77.4 | 7e-15 | EDU88285 |
| Escherichia coli O157:H7 str. EC508 [enterobacteria]  Next Previous First | | | |
| translocated intimin receptor Tir [Escherichia coli O157:H7 str. EC508] | 77.4 | 7e-15 | EDU97151 |
| Escherichia coli O157:H7 str. EC4206 [enterobacteria]  Next Previous First | | | |
| translocated intimin receptor Tir [Escherichia coli O157:H7 str. EC4206] | 77.4 | 7e-15 | EDZ75188 |
| Escherichia coli O157:H7 str. EC4045 [enterobacteria]  Next Previous First | | | |
| translocated intimin receptor Tir [Escherichia coli O157:H7 str. EC4045] | 77.4 | 7e-15 | EDZ83362 |
| Escherichia coli O157:H7 str. EC4042 [enterobacteria]  Next Previous First | | | |
| translocated intimin receptor Tir [Escherichia coli O157:H7 str. EC4042] | 77.4 | 7e-15 | EDZ88496 |
| Escherichia coli O157:H7 str. TW14588 [enterobacteria]  Next Previous First | | | |
| Tir [Escherichia coli O157:H7 str. TW14588] | 77.4 | 7e-15 | EEC29355 |
| Escherichia coli O157:H7 str. EC1212 [enterobacteria]  Next Previous First | | | |
| translocated intimin receptor Tir [Escherichia coli O157:H7 str. EC1212] | 77.4 | 7e-15 | EFW65913 |
| Escherichia coli O157:H7 str. G5101 [enterobacteria]  Next Previous First | | | |
| translocated intimin receptor protein [Escherichia coli O157:H7 str. G5101] | 77.4 | 7e-15 | EFX08967 |
| Escherichia coli O157:H7 str. LSU-61 [enterobacteria]  Next Previous First | | | |
| translocated intimin receptor protein [Escherichia coli O157:H7 str. LSU-61] | 77.4 | 7e-15 | EFX33165 |
| Escherichia coli O157:H7 str. 1044 [enterobacteria]  Next Previous First | | | |
| translocated intimin receptor Tir [Escherichia coli O157:H7 str. 1044] | 77.4 | 7e-15 | EGD61150 |
| Escherichia coli O157:H7 str. 1125 [enterobacteria]  Next Previous First | | | |
| translocated intimin receptor Tir [Escherichia coli O157:H7 str. 1125] | 77.4 | 7e-15 | EGD65478 |
| Escherichia coli DEC3A [enterobacteria]  Next Previous First | | | |
| putative translocated intimin receptor protein [Escherichia coli DEC3A] | 77.4 | 7e-15 | EHU53896 |
| Escherichia coli DEC3B [enterobacteria]  Next Previous First | | | |
| putative translocated intimin receptor protein [Escherichia coli DEC3B] | 77.4 | 7e-15 | EHU54348 |
| Escherichia coli DEC3C [enterobacteria]  Next Previous First | | | |
| putative translocated intimin receptor protein [Escherichia coli DEC3C] | 77.4 | 7e-15 | EHU66979 |
| Escherichia coli DEC4E [enterobacteria]  Next Previous First | | | |
| putative translocated intimin receptor protein [Escherichia coli DEC4E] | 77.4 | 7e-15 | EHV07924 |
| Escherichia coli DEC4F [enterobacteria]  Next Previous First | | | |
| putative translocated intimin receptor protein [Escherichia coli DEC4F] | 77.4 | 7e-15 | EHV18309 |
| Escherichia coli FDA505 [enterobacteria]  Next Previous First | | | |
| putative translocated intimin receptor protein [Escherichia coli FDA505] | 77.4 | 7e-15 | EIN18211 |
| Escherichia coli FDA517 [enterobacteria]  Next Previous First | | | |
| putative translocated intimin receptor protein [Escherichia coli FDA517] | 77.4 | 7e-15 | EIN18642 |
| Escherichia coli FRIK1985 [enterobacteria]  Next Previous First | | | |
| putative translocated intimin receptor protein [Escherichia coli FRIK1985] | 77.4 | 7e-15 | EIN34562 |
| Escherichia coli 93-001 [enterobacteria]  Next Previous First | | | |
| putative translocated intimin receptor protein [Escherichia coli 93-001] | 77.4 | 7e-15 | EIN34813 |
| Escherichia coli PA3 [enterobacteria]  Next Previous First | | | |
| putative translocated intimin receptor protein [Escherichia coli PA3] | 77.4 | 7e-15 | EIN50711 |
| Escherichia coli PA5 [enterobacteria]  Next Previous First | | | |
| putative translocated intimin receptor protein [Escherichia coli PA5] | 77.4 | 7e-15 | EIN53677 |
| Escherichia coli PA9 [enterobacteria]  Next Previous First | | | |
| putative translocated intimin receptor protein [Escherichia coli PA9] | 77.4 | 7e-15 | EIN57067 |
| Escherichia coli PA10 [enterobacteria]  Next Previous First | | | |
| putative translocated intimin receptor protein [Escherichia coli PA10] | 77.4 | 7e-15 | EIN67274 |
| Escherichia coli PA14 [enterobacteria]  Next Previous First | | | |
| putative translocated intimin receptor protein [Escherichia coli PA14] | 77.4 | 7e-15 | EIN71092 |
| Escherichia coli PA15 [enterobacteria]  Next Previous First | | | |
| putative translocated intimin receptor protein [Escherichia coli PA15] | 77.4 | 7e-15 | EIN72189 |
| Escherichia coli PA25 [enterobacteria]  Next Previous First | | | |
| putative translocated intimin receptor protein [Escherichia coli PA25] | 77.4 | 7e-15 | EIN91364 |
| Escherichia coli PA24 [enterobacteria]  Next Previous First | | | |
| putative translocated intimin receptor protein [Escherichia coli PA24] | 77.4 | 7e-15 | EIN91696 |
| Escherichia coli PA28 [enterobacteria]  Next Previous First | | | |
| putative translocated intimin receptor protein [Escherichia coli PA28] | 77.4 | 7e-15 | EIN97067 |
| Escherichia coli PA31 [enterobacteria]  Next Previous First | | | |
| putative translocated intimin receptor protein [Escherichia coli PA31] | 77.4 | 7e-15 | EIO09086 |
| Escherichia coli PA32 [enterobacteria]  Next Previous First | | | |
| putative translocated intimin receptor protein [Escherichia coli PA32] | 77.4 | 7e-15 | EIO09609 |
| Escherichia coli PA33 [enterobacteria]  Next Previous First | | | |
| putative translocated intimin receptor protein [Escherichia coli PA33] | 77.4 | 7e-15 | EIO12931 |
| Escherichia coli PA39 [enterobacteria]  Next Previous First | | | |
| putative translocated intimin receptor protein [Escherichia coli PA39] | 77.4 | 7e-15 | EIO29373 |
| Escherichia coli PA42 [enterobacteria]  Next Previous First | | | |
| putative translocated intimin receptor protein [Escherichia coli PA42] | 77.4 | 7e-15 | EIO34333 |
| Escherichia coli TW07945 [enterobacteria]  Next Previous First | | | |
| putative translocated intimin receptor protein [Escherichia coli TW07945] | 77.4 | 7e-15 | EIO53742 |
| Escherichia coli TW09098 [enterobacteria]  Next Previous First | | | |
| putative translocated intimin receptor protein [Escherichia coli TW09098] | 77.4 | 7e-15 | EIO67907 |
| Escherichia coli TW09109 [enterobacteria]  Next Previous First | | | |
| putative translocated intimin receptor protein [Escherichia coli TW09109] | 77.4 | 7e-15 | EIO72048 |
| Escherichia coli EC4203 [enterobacteria]  Next Previous First | | | |
| putative translocated intimin receptor protein [Escherichia coli EC4203] | 77.4 | 7e-15 | EIO88961 |
| Escherichia coli EC4196 [enterobacteria]  Next Previous First | | | |
| putative translocated intimin receptor protein [Escherichia coli EC4196] | 77.4 | 7e-15 | EIO93598 |
| Escherichia coli O157:H7 str. TW14313 [enterobacteria]  Next Previous First | | | |
| putative translocated intimin receptor protein [Escherichia coli O157:H7 str. TW14313] | 77.4 | 7e-15 | EIP05332 |
| translocated intimin receptor Tir [Escherichia coli O157:H7 str. TW14313] | 77.4 | 7e-15 | PJR33086 |
| Escherichia coli TW14301 [enterobacteria]  Next Previous First | | | |
| putative translocated intimin receptor protein [Escherichia coli TW14301] | 77.4 | 7e-15 | EIP07023 |
| Escherichia coli EC4421 [enterobacteria]  Next Previous First | | | |
| putative translocated intimin receptor protein [Escherichia coli EC4421] | 77.4 | 7e-15 | EIP11559 |
| Escherichia coli EC4422 [enterobacteria]  Next Previous First | | | |
| putative translocated intimin receptor protein [Escherichia coli EC4422] | 77.4 | 7e-15 | EIP20865 |
| Escherichia coli EC4013 [enterobacteria]  Next Previous First | | | |
| putative translocated intimin receptor protein [Escherichia coli EC4013] | 77.4 | 7e-15 | EIP24894 |
| Escherichia coli EC4402 [enterobacteria]  Next Previous First | | | |
| putative translocated intimin receptor protein [Escherichia coli EC4402] | 77.4 | 7e-15 | EIP28874 |
| Escherichia coli EC4439 [enterobacteria]  Next Previous First | | | |
| putative translocated intimin receptor protein [Escherichia coli EC4439] | 77.4 | 7e-15 | EIP36426 |
| Escherichia coli EC4436 [enterobacteria]  Next Previous First | | | |
| putative translocated intimin receptor protein [Escherichia coli EC4436] | 77.4 | 7e-15 | EIP41619 |
| Escherichia coli EC4437 [enterobacteria]  Next Previous First | | | |
| putative translocated intimin receptor protein [Escherichia coli EC4437] | 77.4 | 7e-15 | EIP50188 |
| Escherichia coli EC4448 [enterobacteria]  Next Previous First | | | |
| putative translocated intimin receptor protein [Escherichia coli EC4448] | 77.4 | 7e-15 | EIP51654 |
| Escherichia coli EC1738 [enterobacteria]  Next Previous First | | | |
| putative translocated intimin receptor protein [Escherichia coli EC1738] | 77.4 | 7e-15 | EIP57278 |
| Escherichia coli EC1734 [enterobacteria]  Next Previous First | | | |
| putative translocated intimin receptor protein [Escherichia coli EC1734] | 77.4 | 7e-15 | EIP64884 |
| Escherichia coli EC1863 [enterobacteria]  Next Previous First | | | |
| putative translocated intimin receptor protein [Escherichia coli EC1863] | 77.4 | 7e-15 | EIP74482 |
| Escherichia coli EC1845 [enterobacteria]  Next Previous First | | | |
| putative translocated intimin receptor protein [Escherichia coli EC1845] | 77.4 | 7e-15 | EIP74892 |
| Escherichia coli PA7 [enterobacteria]  Next Previous First | | | |
| putative translocated intimin receptor protein [Escherichia coli PA7] | 77.4 | 7e-15 | EKG96004 |
| Escherichia coli PA34 [enterobacteria]  Next Previous First | | | |
| putative translocated intimin receptor protein [Escherichia coli PA34] | 77.4 | 7e-15 | EKH00492 |
| Escherichia coli FDA506 [enterobacteria]  Next Previous First | | | |
| putative translocated intimin receptor protein [Escherichia coli FDA506] | 77.4 | 7e-15 | EKH10090 |
| Escherichia coli FDA507 [enterobacteria]  Next Previous First | | | |
| putative translocated intimin receptor protein [Escherichia coli FDA507] | 77.4 | 7e-15 | EKH14410 |
| Escherichia coli FDA504 [enterobacteria]  Next Previous First | | | |
| putative translocated intimin receptor protein [Escherichia coli FDA504] | 77.4 | 7e-15 | EKH21836 |
| Escherichia coli NE037 [enterobacteria]  Next Previous First | | | |
| putative translocated intimin receptor protein [Escherichia coli NE037] | 77.4 | 7e-15 | EKH44241 |
| Escherichia coli PA4 [enterobacteria]  Next Previous First | | | |
| intimin receptor protein [Escherichia coli PA4] | 77.4 | 7e-15 | EKH55786 |
| Escherichia coli PA23 [enterobacteria]  Next Previous First | | | |
| putative translocated intimin receptor protein [Escherichia coli PA23] | 77.4 | 7e-15 | EKH64653 |
| Escherichia coli PA49 [enterobacteria]  Next Previous First | | | |
| putative translocated intimin receptor protein [Escherichia coli PA49] | 77.4 | 7e-15 | EKH66825 |
| Escherichia coli PA45 [enterobacteria]  Next Previous First | | | |
| putative translocated intimin receptor protein [Escherichia coli PA45] | 77.4 | 7e-15 | EKH73091 |
| Escherichia coli TT12B [enterobacteria]  Next Previous First | | | |
| putative translocated intimin receptor protein [Escherichia coli TT12B] | 77.4 | 7e-15 | EKH80704 |
| Escherichia coli CB7326 [enterobacteria]  Next Previous First | | | |
| putative translocated intimin receptor protein [Escherichia coli CB7326] | 77.4 | 7e-15 | EKH97783 |
| Escherichia coli EC96038 [enterobacteria]  Next Previous First | | | |
| putative translocated intimin receptor protein [Escherichia coli EC96038] | 77.4 | 7e-15 | EKI04360 |
| Escherichia coli PA38 [enterobacteria]  Next Previous First | | | |
| putative translocated intimin receptor protein [Escherichia coli PA38] | 77.4 | 7e-15 | EKI37706 |
| Escherichia coli EC1736 [enterobacteria]  Next Previous First | | | |
| putative translocated intimin receptor protein [Escherichia coli EC1736] | 77.4 | 7e-15 | EKI58329 |
| Escherichia coli EC1846 [enterobacteria]  Next Previous First | | | |
| putative translocated intimin receptor protein [Escherichia coli EC1846] | 77.4 | 7e-15 | EKI66301 |
| Escherichia coli EC1847 [enterobacteria]  Next Previous First | | | |
| putative translocated intimin receptor protein [Escherichia coli EC1847] | 77.4 | 7e-15 | EKI74024 |
| Escherichia coli EC1848 [enterobacteria]  Next Previous First | | | |
| putative translocated intimin receptor protein [Escherichia coli EC1848] | 77.4 | 7e-15 | EKI77646 |
| Escherichia coli EC1849 [enterobacteria]  Next Previous First | | | |
| putative translocated intimin receptor protein [Escherichia coli EC1849] | 77.4 | 7e-15 | EKI83953 |
| Escherichia coli EC1850 [enterobacteria]  Next Previous First | | | |
| putative translocated intimin receptor protein [Escherichia coli EC1850] | 77.4 | 7e-15 | EKI91432 |
| Escherichia coli EC1856 [enterobacteria]  Next Previous First | | | |
| putative translocated intimin receptor protein [Escherichia coli EC1856] | 77.4 | 7e-15 | EKI94303 |
| Escherichia coli EC1862 [enterobacteria]  Next Previous First | | | |
| putative translocated intimin receptor protein [Escherichia coli EC1862] | 77.4 | 7e-15 | EKJ02120 |
| Escherichia coli EC1864 [enterobacteria]  Next Previous First | | | |
| putative translocated intimin receptor protein [Escherichia coli EC1864] | 77.4 | 7e-15 | EKJ07436 |
| Escherichia coli EC1868 [enterobacteria]  Next Previous First | | | |
| putative translocated intimin receptor protein [Escherichia coli EC1868] | 77.4 | 7e-15 | EKJ21277 |
| Escherichia coli EC1866 [enterobacteria]  Next Previous First | | | |
| putative translocated intimin receptor protein [Escherichia coli EC1866] | 77.4 | 7e-15 | EKJ22066 |
| Escherichia coli EC1869 [enterobacteria]  Next Previous First | | | |
| putative translocated intimin receptor protein [Escherichia coli EC1869] | 77.4 | 7e-15 | EKJ32408 |
| Escherichia coli EC1870 [enterobacteria]  Next Previous First | | | |
| putative translocated intimin receptor protein [Escherichia coli EC1870] | 77.4 | 7e-15 | EKJ37408 |
| Escherichia coli NE098 [enterobacteria]  Next Previous First | | | |
| putative translocated intimin receptor protein [Escherichia coli NE098] | 77.4 | 7e-15 | EKJ39260 |
| Escherichia coli FRIK523 [enterobacteria]  Next Previous First | | | |
| putative translocated intimin receptor protein [Escherichia coli FRIK523] | 77.4 | 7e-15 | EKJ48995 |
| Escherichia coli 0.1304 [enterobacteria]  Next Previous First | | | |
| putative translocated intimin receptor protein [Escherichia coli 0.1304] | 77.4 | 7e-15 | EKJ56669 |
| Escherichia coli 5.2239 [enterobacteria]  Next Previous First | | | |
| tir [Escherichia coli 5.2239] | 77.4 | 7e-15 | EKK22702 |
| Escherichia coli 3.4870 [enterobacteria]  Next Previous First | | | |
| tir [Escherichia coli 3.4870] | 77.4 | 7e-15 | EKK23172 |
| Escherichia coli 6.0172 [enterobacteria]  Next Previous First | | | |
| intimin receptor protein [Escherichia coli 6.0172] | 77.4 | 7e-15 | EKK23740 |
| Escherichia coli 8.0586 [enterobacteria]  Next Previous First | | | |
| tir [Escherichia coli 8.0586] | 77.4 | 7e-15 | EKK40111 |
| Escherichia coli 10.0833 [enterobacteria]  Next Previous First | | | |
| intimin receptor protein [Escherichia coli 10.0833] | 77.4 | 7e-15 | EKK51564 |
| Escherichia coli 8.2524 [enterobacteria]  Next Previous First | | | |
| tir [Escherichia coli 8.2524] | 77.4 | 7e-15 | EKK54359 |
| Escherichia coli 10.0869 [enterobacteria]  Next Previous First | | | |
| tir [Escherichia coli 10.0869] | 77.4 | 7e-15 | EKK63353 |
| Escherichia coli 10.0821 [enterobacteria]  Next Previous First | | | |
| tir [Escherichia coli 10.0821] | 77.4 | 7e-15 | EKK82742 |
| Escherichia coli 88.1042 [enterobacteria]  Next Previous First | | | |
| tir [Escherichia coli 88.1042] | 77.4 | 7e-15 | EKV72011 |
| Escherichia coli 89.0511 [enterobacteria]  Next Previous First | | | |
| tir [Escherichia coli 89.0511] | 77.4 | 7e-15 | EKV72371 |
| Escherichia coli 90.2281 [enterobacteria]  Next Previous First | | | |
| tir [Escherichia coli 90.2281] | 77.4 | 7e-15 | EKV90370 |
| Escherichia coli 93.0056 [enterobacteria]  Next Previous First | | | |
| tir [Escherichia coli 93.0056] | 77.4 | 7e-15 | EKW05913 |
| Escherichia coli 93.0055 [enterobacteria]  Next Previous First | | | |
| tir [Escherichia coli 93.0055] | 77.4 | 7e-15 | EKW06203 |
| Escherichia coli 94.0618 [enterobacteria]  Next Previous First | | | |
| tir [Escherichia coli 94.0618] | 77.4 | 7e-15 | EKW10279 |
| Escherichia coli 95.1288 [enterobacteria]  Next Previous First | | | |
| tir [Escherichia coli 95.1288] | 77.4 | 7e-15 | EKW26041 |
| Escherichia coli 96.0428 [enterobacteria]  Next Previous First | | | |
| tir [Escherichia coli 96.0428] | 77.4 | 7e-15 | EKW38046 |
| Escherichia coli 96.0427 [enterobacteria]  Next Previous First | | | |
| tir [Escherichia coli 96.0427] | 77.4 | 7e-15 | EKW40395 |
| Escherichia coli 96.0932 [enterobacteria]  Next Previous First | | | |
| tir [Escherichia coli 96.0932] | 77.4 | 7e-15 | EKW52881 |
| Escherichia coli 96.0107 [enterobacteria]  Next Previous First | | | |
| tir [Escherichia coli 96.0107] | 77.4 | 7e-15 | EKW59033 |
| Escherichia coli 97.0003 [enterobacteria]  Next Previous First | | | |
| tir [Escherichia coli 97.0003] | 77.4 | 7e-15 | EKW61057 |
| Escherichia coli 97.0007 [enterobacteria]  Next Previous First | | | |
| tir [Escherichia coli 97.0007] | 77.4 | 7e-15 | EKW73723 |
| Escherichia coli 99.0678 [enterobacteria]  Next Previous First | | | |
| intimin receptor protein [Escherichia coli 99.0678] | 77.4 | 7e-15 | EKW86645 |
| Escherichia coli 99.0713 [enterobacteria]  Next Previous First | | | |
| tir [Escherichia coli 99.0713] | 77.4 | 7e-15 | EKW87920 |
| Escherichia coli 96.0109 [enterobacteria]  Next Previous First | | | |
| tir [Escherichia coli 96.0109] | 77.4 | 7e-15 | EKY35822 |
| Escherichia coli 97.0010 [enterobacteria]  Next Previous First | | | |
| tir [Escherichia coli 97.0010] | 77.4 | 7e-15 | EKY36632 |
| Escherichia coli 09BKT078844 [enterobacteria]  Next Previous First | | | |
| tir [Escherichia coli 09BKT078844] | 77.4 | 7e-15 | ELV16600 |
| Escherichia coli 99.0839 [enterobacteria]  Next Previous First | | | |
| tir [Escherichia coli 99.0839] | 77.4 | 7e-15 | ELV32456 |
| Escherichia coli 99.0848 [enterobacteria]  Next Previous First | | | |
| tir [Escherichia coli 99.0848] | 77.4 | 7e-15 | ELV37090 |
| Escherichia coli PA11 [enterobacteria]  Next Previous First | | | |
| tir [Escherichia coli PA11] | 77.4 | 7e-15 | ELV64972 |
| Escherichia coli PA13 [enterobacteria]  Next Previous First | | | |
| tir [Escherichia coli PA13] | 77.4 | 7e-15 | ELV78234 |
| Escherichia coli PA19 [enterobacteria]  Next Previous First | | | |
| tir [Escherichia coli PA19] | 77.4 | 7e-15 | ELV78409 |
| Escherichia coli PA2 [enterobacteria]  Next Previous First | | | |
| tir [Escherichia coli PA2] | 77.4 | 7e-15 | ELV86874 |
| Escherichia coli PA47 [enterobacteria]  Next Previous First | | | |
| tir [Escherichia coli PA47] | 77.4 | 7e-15 | ELV94012 |
| Escherichia coli PA48 [enterobacteria]  Next Previous First | | | |
| tir [Escherichia coli PA48] | 77.4 | 7e-15 | ELV94631 |
| Escherichia coli PA8 [enterobacteria]  Next Previous First | | | |
| tir [Escherichia coli PA8] | 77.4 | 7e-15 | ELW00882 |
| Escherichia coli 7.1982 [enterobacteria]  Next Previous First | | | |
| tir [Escherichia coli 7.1982] | 77.4 | 7e-15 | ELW08835 |
| Escherichia coli 99.1762 [enterobacteria]  Next Previous First | | | |
| tir [Escherichia coli 99.1762] | 77.4 | 7e-15 | ELW15607 |
| Escherichia coli PA35 [enterobacteria]  Next Previous First | | | |
| tir [Escherichia coli PA35] | 77.4 | 7e-15 | ELW24614 |
| Escherichia coli 3.4880 [enterobacteria]  Next Previous First | | | |
| tir [Escherichia coli 3.4880] | 77.4 | 7e-15 | ELW29586 |
| Escherichia coli 95.0083 [enterobacteria]  Next Previous First | | | |
| tir [Escherichia coli 95.0083] | 77.4 | 7e-15 | ELW32480 |
| Escherichia coli 99.0670 [enterobacteria]  Next Previous First | | | |
| tir [Escherichia coli 99.0670] | 77.4 | 7e-15 | ELW39014 |
| Escherichia coli B28-1 [enterobacteria]  Next Previous First | | | |
| tir [Escherichia coli B28-1] | 77.4 | 7e-15 | ERB93502 |
| Escherichia coli B28-2 [enterobacteria]  Next Previous First | | | |
| tir [Escherichia coli B28-2] | 77.4 | 7e-15 | ERB94145 |
| Escherichia coli B29-1 [enterobacteria]  Next Previous First | | | |
| tir [Escherichia coli B29-1] | 77.4 | 7e-15 | ERC03037 |
| Escherichia coli B29-2 [enterobacteria]  Next Previous First | | | |
| tir [Escherichia coli B29-2] | 77.4 | 7e-15 | ERC10166 |
| Escherichia coli B36-1 [enterobacteria]  Next Previous First | | | |
| tir [Escherichia coli B36-1] | 77.4 | 7e-15 | ERC14434 |
| Escherichia coli B36-2 [enterobacteria]  Next Previous First | | | |
| tir [Escherichia coli B36-2] | 77.4 | 7e-15 | ERC18319 |
| Escherichia coli B7-1 [enterobacteria]  Next Previous First | | | |
| tir [Escherichia coli B7-1] | 77.4 | 7e-15 | ERC26355 |
| Escherichia coli B7-2 [enterobacteria]  Next Previous First | | | |
| tir [Escherichia coli B7-2] | 77.4 | 7e-15 | ERC31308 |
| Escherichia coli B93 [enterobacteria]  Next Previous First | | | |
| tir [Escherichia coli B93] | 77.4 | 7e-15 | ERC35728 |
| Escherichia coli B94 [enterobacteria]  Next Previous First | | | |
| tir [Escherichia coli B94] | 77.4 | 7e-15 | ERC41121 |
| Escherichia coli B95 [enterobacteria]  Next Previous First | | | |
| tir [Escherichia coli B95] | 77.4 | 7e-15 | ERC48922 |
| Escherichia coli Bd5610\_99 [enterobacteria]  Next Previous First | | | |
| tir [Escherichia coli Bd5610\_99] | 77.4 | 7e-15 | ERC62990 |
| Escherichia coli T1840\_97 [enterobacteria]  Next Previous First | | | |
| tir [Escherichia coli T1840\_97] | 77.4 | 7e-15 | ERC67332 |
| Escherichia coli T924\_01 [enterobacteria]  Next Previous First | | | |
| tir [Escherichia coli T924\_01] | 77.4 | 7e-15 | ERC81960 |
| Escherichia coli 2886-75 [enterobacteria]  Next Previous First | | | |
| tir [Escherichia coli 2886-75] | 77.4 | 7e-15 | ERC92228 |
| Escherichia coli B108 [enterobacteria]  Next Previous First | | | |
| tir [Escherichia coli B108] | 77.4 | 7e-15 | ERD11530 |
| Escherichia coli B109 [enterobacteria]  Next Previous First | | | |
| tir [Escherichia coli B109] | 77.4 | 7e-15 | ERD23759 |
| Escherichia coli B112 [enterobacteria]  Next Previous First | | | |
| tir [Escherichia coli B112] | 77.4 | 7e-15 | ERD25663 |
| Escherichia coli B113 [enterobacteria]  Next Previous First | | | |
| tir [Escherichia coli B113] | 77.4 | 7e-15 | ERD29345 |
| Escherichia coli B114 [enterobacteria]  Next Previous First | | | |
| tir [Escherichia coli B114] | 77.4 | 7e-15 | ERD38274 |
| Escherichia coli B15 [enterobacteria]  Next Previous First | | | |
| tir [Escherichia coli B15] | 77.4 | 7e-15 | ERD41803 |
| Escherichia coli B17 [enterobacteria]  Next Previous First | | | |
| tir [Escherichia coli B17] | 77.4 | 7e-15 | ERD46567 |
| Escherichia coli B5-2 [enterobacteria]  Next Previous First | | | |
| tir [Escherichia coli B5-2] | 77.4 | 7e-15 | ERD69676 |
| Escherichia coli 09BKT024447 [enterobacteria]  Next Previous First | | | |
| tir [Escherichia coli 09BKT024447] | 77.4 | 7e-15 | ERE11939 |
| Escherichia coli B89 [enterobacteria]  Next Previous First | | | |
| tir [Escherichia coli B89] | 77.4 | 7e-15 | ERE23499 |
| Escherichia coli B90 [enterobacteria]  Next Previous First | | | |
| tir [Escherichia coli B90] | 77.4 | 7e-15 | ERE25469 |
| Escherichia coli Tx1686 [enterobacteria]  Next Previous First | | | |
| tir [Escherichia coli Tx1686] | 77.4 | 7e-15 | ERE30452 |
| Escherichia coli Tx3800 [enterobacteria]  Next Previous First | | | |
| tir [Escherichia coli Tx3800] | 77.4 | 7e-15 | ERE38448 |
| Escherichia coli ATCC BAA-2192 [enterobacteria]  Next Previous First | | | |
| translocated intimin receptor Tir [Escherichia coli ATCC BAA-2192] | 77.4 | 7e-15 | ETJ81008 |
| Escherichia coli O157:H7 str. 2009EL2109 [enterobacteria]  Next Previous First | | | |
| translocated intimin receptor Tir [Escherichia coli O157:H7 str. 2009EL2109] | 77.4 | 7e-15 | EYV58195 |
| Escherichia coli O157:H7 str. 2009EL1705 [enterobacteria]  Next Previous First | | | |
| translocated intimin receptor Tir [Escherichia coli O157:H7 str. 2009EL1705] | 77.4 | 7e-15 | EYV67812 |
| Escherichia coli O157:H7 str. K5806 [enterobacteria]  Next Previous First | | | |
| translocated intimin receptor Tir [Escherichia coli O157:H7 str. K5806] | 77.4 | 7e-15 | EYV83475 |
| Escherichia coli O157:H7 str. 2011EL-2312 [enterobacteria]  Next Previous First | | | |
| translocated intimin receptor Tir [Escherichia coli O157:H7 str. 2011EL-2312] | 77.4 | 7e-15 | EYW04534 |
| Escherichia coli O157:H7 str. 2011EL-2289 [enterobacteria]  Next Previous First | | | |
| translocated intimin receptor Tir [Escherichia coli O157:H7 str. 2011EL-2289] | 77.4 | 7e-15 | EYW05684 |
| Escherichia coli O157:H7 str. 2011EL-2288 [enterobacteria]  Next Previous First | | | |
| translocated intimin receptor Tir [Escherichia coli O157:H7 str. 2011EL-2288] | 77.4 | 7e-15 | EYW09513 |
| Escherichia coli O157:H7 str. 2011EL-2114 [enterobacteria]  Next Previous First | | | |
| translocated intimin receptor Tir [Escherichia coli O157:H7 str. 2011EL-2114] | 77.4 | 7e-15 | EYW14157 |
| Escherichia coli O157:H7 str. 2011EL-2287 [enterobacteria]  Next Previous First | | | |
| translocated intimin receptor Tir [Escherichia coli O157:H7 str. 2011EL-2287] | 77.4 | 7e-15 | EYW16893 |
| Escherichia coli O157:H7 str. 2011EL-2286 [enterobacteria]  Next Previous First | | | |
| translocated intimin receptor Tir [Escherichia coli O157:H7 str. 2011EL-2286] | 77.4 | 7e-15 | EYW25028 |
| Escherichia coli O157:H7 str. 2011EL-2113 [enterobacteria]  Next Previous First | | | |
| translocated intimin receptor Tir [Escherichia coli O157:H7 str. 2011EL-2113] | 77.4 | 7e-15 | EYW29607 |
| Escherichia coli O157:H7 str. 2011EL-2112 [enterobacteria]  Next Previous First | | | |
| translocated intimin receptor Tir [Escherichia coli O157:H7 str. 2011EL-2112] | 77.4 | 7e-15 | EYW32758 |
| Escherichia coli O157:H7 str. 2011EL-2111 [enterobacteria]  Next Previous First | | | |
| translocated intimin receptor Tir [Escherichia coli O157:H7 str. 2011EL-2111] | 77.4 | 7e-15 | EYW41392 |
| Escherichia coli O157:H7 str. 2011EL-2108 [enterobacteria]  Next Previous First | | | |
| translocated intimin receptor Tir [Escherichia coli O157:H7 str. 2011EL-2108] | 77.4 | 7e-15 | EYW48366 |
| Escherichia coli O157:H7 str. 2011EL-2109 [enterobacteria]  Next Previous First | | | |
| translocated intimin receptor Tir [Escherichia coli O157:H7 str. 2011EL-2109] | 77.4 | 7e-15 | EYW52048 |
| Escherichia coli O157:H7 str. 2011EL-2107 [enterobacteria]  Next Previous First | | | |
| translocated intimin receptor Tir [Escherichia coli O157:H7 str. 2011EL-2107] | 77.4 | 7e-15 | EYW53605 |
| Escherichia coli O157:H7 str. 2011EL-2106 [enterobacteria]  Next Previous First | | | |
| translocated intimin receptor Tir [Escherichia coli O157:H7 str. 2011EL-2106] | 77.4 | 7e-15 | EYW61342 |
| Escherichia coli O157:H7 str. 2011EL-2105 [enterobacteria]  Next Previous First | | | |
| translocated intimin receptor Tir [Escherichia coli O157:H7 str. 2011EL-2105] | 77.4 | 7e-15 | EYW63256 |
| Escherichia coli O157:H7 str. 2011EL-2104 [enterobacteria]  Next Previous First | | | |
| translocated intimin receptor Tir [Escherichia coli O157:H7 str. 2011EL-2104] | 77.4 | 7e-15 | EYW71647 |
| Escherichia coli O157:H7 str. 2011EL-2103 [enterobacteria]  Next Previous First | | | |
| translocated intimin receptor Tir [Escherichia coli O157:H7 str. 2011EL-2103] | 77.4 | 7e-15 | EYW78535 |
| Escherichia coli O157:H7 str. 2011EL-2101 [enterobacteria]  Next Previous First | | | |
| translocated intimin receptor Tir [Escherichia coli O157:H7 str. 2011EL-2101] | 77.4 | 7e-15 | EYW79639 |
| Escherichia coli O157:H7 str. 2011EL-2099 [enterobacteria]  Next Previous First | | | |
| translocated intimin receptor Tir [Escherichia coli O157:H7 str. 2011EL-2099] | 77.4 | 7e-15 | EYW81999 |
| Escherichia coli O157:H7 str. 08-4169 [enterobacteria]  Next Previous First | | | |
| translocated intimin receptor Tir [Escherichia coli O157:H7 str. 08-4169] | 77.4 | 7e-15 | EYX00539 |
| Escherichia coli O157:H7 str. 08-3037 [enterobacteria]  Next Previous First | | | |
| translocated intimin receptor Tir [Escherichia coli O157:H7 str. 08-3037] | 77.4 | 7e-15 | EYX14612 |
| Escherichia coli O157:H7 str. 08-3527 [enterobacteria]  Next Previous First | | | |
| translocated intimin receptor Tir [Escherichia coli O157:H7 str. 08-3527] | 77.4 | 7e-15 | EYX15355 |
| Escherichia coli O157:H7 str. 2011EL-2098 [enterobacteria]  Next Previous First | | | |
| translocated intimin receptor Tir [Escherichia coli O157:H7 str. 2011EL-2098] | 77.4 | 7e-15 | EYX23843 |
| Escherichia coli O157:H7 str. 2011EL-2097 [enterobacteria]  Next Previous First | | | |
| translocated intimin receptor Tir [Escherichia coli O157:H7 str. 2011EL-2097] | 77.4 | 7e-15 | EYX24314 |
| Escherichia coli O157:H7 str. 2011EL-2096 [enterobacteria]  Next Previous First | | | |
| translocated intimin receptor Tir [Escherichia coli O157:H7 str. 2011EL-2096] | 77.4 | 7e-15 | EYX34486 |
| Escherichia coli O157:H7 str. 2011EL-2094 [enterobacteria]  Next Previous First | | | |
| translocated intimin receptor Tir [Escherichia coli O157:H7 str. 2011EL-2094] | 77.4 | 7e-15 | EYX38559 |
| Escherichia coli O157:H7 str. 2011EL-2093 [enterobacteria]  Next Previous First | | | |
| translocated intimin receptor Tir [Escherichia coli O157:H7 str. 2011EL-2093] | 77.4 | 7e-15 | EYX40279 |
| Escherichia coli O157:H7 str. 2011EL-2092 [enterobacteria]  Next Previous First | | | |
| translocated intimin receptor Tir [Escherichia coli O157:H7 str. 2011EL-2092] | 77.4 | 7e-15 | EYX50075 |
| Escherichia coli O157:H7 str. 2011EL-2091 [enterobacteria]  Next Previous First | | | |
| translocated intimin receptor Tir [Escherichia coli O157:H7 str. 2011EL-2091] | 77.4 | 7e-15 | EYX52621 |
| Escherichia coli O157:H7 str. 2011EL-2090 [enterobacteria]  Next Previous First | | | |
| translocated intimin receptor Tir [Escherichia coli O157:H7 str. 2011EL-2090] | 77.4 | 7e-15 | EYX59077 |
| Escherichia coli O157:H7 str. 2011EL-1107 [enterobacteria]  Next Previous First | | | |
| translocated intimin receptor Tir [Escherichia coli O157:H7 str. 2011EL-1107] | 77.4 | 7e-15 | EYX62836 |
| Escherichia coli O157:H7 str. 2010C-4979C1 [enterobacteria]  Next Previous First | | | |
| translocated intimin receptor Tir [Escherichia coli O157:H7 str. 2010C-4979C1] | 77.4 | 7e-15 | EYY50539 |
| Escherichia coli O157:H7 str. 07-3091 [enterobacteria]  Next Previous First | | | |
| translocated intimin receptor Tir [Escherichia coli O157:H7 str. 07-3091] | 77.4 | 7e-15 | EYZ28257 |
| Escherichia coli O157:H7 str. 06-4039 [enterobacteria]  Next Previous First | | | |
| translocated intimin receptor Tir [Escherichia coli O157:H7 str. 06-4039] | 77.4 | 7e-15 | EYZ28988 |
| Escherichia coli O157:H7 str. 07-3391 [enterobacteria]  Next Previous First | | | |
| translocated intimin receptor Tir [Escherichia coli O157:H7 str. 07-3391] | 77.4 | 7e-15 | EYZ33426 |
| Escherichia coli O157:H7 str. 06-3745 [enterobacteria]  Next Previous First | | | |
| translocated intimin receptor Tir [Escherichia coli O157:H7 str. 06-3745] | 77.4 | 7e-15 | EYZ50143 |
| Escherichia coli O157:H7 str. F6142 [enterobacteria]  Next Previous First | | | |
| translocated intimin receptor Tir [Escherichia coli O157:H7 str. F6142] | 77.4 | 7e-15 | EZA83296 |
| Escherichia coli O157:H7 str. F6750 [enterobacteria]  Next Previous First | | | |
| translocated intimin receptor Tir [Escherichia coli O157:H7 str. F6750] | 77.4 | 7e-15 | EZA98203 |
| Escherichia coli O157:H7 str. F6749 [enterobacteria]  Next Previous First | | | |
| translocated intimin receptor Tir [Escherichia coli O157:H7 str. F6749] | 77.4 | 7e-15 | EZB04978 |
| Escherichia coli O157:H7 str. F6751 [enterobacteria]  Next Previous First | | | |
| translocated intimin receptor Tir [Escherichia coli O157:H7 str. F6751] | 77.4 | 7e-15 | EZB08102 |
| Escherichia coli O157:H7 str. F7384 [enterobacteria]  Next Previous First | | | |
| translocated intimin receptor Tir [Escherichia coli O157:H7 str. F7384] | 77.4 | 7e-15 | EZB15670 |
| Escherichia coli O157:H7 str. F7410 [enterobacteria]  Next Previous First | | | |
| translocated intimin receptor Tir [Escherichia coli O157:H7 str. F7410] | 77.4 | 7e-15 | EZB24242 |
| Escherichia coli O157:H7 str. G5303 [enterobacteria]  Next Previous First | | | |
| translocated intimin receptor Tir [Escherichia coli O157:H7 str. G5303] | 77.4 | 7e-15 | EZB32641 |
| Escherichia coli O157:H7 str. H2495 [enterobacteria]  Next Previous First | | | |
| translocated intimin receptor Tir [Escherichia coli O157:H7 str. H2495] | 77.4 | 7e-15 | EZB43747 |
| Escherichia coli O157:H7 str. K1420 [enterobacteria]  Next Previous First | | | |
| translocated intimin receptor Tir [Escherichia coli O157:H7 str. K1420] | 77.4 | 7e-15 | EZB44245 |
| Escherichia coli O157:H7 str. H2498 [enterobacteria]  Next Previous First | | | |
| translocated intimin receptor Tir [Escherichia coli O157:H7 str. H2498] | 77.4 | 7e-15 | EZB47735 |
| Escherichia coli O157:H7 str. K1792 [enterobacteria]  Next Previous First | | | |
| translocated intimin receptor Tir [Escherichia coli O157:H7 str. K1792] | 77.4 | 7e-15 | EZB50823 |
| Escherichia coli O157:H7 str. K1845 [enterobacteria]  Next Previous First | | | |
| translocated intimin receptor Tir [Escherichia coli O157:H7 str. K1845] | 77.4 | 7e-15 | EZB69816 |
| Escherichia coli O157:H7 str. K1796 [enterobacteria]  Next Previous First | | | |
| translocated intimin receptor Tir [Escherichia coli O157:H7 str. K1796] | 77.4 | 7e-15 | EZB72061 |
| Escherichia coli O157:H7 str. K1921 [enterobacteria]  Next Previous First | | | |
| translocated intimin receptor Tir [Escherichia coli O157:H7 str. K1921] | 77.4 | 7e-15 | EZB81670 |
| Escherichia coli O157:H7 str. K2188 [enterobacteria]  Next Previous First | | | |
| translocated intimin receptor Tir [Escherichia coli O157:H7 str. K2188] | 77.4 | 7e-15 | EZB84004 |
| Escherichia coli O157:H7 str. K2191 [enterobacteria]  Next Previous First | | | |
| translocated intimin receptor Tir [Escherichia coli O157:H7 str. K2191] | 77.4 | 7e-15 | EZB95741 |
| Escherichia coli O157:H7 str. K2192 [enterobacteria]  Next Previous First | | | |
| translocated intimin receptor Tir [Escherichia coli O157:H7 str. K2192] | 77.4 | 7e-15 | EZC01830 |
| Escherichia coli O157:H7 str. K2581 [enterobacteria]  Next Previous First | | | |
| translocated intimin receptor Tir [Escherichia coli O157:H7 str. K2581] | 77.4 | 7e-15 | EZC10302 |
| Escherichia coli O157:H7 str. K2622 [enterobacteria]  Next Previous First | | | |
| translocated intimin receptor Tir [Escherichia coli O157:H7 str. K2622] | 77.4 | 7e-15 | EZC17846 |
| Escherichia coli O157:H7 str. K2845 [enterobacteria]  Next Previous First | | | |
| translocated intimin receptor Tir [Escherichia coli O157:H7 str. K2845] | 77.4 | 7e-15 | EZC18601 |
| Escherichia coli O157:H7 str. K2854 [enterobacteria]  Next Previous First | | | |
| translocated intimin receptor Tir [Escherichia coli O157:H7 str. K2854] | 77.4 | 7e-15 | EZC21335 |
| Escherichia coli O157:H7 str. K4406 [enterobacteria]  Next Previous First | | | |
| translocated intimin receptor Tir [Escherichia coli O157:H7 str. K4406] | 77.4 | 7e-15 | EZC31962 |
| Escherichia coli O157:H7 str. K4396 [enterobacteria]  Next Previous First | | | |
| translocated intimin receptor Tir [Escherichia coli O157:H7 str. K4396] | 77.4 | 7e-15 | EZC33548 |
| Escherichia coli O157:H7 str. K4405 [enterobacteria]  Next Previous First | | | |
| translocated intimin receptor Tir [Escherichia coli O157:H7 str. K4405] | 77.4 | 7e-15 | EZC36100 |
| Escherichia coli O157:H7 str. K4527 [enterobacteria]  Next Previous First | | | |
| translocated intimin receptor Tir [Escherichia coli O157:H7 str. K4527] | 77.4 | 7e-15 | EZC46313 |
| Escherichia coli O157:H7 str. K5418 [enterobacteria]  Next Previous First | | | |
| translocated intimin receptor Tir [Escherichia coli O157:H7 str. K5418] | 77.4 | 7e-15 | EZC56440 |
| Escherichia coli O157:H7 str. K5448 [enterobacteria]  Next Previous First | | | |
| translocated intimin receptor Tir [Escherichia coli O157:H7 str. K5448] | 77.4 | 7e-15 | EZC66996 |
| Escherichia coli O157:H7 str. K5453 [enterobacteria]  Next Previous First | | | |
| translocated intimin receptor Tir [Escherichia coli O157:H7 str. K5453] | 77.4 | 7e-15 | EZC71479 |
| Escherichia coli O157:H7 str. K5449 [enterobacteria]  Next Previous First | | | |
| translocated intimin receptor Tir [Escherichia coli O157:H7 str. K5449] | 77.4 | 7e-15 | EZC73722 |
| Escherichia coli O157:H7 str. K5460 [enterobacteria]  Next Previous First | | | |
| translocated intimin receptor Tir [Escherichia coli O157:H7 str. K5460] | 77.4 | 7e-15 | EZC80937 |
| Escherichia coli O157:H7 str. K5467 [enterobacteria]  Next Previous First | | | |
| translocated intimin receptor Tir [Escherichia coli O157:H7 str. K5467] | 77.4 | 7e-15 | EZC87704 |
| Escherichia coli O157:H7 str. K5602 [enterobacteria]  Next Previous First | | | |
| translocated intimin receptor Tir [Escherichia coli O157:H7 str. K5602] | 77.4 | 7e-15 | EZC91873 |
| Escherichia coli O157:H7 str. K5609 [enterobacteria]  Next Previous First | | | |
| translocated intimin receptor Tir [Escherichia coli O157:H7 str. K5609] | 77.4 | 7e-15 | EZC94046 |
| Escherichia coli O157:H7 str. K5607 [enterobacteria]  Next Previous First | | | |
| translocated intimin receptor Tir [Escherichia coli O157:H7 str. K5607] | 77.4 | 7e-15 | EZC95803 |
| Escherichia coli O157:H7 str. K5852 [enterobacteria]  Next Previous First | | | |
| translocated intimin receptor Tir [Escherichia coli O157:H7 str. K5852] | 77.4 | 7e-15 | EZD00921 |
| Escherichia coli O157:H7 str. K6590 [enterobacteria]  Next Previous First | | | |
| translocated intimin receptor Tir [Escherichia coli O157:H7 str. K6590] | 77.4 | 7e-15 | EZD10962 |
| Escherichia coli O157:H7 str. K6676 [enterobacteria]  Next Previous First | | | |
| translocated intimin receptor Tir [Escherichia coli O157:H7 str. K6676] | 77.4 | 7e-15 | EZD12048 |
| Escherichia coli O157:H7 str. K6687 [enterobacteria]  Next Previous First | | | |
| translocated intimin receptor Tir [Escherichia coli O157:H7 str. K6687] | 77.4 | 7e-15 | EZD21486 |
| Escherichia coli O157:H7 str. K7140 [enterobacteria]  Next Previous First | | | |
| translocated intimin receptor Tir [Escherichia coli O157:H7 str. K7140] | 77.4 | 7e-15 | EZD73719 |
| Escherichia coli O157:H7 str. 08-4529 [enterobacteria]  Next Previous First | | | |
| translocated intimin receptor Tir [Escherichia coli O157:H7 str. 08-4529] | 77.4 | 7e-15 | EZD80823 |
| Escherichia coli O157:H7 str. 2009EL1913 [enterobacteria]  Next Previous First | | | |
| translocated intimin receptor Tir [Escherichia coli O157:H7 str. 2009EL1913] | 77.4 | 7e-15 | EZE80300 |
| Escherichia coli O157:H7 str. 2011EL-2313 [enterobacteria]  Next Previous First | | | |
| translocated intimin receptor Tir [Escherichia coli O157:H7 str. 2011EL-2313] | 77.4 | 7e-15 | EZF06439 |
| Escherichia coli O157:H7 str. 2011EL-2290 [enterobacteria]  Next Previous First | | | |
| translocated intimin receptor Tir [Escherichia coli O157:H7 str. 2011EL-2290] | 77.4 | 7e-15 | EZF06643 |
| Escherichia coli O157: str. 2010EL-2045 [enterobacteria]  Next Previous First | | | |
| translocated intimin receptor Tir [Escherichia coli O157: str. 2010EL-2045] | 77.4 | 7e-15 | EZQ40147 |
| Escherichia coli O157: str. 2010EL-2044 [enterobacteria]  Next Previous First | | | |
| translocated intimin receptor Tir [Escherichia coli O157: str. 2010EL-2044] | 77.4 | 7e-15 | EZQ53276 |
| Escherichia coli SHECO003 [enterobacteria]  Next Previous First | | | |
| hypothetical protein L317\_13220 [Escherichia coli SHECO003] | 77.4 | 7e-15 | OSM85740 |
| Escherichia coli O157:H7 str. EC1825 [enterobacteria]  Next Previous First | | | |
| translocated intimin receptor Tir [Escherichia coli O157:H7 str. EC1825] | 77.4 | 7e-15 | PJR44496 |
| Escherichia coli O157 [enterobacteria]  Next Previous First | | | |
| type III secretion system LEE translocated intimin receptor Tir [Escherichia coli O157] | 77.4 | 7e-15 | PNL71000 |
| type III secretion system LEE translocated intimin receptor Tir [Escherichia coli O157] | 77.4 | 7e-15 | AVJ53171 |
| Escherichia coli O55:H7 str. USDA 5905 [enterobacteria]  Next Previous First | | | |
| Translocated intimin receptor Tir [Escherichia coli O55:H7 str. USDA 5905] | 77.4 | 7e-15 | EFX28470 |
| translocated intimin receptor Tir [Escherichia coli O55:H7 str. USDA 5905] | 77.4 | 7e-15 | PIS72829 |
| Escherichia coli DEC5E [enterobacteria]  Next Previous First | | | |
| putative translocated intimin receptor protein [Escherichia coli DEC5E] | 77.4 | 7e-15 | EHV44371 |
| Escherichia coli 5905 [enterobacteria]  Next Previous First | | | |
| translocated intimin receptor [Escherichia coli 5905] | 77.4 | 7e-15 | EKH89324 |
| Escherichia coli 09BKT076207 [enterobacteria]  Next Previous First | | | |
| tir [Escherichia coli 09BKT076207] | 77.4 | 7e-15 | ERB69919 |
| Escherichia coli O55:H7 str. CB9615 [enterobacteria]  Next Previous First | | | |
| RecName: Full=Translocated intimin receptor Tir; AltName: Full=Secreted effector protein Tir [Escherichia coli O55:H7 str. CB9615] | 77.4 | 7e-15 | D3QW22 |
| Translocated intimin receptor Tir [Escherichia coli O55:H7 str. CB9615] | 77.4 | 7e-15 | ADD58883 |
| Escherichia coli O55:H7 str. 3256-97 [enterobacteria]  Next Previous First | | | |
| Translocated intimin receptor Tir [Escherichia coli O55:H7 str. 3256-97] | 77.4 | 7e-15 | EFX23342 |
| Escherichia coli DEC4A [enterobacteria]  Next Previous First | | | |
| putative translocated intimin receptor protein [Escherichia coli DEC4A] | 77.4 | 7e-15 | EHU87073 |
| Escherichia coli DEC5A [enterobacteria]  Next Previous First | | | |
| putative translocated intimin receptor protein [Escherichia coli DEC5A] | 77.4 | 7e-15 | EHV21047 |
| Escherichia coli DEC5B [enterobacteria]  Next Previous First | | | |
| putative translocated intimin receptor protein [Escherichia coli DEC5B] | 77.4 | 7e-15 | EHV25219 |
| Escherichia coli PA22 [enterobacteria]  Next Previous First | | | |
| translocated intimin receptor Tir [Escherichia coli PA22] | 77.4 | 7e-15 | EIN84433 |
| Escherichia coli TW10246 [enterobacteria]  Next Previous First | | | |
| translocated intimin receptor Tir [Escherichia coli TW10246] | 77.4 | 7e-15 | EIO54787 |
| Escherichia coli 8.0416 [enterobacteria]  Next Previous First | | | |
| translocated intimin receptor Tir [Escherichia coli 8.0416] | 77.4 | 7e-15 | EKK72960 |
| Escherichia coli O55:H7 str. 06-3555 [enterobacteria]  Next Previous First | | | |
| translocated intimin receptor Tir [Escherichia coli O55:H7 str. 06-3555] | 77.4 | 7e-15 | EYZ54527 |
| Escherichia coli 88.1467 [enterobacteria]  Next Previous First | | | |
| tir [Escherichia coli 88.1467] | 77.4 | 7e-15 | EKV75105 |
| Escherichia coli TW11039 [enterobacteria]  Next Previous First | | | |
| putative translocated intimin receptor protein [Escherichia coli TW11039] | 77.4 | 7e-15 | EIO60454 |
| Escherichia coli FRIK1997 [enterobacteria]  Next Previous First | | | |
| putative translocated intimin receptor protein [Escherichia coli FRIK1997] | 77.4 | 7e-15 | EKH33350 |
| Escherichia coli EC1735 [enterobacteria]  Next Previous First | | | |
| putative translocated intimin receptor protein [Escherichia coli EC1735] | 77.4 | 7e-15 | EKI47661 |
| Escherichia coli EC1737 [enterobacteria]  Next Previous First | | | |
| putative translocated intimin receptor protein [Escherichia coli EC1737] | 77.4 | 7e-15 | EKI61410 |
| Escherichia coli 88.0221 [enterobacteria]  Next Previous First | | | |
| tir [Escherichia coli 88.0221] | 77.4 | 7e-15 | EKK67725 |
| Escherichia coli DEC4B [enterobacteria]  Next Previous First | | | |
| putative translocated intimin receptor protein [Escherichia coli DEC4B] | 77.4 | 7e-15 | EHU91785 |
| Escherichia coli ATCC 35150 [enterobacteria]  Next Previous First | | | |
| translocated intimin receptor Tir [Escherichia coli ATCC 35150] | 77.4 | 7e-15 | ETJ70976 |
| Escherichia coli O157:H7 str. K1927 [enterobacteria]  Next Previous First | | | |
| translocated intimin receptor Tir, partial [Escherichia coli O157:H7 str. K1927] | 77.4 | 7e-15 | EZB82918 |
| Escherichia coli O157:H7 str. 2009EL1449 [enterobacteria]  Next Previous First | | | |
| translocated intimin receptor Tir, partial [Escherichia coli O157:H7 str. 2009EL1449] | 77.4 | 7e-15 | EZE79134 |
| Escherichia coli 97.1742 [enterobacteria]  Next Previous First | | | |
| tir [Escherichia coli 97.1742] | 77.4 | 7e-15 | EKW70817 |
| Escherichia coli DEC4D [enterobacteria]  Next Previous First | | | |
| putative translocated intimin receptor protein [Escherichia coli DEC4D] | 77.4 | 7e-15 | EHV01565 |
| Escherichia coli DEC3D [enterobacteria]  Next Previous First | | | |
| putative translocated intimin receptor protein [Escherichia coli DEC3D] | 77.4 | 7e-15 | EHU70585 |
| Escherichia coli DEC3E [enterobacteria]  Next Previous First | | | |
| putative translocated intimin receptor protein [Escherichia coli DEC3E] | 77.4 | 7e-15 | EHU71809 |
| Escherichia coli DEC4C [enterobacteria]  Next Previous First | | | |
| putative translocated intimin receptor protein [Escherichia coli DEC4C] | 77.4 | 7e-15 | EHV01826 |
| Salmonella enterica subsp. enterica [enterobacteria]  Next Previous First | | | |
| type III secretion system LEE translocated intimin receptor Tir [Salmonella enterica subsp. enterica] | 77.4 | 7e-15 | EAB7041911 |
| Escherichia coli O157:H7 str. K2324 [enterobacteria]  Next Previous First | | | |
| translocated intimin receptor Tir, partial [Escherichia coli O157:H7 str. K2324] | 77.4 | 7e-15 | EZC00076 |

## Taxonomy Report

Organism Report
Lineage Report

Taxonomy Report

| Taxonomy | Number of hits | Number of Organisms | Description |
| --- | --- | --- | --- |
| root | 1288 | 367 |  |
| .  Enterobacteriaceae | 1286 | 366 |  |
| ..  Escherichia | 1281 | 363 |  |
| ...  Escherichia coli | 871 | 363 | Escherichia coli hits |
| ....  Escherichia coli O157:H7 | 37 | 113 | Escherichia coli O157:H7 hits |
| .....  Escherichia coli O157:H7 str. EDL933 | 6 | 1 | Escherichia coli O157:H7 str. EDL933 hits |
| .....  Escherichia coli O157:H7 str. EC869 | 1 | 1 | Escherichia coli O157:H7 str. EC869 hits |
| .....  Escherichia coli O157:H7 str. 2009C-4258 | 1 | 1 | Escherichia coli O157:H7 str. 2009C-4258 hits |
| .....  Escherichia coli O157:H7 str. F7350 | 1 | 1 | Escherichia coli O157:H7 str. F7350 hits |
| .....  Escherichia coli O157:H7 str. F7377 | 1 | 1 | Escherichia coli O157:H7 str. F7377 hits |
| .....  Escherichia coli O157:H7 str. Sakai | 2 | 1 | Escherichia coli O157:H7 str. Sakai hits |
| .....  Escherichia coli O157:H7 str. EC4115 | 2 | 1 | Escherichia coli O157:H7 str. EC4115 hits |
| .....  Escherichia coli O157:H7 str. TW14359 | 2 | 1 | Escherichia coli O157:H7 str. TW14359 hits |
| .....  Escherichia coli O157:H7 str. SS17 | 1 | 1 | Escherichia coli O157:H7 str. SS17 hits |
| .....  Escherichia coli O157:H7 str. SS52 | 1 | 1 | Escherichia coli O157:H7 str. SS52 hits |
| .....  Escherichia coli O157:H7 str. EC4196 | 1 | 1 | Escherichia coli O157:H7 str. EC4196 hits |
| .....  Escherichia coli O157:H7 str. EC4113 | 1 | 1 | Escherichia coli O157:H7 str. EC4113 hits |
| .....  Escherichia coli O157:H7 str. EC4076 | 1 | 1 | Escherichia coli O157:H7 str. EC4076 hits |
| .....  Escherichia coli O157:H7 str. EC4401 | 1 | 1 | Escherichia coli O157:H7 str. EC4401 hits |
| .....  Escherichia coli O157:H7 str. EC4486 | 1 | 1 | Escherichia coli O157:H7 str. EC4486 hits |
| .....  Escherichia coli O157:H7 str. EC4501 | 1 | 1 | Escherichia coli O157:H7 str. EC4501 hits |
| .....  Escherichia coli O157:H7 str. EC508 | 1 | 1 | Escherichia coli O157:H7 str. EC508 hits |
| .....  Escherichia coli O157:H7 str. EC4206 | 1 | 1 | Escherichia coli O157:H7 str. EC4206 hits |
| .....  Escherichia coli O157:H7 str. EC4045 | 1 | 1 | Escherichia coli O157:H7 str. EC4045 hits |
| .....  Escherichia coli O157:H7 str. EC4042 | 1 | 1 | Escherichia coli O157:H7 str. EC4042 hits |
| .....  Escherichia coli O157:H7 str. TW14588 | 1 | 1 | Escherichia coli O157:H7 str. TW14588 hits |
| .....  Escherichia coli O157:H7 str. EC1212 | 1 | 1 | Escherichia coli O157:H7 str. EC1212 hits |
| .....  Escherichia coli O157:H7 str. G5101 | 1 | 1 | Escherichia coli O157:H7 str. G5101 hits |
| .....  Escherichia coli O157:H7 str. LSU-61 | 1 | 1 | Escherichia coli O157:H7 str. LSU-61 hits |
| .....  Escherichia coli O157:H7 str. 1044 | 1 | 1 | Escherichia coli O157:H7 str. 1044 hits |
| .....  Escherichia coli O157:H7 str. 1125 | 1 | 1 | Escherichia coli O157:H7 str. 1125 hits |
| .....  Escherichia coli O157:H7 str. TW14313 | 2 | 1 | Escherichia coli O157:H7 str. TW14313 hits |
| .....  Escherichia coli O157:H7 str. 2009EL2109 | 1 | 1 | Escherichia coli O157:H7 str. 2009EL2109 hits |
| .....  Escherichia coli O157:H7 str. 2009EL1705 | 1 | 1 | Escherichia coli O157:H7 str. 2009EL1705 hits |
| .....  Escherichia coli O157:H7 str. K5806 | 1 | 1 | Escherichia coli O157:H7 str. K5806 hits |
| .....  Escherichia coli O157:H7 str. 2011EL-2312 | 1 | 1 | Escherichia coli O157:H7 str. 2011EL-2312 hits |
| .....  Escherichia coli O157:H7 str. 2011EL-2289 | 1 | 1 | Escherichia coli O157:H7 str. 2011EL-2289 hits |
| .....  Escherichia coli O157:H7 str. 2011EL-2288 | 1 | 1 | Escherichia coli O157:H7 str. 2011EL-2288 hits |
| .....  Escherichia coli O157:H7 str. 2011EL-2114 | 1 | 1 | Escherichia coli O157:H7 str. 2011EL-2114 hits |
| .....  Escherichia coli O157:H7 str. 2011EL-2287 | 1 | 1 | Escherichia coli O157:H7 str. 2011EL-2287 hits |
| .....  Escherichia coli O157:H7 str. 2011EL-2286 | 1 | 1 | Escherichia coli O157:H7 str. 2011EL-2286 hits |
| .....  Escherichia coli O157:H7 str. 2011EL-2113 | 1 | 1 | Escherichia coli O157:H7 str. 2011EL-2113 hits |
| .....  Escherichia coli O157:H7 str. 2011EL-2112 | 1 | 1 | Escherichia coli O157:H7 str. 2011EL-2112 hits |
| .....  Escherichia coli O157:H7 str. 2011EL-2111 | 1 | 1 | Escherichia coli O157:H7 str. 2011EL-2111 hits |
| .....  Escherichia coli O157:H7 str. 2011EL-2108 | 1 | 1 | Escherichia coli O157:H7 str. 2011EL-2108 hits |
| .....  Escherichia coli O157:H7 str. 2011EL-2109 | 1 | 1 | Escherichia coli O157:H7 str. 2011EL-2109 hits |
| .....  Escherichia coli O157:H7 str. 2011EL-2107 | 1 | 1 | Escherichia coli O157:H7 str. 2011EL-2107 hits |
| .....  Escherichia coli O157:H7 str. 2011EL-2106 | 1 | 1 | Escherichia coli O157:H7 str. 2011EL-2106 hits |
| .....  Escherichia coli O157:H7 str. 2011EL-2105 | 1 | 1 | Escherichia coli O157:H7 str. 2011EL-2105 hits |
| .....  Escherichia coli O157:H7 str. 2011EL-2104 | 1 | 1 | Escherichia coli O157:H7 str. 2011EL-2104 hits |
| .....  Escherichia coli O157:H7 str. 2011EL-2103 | 1 | 1 | Escherichia coli O157:H7 str. 2011EL-2103 hits |
| .....  Escherichia coli O157:H7 str. 2011EL-2101 | 1 | 1 | Escherichia coli O157:H7 str. 2011EL-2101 hits |
| .....  Escherichia coli O157:H7 str. 2011EL-2099 | 1 | 1 | Escherichia coli O157:H7 str. 2011EL-2099 hits |
| .....  Escherichia coli O157:H7 str. 08-4169 | 1 | 1 | Escherichia coli O157:H7 str. 08-4169 hits |
| .....  Escherichia coli O157:H7 str. 08-3037 | 1 | 1 | Escherichia coli O157:H7 str. 08-3037 hits |
| .....  Escherichia coli O157:H7 str. 08-3527 | 1 | 1 | Escherichia coli O157:H7 str. 08-3527 hits |
| .....  Escherichia coli O157:H7 str. 2011EL-2098 | 1 | 1 | Escherichia coli O157:H7 str. 2011EL-2098 hits |
| .....  Escherichia coli O157:H7 str. 2011EL-2097 | 1 | 1 | Escherichia coli O157:H7 str. 2011EL-2097 hits |
| .....  Escherichia coli O157:H7 str. 2011EL-2096 | 1 | 1 | Escherichia coli O157:H7 str. 2011EL-2096 hits |
| .....  Escherichia coli O157:H7 str. 2011EL-2094 | 1 | 1 | Escherichia coli O157:H7 str. 2011EL-2094 hits |
| .....  Escherichia coli O157:H7 str. 2011EL-2093 | 1 | 1 | Escherichia coli O157:H7 str. 2011EL-2093 hits |
| .....  Escherichia coli O157:H7 str. 2011EL-2092 | 1 | 1 | Escherichia coli O157:H7 str. 2011EL-2092 hits |
| .....  Escherichia coli O157:H7 str. 2011EL-2091 | 1 | 1 | Escherichia coli O157:H7 str. 2011EL-2091 hits |
| .....  Escherichia coli O157:H7 str. 2011EL-2090 | 1 | 1 | Escherichia coli O157:H7 str. 2011EL-2090 hits |
| .....  Escherichia coli O157:H7 str. 2011EL-1107 | 1 | 1 | Escherichia coli O157:H7 str. 2011EL-1107 hits |
| .....  Escherichia coli O157:H7 str. 2010C-4979C1 | 1 | 1 | Escherichia coli O157:H7 str. 2010C-4979C1 hits |
| .....  Escherichia coli O157:H7 str. 07-3091 | 1 | 1 | Escherichia coli O157:H7 str. 07-3091 hits |
| .....  Escherichia coli O157:H7 str. 06-4039 | 1 | 1 | Escherichia coli O157:H7 str. 06-4039 hits |
| .....  Escherichia coli O157:H7 str. 07-3391 | 1 | 1 | Escherichia coli O157:H7 str. 07-3391 hits |
| .....  Escherichia coli O157:H7 str. 06-3745 | 1 | 1 | Escherichia coli O157:H7 str. 06-3745 hits |
| .....  Escherichia coli O157:H7 str. F6142 | 1 | 1 | Escherichia coli O157:H7 str. F6142 hits |
| .....  Escherichia coli O157:H7 str. F6750 | 1 | 1 | Escherichia coli O157:H7 str. F6750 hits |
| .....  Escherichia coli O157:H7 str. F6749 | 1 | 1 | Escherichia coli O157:H7 str. F6749 hits |
| .....  Escherichia coli O157:H7 str. F6751 | 1 | 1 | Escherichia coli O157:H7 str. F6751 hits |
| .....  Escherichia coli O157:H7 str. F7384 | 1 | 1 | Escherichia coli O157:H7 str. F7384 hits |
| .....  Escherichia coli O157:H7 str. F7410 | 1 | 1 | Escherichia coli O157:H7 str. F7410 hits |
| .....  Escherichia coli O157:H7 str. G5303 | 1 | 1 | Escherichia coli O157:H7 str. G5303 hits |
| .....  Escherichia coli O157:H7 str. H2495 | 1 | 1 | Escherichia coli O157:H7 str. H2495 hits |
| .....  Escherichia coli O157:H7 str. K1420 | 1 | 1 | Escherichia coli O157:H7 str. K1420 hits |
| .....  Escherichia coli O157:H7 str. H2498 | 1 | 1 | Escherichia coli O157:H7 str. H2498 hits |
| .....  Escherichia coli O157:H7 str. K1792 | 1 | 1 | Escherichia coli O157:H7 str. K1792 hits |
| .....  Escherichia coli O157:H7 str. K1845 | 1 | 1 | Escherichia coli O157:H7 str. K1845 hits |
| .....  Escherichia coli O157:H7 str. K1796 | 1 | 1 | Escherichia coli O157:H7 str. K1796 hits |
| .....  Escherichia coli O157:H7 str. K1921 | 1 | 1 | Escherichia coli O157:H7 str. K1921 hits |
| .....  Escherichia coli O157:H7 str. K2188 | 1 | 1 | Escherichia coli O157:H7 str. K2188 hits |
| .....  Escherichia coli O157:H7 str. K2191 | 1 | 1 | Escherichia coli O157:H7 str. K2191 hits |
| .....  Escherichia coli O157:H7 str. K2192 | 1 | 1 | Escherichia coli O157:H7 str. K2192 hits |
| .....  Escherichia coli O157:H7 str. K2581 | 1 | 1 | Escherichia coli O157:H7 str. K2581 hits |
| .....  Escherichia coli O157:H7 str. K2622 | 1 | 1 | Escherichia coli O157:H7 str. K2622 hits |
| .....  Escherichia coli O157:H7 str. K2845 | 1 | 1 | Escherichia coli O157:H7 str. K2845 hits |
| .....  Escherichia coli O157:H7 str. K2854 | 1 | 1 | Escherichia coli O157:H7 str. K2854 hits |
| .....  Escherichia coli O157:H7 str. K4406 | 1 | 1 | Escherichia coli O157:H7 str. K4406 hits |
| .....  Escherichia coli O157:H7 str. K4396 | 1 | 1 | Escherichia coli O157:H7 str. K4396 hits |
| .....  Escherichia coli O157:H7 str. K4405 | 1 | 1 | Escherichia coli O157:H7 str. K4405 hits |
| .....  Escherichia coli O157:H7 str. K4527 | 1 | 1 | Escherichia coli O157:H7 str. K4527 hits |
| .....  Escherichia coli O157:H7 str. K5418 | 1 | 1 | Escherichia coli O157:H7 str. K5418 hits |
| .....  Escherichia coli O157:H7 str. K5448 | 1 | 1 | Escherichia coli O157:H7 str. K5448 hits |
| .....  Escherichia coli O157:H7 str. K5453 | 1 | 1 | Escherichia coli O157:H7 str. K5453 hits |
| .....  Escherichia coli O157:H7 str. K5449 | 1 | 1 | Escherichia coli O157:H7 str. K5449 hits |
| .....  Escherichia coli O157:H7 str. K5460 | 1 | 1 | Escherichia coli O157:H7 str. K5460 hits |
| .....  Escherichia coli O157:H7 str. K5467 | 1 | 1 | Escherichia coli O157:H7 str. K5467 hits |
| .....  Escherichia coli O157:H7 str. K5602 | 1 | 1 | Escherichia coli O157:H7 str. K5602 hits |
| .....  Escherichia coli O157:H7 str. K5609 | 1 | 1 | Escherichia coli O157:H7 str. K5609 hits |
| .....  Escherichia coli O157:H7 str. K5607 | 1 | 1 | Escherichia coli O157:H7 str. K5607 hits |
| .....  Escherichia coli O157:H7 str. K5852 | 1 | 1 | Escherichia coli O157:H7 str. K5852 hits |
| .....  Escherichia coli O157:H7 str. K6590 | 1 | 1 | Escherichia coli O157:H7 str. K6590 hits |
| .....  Escherichia coli O157:H7 str. K6676 | 1 | 1 | Escherichia coli O157:H7 str. K6676 hits |
| .....  Escherichia coli O157:H7 str. K6687 | 1 | 1 | Escherichia coli O157:H7 str. K6687 hits |
| .....  Escherichia coli O157:H7 str. K7140 | 1 | 1 | Escherichia coli O157:H7 str. K7140 hits |
| .....  Escherichia coli O157:H7 str. 08-4529 | 1 | 1 | Escherichia coli O157:H7 str. 08-4529 hits |
| .....  Escherichia coli O157:H7 str. 2009EL1913 | 1 | 1 | Escherichia coli O157:H7 str. 2009EL1913 hits |
| .....  Escherichia coli O157:H7 str. 2011EL-2313 | 1 | 1 | Escherichia coli O157:H7 str. 2011EL-2313 hits |
| .....  Escherichia coli O157:H7 str. 2011EL-2290 | 1 | 1 | Escherichia coli O157:H7 str. 2011EL-2290 hits |
| .....  Escherichia coli O157:H7 str. EC1825 | 1 | 1 | Escherichia coli O157:H7 str. EC1825 hits |
| .....  Escherichia coli O157:H7 str. K1927 | 1 | 1 | Escherichia coli O157:H7 str. K1927 hits |
| .....  Escherichia coli O157:H7 str. 2009EL1449 | 1 | 1 | Escherichia coli O157:H7 str. 2009EL1449 hits |
| .....  Escherichia coli O157:H7 str. K2324 | 1 | 1 | Escherichia coli O157:H7 str. K2324 hits |
| ....  Escherichia coli 6-175-07\_S1\_C1 | 1 | 1 | Escherichia coli 6-175-07\_S1\_C1 hits |
| ....  Escherichia coli T1282\_01 | 1 | 1 | Escherichia coli T1282\_01 hits |
| ....  Escherichia coli 99.1775 | 1 | 1 | Escherichia coli 99.1775 hits |
| ....  Escherichia coli 99.1781 | 1 | 1 | Escherichia coli 99.1781 hits |
| ....  Escherichia coli 96.0939 | 1 | 1 | Escherichia coli 96.0939 hits |
| ....  Escherichia coli FRIK1996 | 1 | 1 | Escherichia coli FRIK1996 hits |
| ....  Escherichia coli FRIK1990 | 1 | 1 | Escherichia coli FRIK1990 hits |
| ....  Escherichia coli PA40 | 1 | 1 | Escherichia coli PA40 hits |
| ....  Escherichia coli PA41 | 1 | 1 | Escherichia coli PA41 hits |
| ....  Escherichia coli TW09195 | 1 | 1 | Escherichia coli TW09195 hits |
| ....  Escherichia coli FRIK920 | 1 | 1 | Escherichia coli FRIK920 hits |
| ....  Escherichia coli FRIK1999 | 1 | 1 | Escherichia coli FRIK1999 hits |
| ....  Escherichia coli NE1487 | 1 | 1 | Escherichia coli NE1487 hits |
| ....  Escherichia coli FRIK2001 | 1 | 1 | Escherichia coli FRIK2001 hits |
| ....  Escherichia coli MA6 | 1 | 1 | Escherichia coli MA6 hits |
| ....  Escherichia coli 90.0039 | 1 | 1 | Escherichia coli 90.0039 hits |
| ....  Escherichia coli 95.0183 | 1 | 1 | Escherichia coli 95.0183 hits |
| ....  Escherichia coli 99.0672 | 1 | 1 | Escherichia coli 99.0672 hits |
| ....  Escherichia coli O157 | 2 | 11 | Escherichia coli O157 hits |
| .....  Escherichia coli ATCC 700728 | 1 | 1 | Escherichia coli ATCC 700728 hits |
| .....  Escherichia coli PA11 | 1 | 1 | Escherichia coli PA11 hits |
| .....  Escherichia coli PA13 | 1 | 1 | Escherichia coli PA13 hits |
| .....  Escherichia coli PA19 | 1 | 1 | Escherichia coli PA19 hits |
| .....  Escherichia coli 7.1982 | 1 | 1 | Escherichia coli 7.1982 hits |
| .....  Escherichia coli PA35 | 1 | 1 | Escherichia coli PA35 hits |
| .....  Escherichia coli 3.4880 | 1 | 1 | Escherichia coli 3.4880 hits |
| .....  Escherichia coli 95.0083 | 1 | 1 | Escherichia coli 95.0083 hits |
| .....  Escherichia coli O157: str. 2010EL-2045 | 1 | 1 | Escherichia coli O157: str. 2010EL-2045 hits |
| .....  Escherichia coli O157: str. 2010EL-2044 | 1 | 1 | Escherichia coli O157: str. 2010EL-2044 hits |
| ....  Escherichia coli 08BKT055439 | 1 | 1 | Escherichia coli 08BKT055439 hits |
| ....  Escherichia coli T234\_00 | 1 | 1 | Escherichia coli T234\_00 hits |
| ....  Escherichia coli 14A | 1 | 1 | Escherichia coli 14A hits |
| ....  Escherichia coli 08BKT77219 | 1 | 1 | Escherichia coli 08BKT77219 hits |
| ....  Escherichia coli O55:H7 | 8 | 6 |  |
| .....  Escherichia coli O55:H7 str. RM12579 | 1 | 1 | Escherichia coli O55:H7 str. RM12579 hits |
| .....  Escherichia coli O55:H7 str. TB182A | 1 | 1 | Escherichia coli O55:H7 str. TB182A hits |
| .....  Escherichia coli O55:H7 str. USDA 5905 | 2 | 1 | Escherichia coli O55:H7 str. USDA 5905 hits |
| .....  Escherichia coli O55:H7 str. CB9615 | 2 | 1 | Escherichia coli O55:H7 str. CB9615 hits |
| .....  Escherichia coli O55:H7 str. 3256-97 | 1 | 1 | Escherichia coli O55:H7 str. 3256-97 hits |
| .....  Escherichia coli O55:H7 str. 06-3555 | 1 | 1 | Escherichia coli O55:H7 str. 06-3555 hits |
| ....  Escherichia coli DEC5C | 1 | 1 | Escherichia coli DEC5C hits |
| ....  Escherichia coli DEC5D | 1 | 1 | Escherichia coli DEC5D hits |
| ....  Escherichia coli 99.0814 | 1 | 1 | Escherichia coli 99.0814 hits |
| ....  Escherichia coli 99.0815 | 1 | 1 | Escherichia coli 99.0815 hits |
| ....  Escherichia coli 99.0816 | 1 | 1 | Escherichia coli 99.0816 hits |
| ....  Escherichia coli 95.0943 | 1 | 1 | Escherichia coli 95.0943 hits |
| ....  Escherichia coli 99.1753 | 1 | 1 | Escherichia coli 99.1753 hits |
| ....  Escherichia coli B107 | 1 | 1 | Escherichia coli B107 hits |
| ....  Escherichia coli B102 | 1 | 1 | Escherichia coli B102 hits |
| ....  Escherichia coli B26-1 | 1 | 1 | Escherichia coli B26-1 hits |
| ....  Escherichia coli B26-2 | 1 | 1 | Escherichia coli B26-2 hits |
| ....  Escherichia coli B103 | 1 | 1 | Escherichia coli B103 hits |
| ....  Escherichia coli B104 | 1 | 1 | Escherichia coli B104 hits |
| ....  Escherichia coli B105 | 1 | 1 | Escherichia coli B105 hits |
| ....  Escherichia coli B106 | 1 | 1 | Escherichia coli B106 hits |
| ....  Escherichia coli B40-2 | 1 | 1 | Escherichia coli B40-2 hits |
| ....  Escherichia coli B40-1 | 1 | 1 | Escherichia coli B40-1 hits |
| ....  Escherichia coli B49-2 | 1 | 1 | Escherichia coli B49-2 hits |
| ....  Escherichia coli B83 | 1 | 1 | Escherichia coli B83 hits |
| ....  Escherichia coli B84 | 1 | 1 | Escherichia coli B84 hits |
| ....  Escherichia coli B85 | 1 | 1 | Escherichia coli B85 hits |
| ....  Escherichia coli B86 | 1 | 1 | Escherichia coli B86 hits |
| ....  Escherichia coli O145:NM | 1 | 14 | Escherichia coli O145:NM hits |
| .....  Escherichia coli O145:NM str. 2010C-4557C2 | 1 | 1 | Escherichia coli O145:NM str. 2010C-4557C2 hits |
| .....  Escherichia coli O145:NM str. 2010C-3526 | 1 | 1 | Escherichia coli O145:NM str. 2010C-3526 hits |
| .....  Escherichia coli O145:NM str. 2010C-3521 | 1 | 1 | Escherichia coli O145:NM str. 2010C-3521 hits |
| .....  Escherichia coli O145:NM str. 2010C-3517 | 1 | 1 | Escherichia coli O145:NM str. 2010C-3517 hits |
| .....  Escherichia coli O145:NM str. 2010C-3518 | 1 | 1 | Escherichia coli O145:NM str. 2010C-3518 hits |
| .....  Escherichia coli O145:NM str. 2010C-3516 | 1 | 1 | Escherichia coli O145:NM str. 2010C-3516 hits |
| .....  Escherichia coli O145:NM str. 2010C-3510 | 1 | 1 | Escherichia coli O145:NM str. 2010C-3510 hits |
| .....  Escherichia coli O145:NM str. 2010C-3509 | 1 | 1 | Escherichia coli O145:NM str. 2010C-3509 hits |
| .....  Escherichia coli O145:NM str. 2010C-3511 | 1 | 1 | Escherichia coli O145:NM str. 2010C-3511 hits |
| .....  Escherichia coli O145:NM str. 2010C-3507 | 1 | 1 | Escherichia coli O145:NM str. 2010C-3507 hits |
| .....  Escherichia coli O145:NM str. 08-4270 | 1 | 1 | Escherichia coli O145:NM str. 08-4270 hits |
| .....  Escherichia coli O145:NM str. 06-3484 | 1 | 1 | Escherichia coli O145:NM str. 06-3484 hits |
| .....  Escherichia coli O145:NM str. 2010C-3508 | 1 | 1 | Escherichia coli O145:NM str. 2010C-3508 hits |
| ....  Escherichia coli O145:H28 | 5 | 5 |  |
| .....  Escherichia coli O145:H28 str. RM13514 | 1 | 1 | Escherichia coli O145:H28 str. RM13514 hits |
| .....  Escherichia coli O145:H28 str. RM13516 | 1 | 1 | Escherichia coli O145:H28 str. RM13516 hits |
| .....  Escherichia coli O145:H28 str. RM12761 | 1 | 1 | Escherichia coli O145:H28 str. RM12761 hits |
| .....  Escherichia coli O145:H28 str. RM12581 | 1 | 1 | Escherichia coli O145:H28 str. RM12581 hits |
| .....  Escherichia coli O145:H28 str. 4865/96 | 1 | 1 | Escherichia coli O145:H28 str. 4865/96 hits |
| ....  Escherichia coli O145 | 1 | 2 | Escherichia coli O145 hits |
| .....  Escherichia coli O145 str. RM9872 | 1 | 1 | Escherichia coli O145 str. RM9872 hits |
| ....  Escherichia coli O157:H- | 2 | 2 |  |
| .....  Escherichia coli O157:H- str. 493-89 | 1 | 1 | Escherichia coli O157:H- str. 493-89 hits |
| .....  Escherichia coli O157:H- str. H 2687 | 1 | 1 | Escherichia coli O157:H- str. H 2687 hits |
| ....  Escherichia coli DEC3F | 1 | 1 | Escherichia coli DEC3F hits |
| ....  Escherichia coli TW06591 | 1 | 1 | Escherichia coli TW06591 hits |
| ....  Escherichia coli 5412 | 1 | 1 | Escherichia coli 5412 hits |
| ....  Escherichia coli TW10119 | 1 | 1 | Escherichia coli TW10119 hits |
| ....  Escherichia coli 90.0091 | 1 | 1 | Escherichia coli 90.0091 hits |
| ....  Escherichia coli 99.1805 | 1 | 1 | Escherichia coli 99.1805 hits |
| ....  Escherichia coli O157:NM str. 08-4540 | 1 | 1 | Escherichia coli O157:NM str. 08-4540 hits |
| ....  Escherichia coli Xuzhou21 | 1 | 1 | Escherichia coli Xuzhou21 hits |
| ....  Escherichia coli DEC3A | 1 | 1 | Escherichia coli DEC3A hits |
| ....  Escherichia coli DEC3B | 1 | 1 | Escherichia coli DEC3B hits |
| ....  Escherichia coli DEC3C | 1 | 1 | Escherichia coli DEC3C hits |
| ....  Escherichia coli DEC4E | 1 | 1 | Escherichia coli DEC4E hits |
| ....  Escherichia coli DEC4F | 1 | 1 | Escherichia coli DEC4F hits |
| ....  Escherichia coli FDA505 | 1 | 1 | Escherichia coli FDA505 hits |
| ....  Escherichia coli FDA517 | 1 | 1 | Escherichia coli FDA517 hits |
| ....  Escherichia coli FRIK1985 | 1 | 1 | Escherichia coli FRIK1985 hits |
| ....  Escherichia coli 93-001 | 1 | 1 | Escherichia coli 93-001 hits |
| ....  Escherichia coli PA3 | 1 | 1 | Escherichia coli PA3 hits |
| ....  Escherichia coli PA5 | 1 | 1 | Escherichia coli PA5 hits |
| ....  Escherichia coli PA9 | 1 | 1 | Escherichia coli PA9 hits |
| ....  Escherichia coli PA10 | 1 | 1 | Escherichia coli PA10 hits |
| ....  Escherichia coli PA14 | 1 | 1 | Escherichia coli PA14 hits |
| ....  Escherichia coli PA15 | 1 | 1 | Escherichia coli PA15 hits |
| ....  Escherichia coli PA25 | 1 | 1 | Escherichia coli PA25 hits |
| ....  Escherichia coli PA24 | 1 | 1 | Escherichia coli PA24 hits |
| ....  Escherichia coli PA28 | 1 | 1 | Escherichia coli PA28 hits |
| ....  Escherichia coli PA31 | 1 | 1 | Escherichia coli PA31 hits |
| ....  Escherichia coli PA32 | 1 | 1 | Escherichia coli PA32 hits |
| ....  Escherichia coli PA33 | 1 | 1 | Escherichia coli PA33 hits |
| ....  Escherichia coli PA39 | 1 | 1 | Escherichia coli PA39 hits |
| ....  Escherichia coli PA42 | 1 | 1 | Escherichia coli PA42 hits |
| ....  Escherichia coli TW07945 | 1 | 1 | Escherichia coli TW07945 hits |
| ....  Escherichia coli TW09098 | 1 | 1 | Escherichia coli TW09098 hits |
| ....  Escherichia coli TW09109 | 1 | 1 | Escherichia coli TW09109 hits |
| ....  Escherichia coli EC4203 | 1 | 1 | Escherichia coli EC4203 hits |
| ....  Escherichia coli EC4196 | 1 | 1 | Escherichia coli EC4196 hits |
| ....  Escherichia coli TW14301 | 1 | 1 | Escherichia coli TW14301 hits |
| ....  Escherichia coli EC4421 | 1 | 1 | Escherichia coli EC4421 hits |
| ....  Escherichia coli EC4422 | 1 | 1 | Escherichia coli EC4422 hits |
| ....  Escherichia coli EC4013 | 1 | 1 | Escherichia coli EC4013 hits |
| ....  Escherichia coli EC4402 | 1 | 1 | Escherichia coli EC4402 hits |
| ....  Escherichia coli EC4439 | 1 | 1 | Escherichia coli EC4439 hits |
| ....  Escherichia coli EC4436 | 1 | 1 | Escherichia coli EC4436 hits |
| ....  Escherichia coli EC4437 | 1 | 1 | Escherichia coli EC4437 hits |
| ....  Escherichia coli EC4448 | 1 | 1 | Escherichia coli EC4448 hits |
| ....  Escherichia coli EC1738 | 1 | 1 | Escherichia coli EC1738 hits |
| ....  Escherichia coli EC1734 | 1 | 1 | Escherichia coli EC1734 hits |
| ....  Escherichia coli EC1863 | 1 | 1 | Escherichia coli EC1863 hits |
| ....  Escherichia coli EC1845 | 1 | 1 | Escherichia coli EC1845 hits |
| ....  Escherichia coli PA7 | 1 | 1 | Escherichia coli PA7 hits |
| ....  Escherichia coli PA34 | 1 | 1 | Escherichia coli PA34 hits |
| ....  Escherichia coli FDA506 | 1 | 1 | Escherichia coli FDA506 hits |
| ....  Escherichia coli FDA507 | 1 | 1 | Escherichia coli FDA507 hits |
| ....  Escherichia coli FDA504 | 1 | 1 | Escherichia coli FDA504 hits |
| ....  Escherichia coli NE037 | 1 | 1 | Escherichia coli NE037 hits |
| ....  Escherichia coli PA4 | 1 | 1 | Escherichia coli PA4 hits |
| ....  Escherichia coli PA23 | 1 | 1 | Escherichia coli PA23 hits |
| ....  Escherichia coli PA49 | 1 | 1 | Escherichia coli PA49 hits |
| ....  Escherichia coli PA45 | 1 | 1 | Escherichia coli PA45 hits |
| ....  Escherichia coli TT12B | 1 | 1 | Escherichia coli TT12B hits |
| ....  Escherichia coli CB7326 | 1 | 1 | Escherichia coli CB7326 hits |
| ....  Escherichia coli EC96038 | 1 | 1 | Escherichia coli EC96038 hits |
| ....  Escherichia coli PA38 | 1 | 1 | Escherichia coli PA38 hits |
| ....  Escherichia coli EC1736 | 1 | 1 | Escherichia coli EC1736 hits |
| ....  Escherichia coli EC1846 | 1 | 1 | Escherichia coli EC1846 hits |
| ....  Escherichia coli EC1847 | 1 | 1 | Escherichia coli EC1847 hits |
| ....  Escherichia coli EC1848 | 1 | 1 | Escherichia coli EC1848 hits |
| ....  Escherichia coli EC1849 | 1 | 1 | Escherichia coli EC1849 hits |
| ....  Escherichia coli EC1850 | 1 | 1 | Escherichia coli EC1850 hits |
| ....  Escherichia coli EC1856 | 1 | 1 | Escherichia coli EC1856 hits |
| ....  Escherichia coli EC1862 | 1 | 1 | Escherichia coli EC1862 hits |
| ....  Escherichia coli EC1864 | 1 | 1 | Escherichia coli EC1864 hits |
| ....  Escherichia coli EC1868 | 1 | 1 | Escherichia coli EC1868 hits |
| ....  Escherichia coli EC1866 | 1 | 1 | Escherichia coli EC1866 hits |
| ....  Escherichia coli EC1869 | 1 | 1 | Escherichia coli EC1869 hits |
| ....  Escherichia coli EC1870 | 1 | 1 | Escherichia coli EC1870 hits |
| ....  Escherichia coli NE098 | 1 | 1 | Escherichia coli NE098 hits |
| ....  Escherichia coli FRIK523 | 1 | 1 | Escherichia coli FRIK523 hits |
| ....  Escherichia coli 0.1304 | 1 | 1 | Escherichia coli 0.1304 hits |
| ....  Escherichia coli 5.2239 | 1 | 1 | Escherichia coli 5.2239 hits |
| ....  Escherichia coli 3.4870 | 1 | 1 | Escherichia coli 3.4870 hits |
| ....  Escherichia coli 6.0172 | 1 | 1 | Escherichia coli 6.0172 hits |
| ....  Escherichia coli 8.0586 | 1 | 1 | Escherichia coli 8.0586 hits |
| ....  Escherichia coli 10.0833 | 1 | 1 | Escherichia coli 10.0833 hits |
| ....  Escherichia coli 8.2524 | 1 | 1 | Escherichia coli 8.2524 hits |
| ....  Escherichia coli 10.0869 | 1 | 1 | Escherichia coli 10.0869 hits |
| ....  Escherichia coli 10.0821 | 1 | 1 | Escherichia coli 10.0821 hits |
| ....  Escherichia coli 88.1042 | 1 | 1 | Escherichia coli 88.1042 hits |
| ....  Escherichia coli 89.0511 | 1 | 1 | Escherichia coli 89.0511 hits |
| ....  Escherichia coli 90.2281 | 1 | 1 | Escherichia coli 90.2281 hits |
| ....  Escherichia coli 93.0056 | 1 | 1 | Escherichia coli 93.0056 hits |
| ....  Escherichia coli 93.0055 | 1 | 1 | Escherichia coli 93.0055 hits |
| ....  Escherichia coli 94.0618 | 1 | 1 | Escherichia coli 94.0618 hits |
| ....  Escherichia coli 95.1288 | 1 | 1 | Escherichia coli 95.1288 hits |
| ....  Escherichia coli 96.0428 | 1 | 1 | Escherichia coli 96.0428 hits |
| ....  Escherichia coli 96.0427 | 1 | 1 | Escherichia coli 96.0427 hits |
| ....  Escherichia coli 96.0932 | 1 | 1 | Escherichia coli 96.0932 hits |
| ....  Escherichia coli 96.0107 | 1 | 1 | Escherichia coli 96.0107 hits |
| ....  Escherichia coli 97.0003 | 1 | 1 | Escherichia coli 97.0003 hits |
| ....  Escherichia coli 97.0007 | 1 | 1 | Escherichia coli 97.0007 hits |
| ....  Escherichia coli 99.0678 | 1 | 1 | Escherichia coli 99.0678 hits |
| ....  Escherichia coli 99.0713 | 1 | 1 | Escherichia coli 99.0713 hits |
| ....  Escherichia coli 96.0109 | 1 | 1 | Escherichia coli 96.0109 hits |
| ....  Escherichia coli 97.0010 | 1 | 1 | Escherichia coli 97.0010 hits |
| ....  Escherichia coli 09BKT078844 | 1 | 1 | Escherichia coli 09BKT078844 hits |
| ....  Escherichia coli 99.0839 | 1 | 1 | Escherichia coli 99.0839 hits |
| ....  Escherichia coli 99.0848 | 1 | 1 | Escherichia coli 99.0848 hits |
| ....  Escherichia coli PA2 | 1 | 1 | Escherichia coli PA2 hits |
| ....  Escherichia coli PA47 | 1 | 1 | Escherichia coli PA47 hits |
| ....  Escherichia coli PA48 | 1 | 1 | Escherichia coli PA48 hits |
| ....  Escherichia coli PA8 | 1 | 1 | Escherichia coli PA8 hits |
| ....  Escherichia coli 99.1762 | 1 | 1 | Escherichia coli 99.1762 hits |
| ....  Escherichia coli 99.0670 | 1 | 1 | Escherichia coli 99.0670 hits |
| ....  Escherichia coli B28-1 | 1 | 1 | Escherichia coli B28-1 hits |
| ....  Escherichia coli B28-2 | 1 | 1 | Escherichia coli B28-2 hits |
| ....  Escherichia coli B29-1 | 1 | 1 | Escherichia coli B29-1 hits |
| ....  Escherichia coli B29-2 | 1 | 1 | Escherichia coli B29-2 hits |
| ....  Escherichia coli B36-1 | 1 | 1 | Escherichia coli B36-1 hits |
| ....  Escherichia coli B36-2 | 1 | 1 | Escherichia coli B36-2 hits |
| ....  Escherichia coli B7-1 | 1 | 1 | Escherichia coli B7-1 hits |
| ....  Escherichia coli B7-2 | 1 | 1 | Escherichia coli B7-2 hits |
| ....  Escherichia coli B93 | 1 | 1 | Escherichia coli B93 hits |
| ....  Escherichia coli B94 | 1 | 1 | Escherichia coli B94 hits |
| ....  Escherichia coli B95 | 1 | 1 | Escherichia coli B95 hits |
| ....  Escherichia coli Bd5610\_99 | 1 | 1 | Escherichia coli Bd5610\_99 hits |
| ....  Escherichia coli T1840\_97 | 1 | 1 | Escherichia coli T1840\_97 hits |
| ....  Escherichia coli T924\_01 | 1 | 1 | Escherichia coli T924\_01 hits |
| ....  Escherichia coli 2886-75 | 1 | 1 | Escherichia coli 2886-75 hits |
| ....  Escherichia coli B108 | 1 | 1 | Escherichia coli B108 hits |
| ....  Escherichia coli B109 | 1 | 1 | Escherichia coli B109 hits |
| ....  Escherichia coli B112 | 1 | 1 | Escherichia coli B112 hits |
| ....  Escherichia coli B113 | 1 | 1 | Escherichia coli B113 hits |
| ....  Escherichia coli B114 | 1 | 1 | Escherichia coli B114 hits |
| ....  Escherichia coli B15 | 1 | 1 | Escherichia coli B15 hits |
| ....  Escherichia coli B17 | 1 | 1 | Escherichia coli B17 hits |
| ....  Escherichia coli B5-2 | 1 | 1 | Escherichia coli B5-2 hits |
| ....  Escherichia coli 09BKT024447 | 1 | 1 | Escherichia coli 09BKT024447 hits |
| ....  Escherichia coli B89 | 1 | 1 | Escherichia coli B89 hits |
| ....  Escherichia coli B90 | 1 | 1 | Escherichia coli B90 hits |
| ....  Escherichia coli Tx1686 | 1 | 1 | Escherichia coli Tx1686 hits |
| ....  Escherichia coli Tx3800 | 1 | 1 | Escherichia coli Tx3800 hits |
| ....  Escherichia coli ATCC BAA-2192 | 1 | 1 | Escherichia coli ATCC BAA-2192 hits |
| ....  Escherichia coli SHECO003 | 1 | 1 | Escherichia coli SHECO003 hits |
| ....  Escherichia coli DEC5E | 1 | 1 | Escherichia coli DEC5E hits |
| ....  Escherichia coli 5905 | 1 | 1 | Escherichia coli 5905 hits |
| ....  Escherichia coli 09BKT076207 | 1 | 1 | Escherichia coli 09BKT076207 hits |
| ....  Escherichia coli DEC4A | 1 | 1 | Escherichia coli DEC4A hits |
| ....  Escherichia coli DEC5A | 1 | 1 | Escherichia coli DEC5A hits |
| ....  Escherichia coli DEC5B | 1 | 1 | Escherichia coli DEC5B hits |
| ....  Escherichia coli PA22 | 1 | 1 | Escherichia coli PA22 hits |
| ....  Escherichia coli TW10246 | 1 | 1 | Escherichia coli TW10246 hits |
| ....  Escherichia coli 8.0416 | 1 | 1 | Escherichia coli 8.0416 hits |
| ....  Escherichia coli 88.1467 | 1 | 1 | Escherichia coli 88.1467 hits |
| ....  Escherichia coli TW11039 | 1 | 1 | Escherichia coli TW11039 hits |
| ....  Escherichia coli FRIK1997 | 1 | 1 | Escherichia coli FRIK1997 hits |
| ....  Escherichia coli EC1735 | 1 | 1 | Escherichia coli EC1735 hits |
| ....  Escherichia coli EC1737 | 1 | 1 | Escherichia coli EC1737 hits |
| ....  Escherichia coli 88.0221 | 1 | 1 | Escherichia coli 88.0221 hits |
| ....  Escherichia coli DEC4B | 1 | 1 | Escherichia coli DEC4B hits |
| ....  Escherichia coli ATCC 35150 | 1 | 1 | Escherichia coli ATCC 35150 hits |
| ....  Escherichia coli 97.1742 | 1 | 1 | Escherichia coli 97.1742 hits |
| ....  Escherichia coli DEC4D | 1 | 1 | Escherichia coli DEC4D hits |
| ....  Escherichia coli DEC3D | 1 | 1 | Escherichia coli DEC3D hits |
| ....  Escherichia coli DEC3E | 1 | 1 | Escherichia coli DEC3E hits |
| ....  Escherichia coli DEC4C | 1 | 1 | Escherichia coli DEC4C hits |
| ..  Shigella | 4 | 2 |  |
| ...  Shigella boydii | 3 | 1 | Shigella boydii hits |
| ...  Shigella dysenteriae | 1 | 1 | Shigella dysenteriae hits |
| ..  Salmonella enterica subsp. enterica | 1 | 1 | Salmonella enterica subsp. enterica hits |
| .  synthetic construct | 2 | 1 | synthetic construct hits |


BLAST is a registered trademark of the National Library of Medicine

Support center
Mailing list


YouTube

- National Library Of Medicine
- National Institutes Of Health
- U.S. Department of Health & Human Services
- USA.gov

### NCBI


National Center for Biotechnology Information,
 U.S. National Library of Medicine

8600 Rockville Pike,
Bethesda
 MD,
20894
USA

Policies and Guidelines
|
Contact
